# Molecular Insights into the Aroma Difference between Beer and Wine: A Meta-Analysis-Based Sensory Study Using Concentration Leveling Tests

**DOI:** 10.1021/acs.jafc.4c06838

**Published:** 2024-09-30

**Authors:** Xingjie Wang, Stephanie Frank, Martin Steinhaus

**Affiliations:** † Technical University of Munich, TUM School of Natural Sciences,Department of Chemistry, Lichtenbergstraße 4, 85748 Garching, Germany; ‡ Leibniz Institute for Food Systems Biology at the Technical University of Munich (Leibniz-LSB@TUM), Lise-Meitner-Straße 34, 85354 Freising, Germany

**Keywords:** beer, wine, aroma base, fruity odorants, literature survey, meta-analysis, concentration leveling test

## Abstract

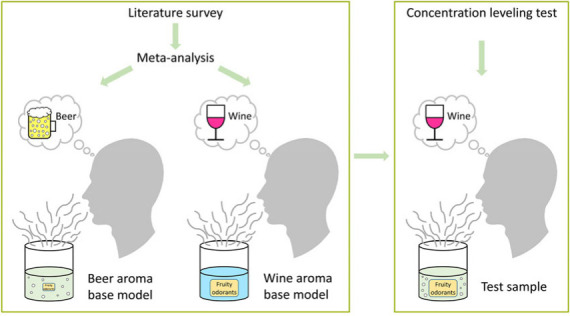

Beer and wine are popular beverages with clearly different aroma characters, the molecular background of which has not yet been systematically investigated. A comprehensive literature survey returned 14 845 concentration values obtained from 160 beer and 904 wine samples, covering 42 basic beer and 42 basic wine odorants, among which 40 were common to both beverages. Based on mean concentrations and a comparison with threshold data, 29 beer and 32 wine odorants were finally selected to build aroma base models that reflected the basic olfactory difference between beer and wine. Orthonasal concentration leveling tests applied to groups of odorants with similar odor characteristics finally revealed the crucial role of fruity smelling compounds. When 11 fruity compounds, predominantly esters, in the beer aroma base model were adjusted to the respective concentration levels in the wine aroma base model, the sensory panel no longer described the sample as beer-like but as wine-like.

## Introduction

With production volumes of 189 and 26.2 billion liters in 2022, beer and wine are undoubtedly the most important alcoholic beverages in the world.^1,2^ Whereas winemaking has a history of approximately 8000 years, humankind started even earlier making beer, presumably around 13 000 bp.^3,4^ An important factor that adds to the popularity of beer and wine is their pleasant aroma, which is basically caused by volatile compounds. In both beer and wine, several hundred volatiles have been structurally characterized so far.^5^ However, only the volatiles present in concentrations above their respective substance-specific odor threshold concentrations (OTCs) have the potential to contribute to the aroma, which considerably reduces the number of compounds.^6^ Among these odor-active volatiles are compounds originating in the starting materials as well as compounds formed during processing and storage.^7−21^ For example, malt^7,8^ and hops^9−11^ are important sources of beer odorants, and so are grapes for wine odorants.^12−17^ A major proportion of beer and wine odorants, however, are formed during fermentation.^12,14,18−20^ This contributes to the fact that beer and wine have many key odorants in common, which in turn raises the question of which compounds actually account for the aroma difference between beer and wine. In his groundbreaking work on wine aroma, Vicente Ferreira defined an “aroma base” consisting of ethanol and 26 other, mainly also fermentation-derived compounds that produced a basic wine aroma.^22^ Further odorants, either individually or in combination, can, if present in sufficient concentration, break the “aroma buffer” formed by the aroma base odorants and generate specific aroma notes, including the woody character of barrel-aged wines^23−25^ and specific varietal notes such as the 1,1,6-trimethyl-1,2-dihydronaphthalene (TDN) note specifically for Riesling wines.^16,17^ No such research was ever undertaken with beer aroma compounds.

The aims of our study were to (i) perform a literature-based meta-analysis on the occurrence and concentrations of beer and wine odorants, (ii) based on the literature data establish aroma base models for beer and wine that reflect the basic olfactory difference between the two, and (iii) identify the odorant group(s) responsible for the aroma difference between the beer and wine base models by sensory analyses using concentration leveling tests.

## Materials and Methods

### Literature Survey and Data Extraction

Web of Science and Google Scholar database searches were conducted using the strings “aroma”, “volatile”, “odor-active compound”, and “odorant” in combination with “beer” and “wine”, respectively. Papers written in English and published in 2000 or later were screened for odorant concentration data. Data extraction was applied to papers that fulfilled the following sample and quality criteria: (1) Beer data were obtained from bottom-fermented beers predominantly brewed with barley malt; wine data were obtained from dry red, rosé, or white wines exclusively made from *Vitis vinifera* berries. (2) Fermentation was accomplished with a single commercial *Saccharomyce*s yeast strain. (3) The ethanol concentration was within typical limits (3.5–7.5% ALC/VOL in beer, 8.5–17.9% ALC/VOL in wine). And finally, (4) structure assignments complied with the JAFC guidelines for reporting flavor constituents, and, in addition, quantitation methods used GC–FID, GC–MS, or LC–MS, included the use of internal standards, and compensated for detector response differences by appropriate calibration approaches. Data not further considered included, for example, data obtained from light and extra high alcohol beers, dealcoholized beers and wines, data obtained from beers and wines made with mixed fermentation, and data obtained after storage at elevated temperature.

### Beer and Wine Samples

German Bitburger Premium Pils beer (4.8% ALC/VOL; vintage 2022), Spanish Airén dry white wine (10.5% ALC/VOL; vintage 2021), German Silvaner dry white wine (12.0% ALC/VOL; vintage 2019), and German Weissburgunder dry white wine (12.0% ALC/VOL; vintage 2021) were purchased from a local shop in Germany.

### Reference Odorants

Commercially available compounds were purchased at the highest purity available. Acetaldehyde (≥99%), acetic acid (≥99%), butane-2,3-dione (97%), decanoic acid (≥98%), 1,1-diethoxyethane (99%), dimethyl sulfide (≥99%), ethyl acetate (≥99%), ethyl butanoate (≥99%), ethyl decanoate (≥99%), ethyl hexanoate (≥99%), ethyl 2-methylbutanoate (99%), ethyl 3-methylbutanoate (98%), ethyl 2-methylpropanoate (≥98%), ethyl octanoate (≥98%), 3-hydroxybutan-2-one (97%), 2-methylbutanal (95%), 3-methylbutanal (≥97%), 3-methylbutanoic acid (99%), 2-methylbutan-1-ol (≥99%), 3-methylbutan-1-ol (≥98%), 3-methylbutyl acetate (≥99%), 2-methylpropanal (≥99%), 2-methylpropyl acetate (99%), 3-(methylsulfanyl)propanal (96%), 3-(methylsulfanyl)propan-1-ol (≥98%), octanoic acid (≥98%), phenylacetic acid (99%), 2-phenylethan-1-ol (≥99%) were from Merck (Darmstadt, Germany); ethyl propanoate (≥99%), hexan-1-ol (99%), phenylacetaldehyde (95%), 2-phenylethyl acetate (98%) were from Thermo Fisher Scientific (Dreieich, Germany); 2-methylpropan-1-ol (≥99%) was from TCI (Eschborn, Germany). 3-Methylbut-2-ene-1-thiol was synthesized according to a previously published procedure.^26^ The absence of odor-active impurities in the reference odorants was confirmed by GC–O.^27^

### Miscellaneous Chemicals

Citric acid monohydrate, glycerol, potassium hydroxide, and (2*R*,3*R*)-tartaric acid were purchased from Merck. Ethanol (99.9%) was obtained from Honeywell (Seelze, Germany).

### Aroma Models

For the beer aroma base model, glycerol (1.3 g) and citric acid (150 mg) were added to a 1 L volumetric flask and dissolved in a minimum amount of deionized water (∼10 mL).^28^ Distinct volumes (40 μL–1.3 mL) of individual ethanolic stock solutions prepared from the reference odorants or aqueous dilutions thereof were added to achieve final concentrations in agreement with the data compiled in Table 1. Further ethanol was added to achieve a final concentration of 5% ALC/VOL. The solution was first made up to ∼990 mL with deionized and carbonated water, and the pH was adjusted to 4.5 using aqueous potassium hydroxide (2 mol/L) before the solution was finally made up to 1 L. For the wine aroma base model, glycerol (7.5 g) and tartaric acid (1.3 g) were added to a 1 L volumetric flask and dissolved in a minimum amount of deionized water (∼10 mL).^28^ Distinct volumes (30 μL–3 mL) of individual ethanolic stock solutions prepared from the reference odorants were added to achieve final concentrations in agreement with the data compiled in Table 2. Further ethanol was added to achieve a final concentration of 12.9% ALC/VOL. The solution was first made up to ∼990 mL with deionized water, and the pH was adjusted to 3.4 using aqueous potassium hydroxide (2 mol/L) before the solution was finally made up to 1 L.

**Table 1:** Mean Concentrations of Beer Odorants Based on Published Data and the Corresponding OAVs

compound^a^	concentration^b^ (μg/L)	OTC^c^ (μg/kg)	OAV^d^	data set size^e^
ethyl acetate	23700	5^f^	4700	81
3-methylbutyl acetate	2070	7.2^g^	290	96
ethyl hexanoate	239	1.2^g^	200	74
2-phenylethan-1-ol	25700	140^g^	180	92
3-methylbutan-1-ol	30000	220^g^	140	73
acetaldehyde	1800	16^g^	110	10
ethyl 3-methylbutanoate	2.41	0.023^g^	100	22
dimethyl sulfide	31.0	0.30^g^	100	3
ethyl butanoate	70.6	0.76^g^	93	69
3-methylbutanal	35.0	0.50^g^	70	52
ethyl octanoate	581	8.7^g^	67	68
2-methylpropanal	28.4	0.49^g^	58	42
acetic acid	311000	5600^g^	55	3
ethyl 2-methylpropanoate	3.37	0.089^g^	38	7
octanoic acid	5930	190^g^	31	56
butane-2,3-dione	16.6	1.0^g^	17	53
phenylacetic acid	821	68^g^	12	4
3-(methylsulfanyl)propan-1-ol	421	36^g^	12	6
ethyl 2-methylbutanoate	1.30	0.13^g^	10	19
3-(methylsulfanyl)propanal	3.94	0.43^g^	9.2	24
2-methylbutan-1-ol	10300	1200^g^	8.6	64
ethyl propanoate	85.8	10^f^	8.6	19
3-methylbut-2-ene-1-thiol	0.00645	0.00076^g^	8.5	2
2-methylbutanal	8.22	1.5^g^	5.5	48
decanoic acid	2360	500^f^	4.7	41
2-methylpropyl acetate	205	66^f^	3.1	40
phenylacetaldehyde	13.8	5.2^g^	2.7	30
2-phenylethyl acetate	788	360^g^	2.2	59
1,1-diethoxyethane	50	25^g^	2.0	1
ethyl decanoate	84.8	122^f^	<1	57
butanoic acid	1380	2400^g^	<1	16
2-methylpropan-1-ol	9600	19000^g^	<1	78
3-methylbutanoic acid	245	490^g^	<1	15
hexanoic acid	1780	4800^g^	<1	55
octan-1-ol	35.2	110^f^	<1	5
2-methylbutanoic acid	561	3100^g^	<1	3
hexan-1-ol	35.1	590^g^	<1	14
benzaldehyde	8.37	150^g^	<1	50
ethyl dodecanoate	35.0	3500^f^	<1	4
2-methylpropanoic acid	448	60000^g^	<1	4
ethyl 2-phenylacetate	0.66	155.55^f^	<1	1
butan-1-ol	1.54	1900^g^	<1	9

a Compounds in order of decreasing OAVs.

b Arithmetic mean of individual values resulting from the literature survey; individual values are available in the Supporting Information, Table S3.

c Orthonasal odor threshold concentration in water.

d Odor activity value; approximated as ratio of the mean concentration in beer to the orthonasal odor threshold concentration in water.

e Number of concentration values used to calculate the mean.

f Data from literature.^44−49^

g Data taken from the Leibniz-LSB@TUM Odorant Database.^39^

**Table 2:** Mean Concentrations of Wine Odorants Based on Published Data and the Corresponding OAVs

compound^a^	concentration^b^ (μg/L)	OTC^c^ (μg/kg)	OAV^d^	data set size^e^
ethyl acetate	69100	5^f^	14000	464
acetaldehyde	49100	16^g^	3100	144
butane-2,3-dione	1400	1.0^g^	1400	85
ethyl hexanoate	1570	1.2^g^	1300	658
ethyl 3-methylbutanoate	27.5	0.023^g^	1200	320
ethyl 2-methylpropanoate	93.5	0.089^g^	1100	264
3-methylbutan-1-ol	172000	220^g^	780	555
3-methylbutyl acetate	3650	7.2^g^	510	719
ethyl butanoate	374	0.76^g^	490	596
ethyl 2-methylbutanoate	42.7	0.13^g^	330	270
ethyl octanoate	2460	8.7^g^	280	687
3-methylbutanal	119	0.50^g^	240	104
2-phenylethan-1-ol	28700	140^g^	200	684
2-methylpropanal	36.5	0.49^g^	74	112
2-methylbutan-1-ol	70100	1200^g^	58	128
dimethyl sulfide	14.1	0.30^g^	47	82
acetic acid	219000	5600^g^	39	229
3-(methylsulfanyl)propan-1-ol	1360	36^g^	38	311
3-(methylsulfanyl)propanal	14.6	0.43^g^	34	122
ethyl propanoate	295	10^f^	30	150
octanoic acid	5580	190^g^	29	582
3-hydroxybutan-2-one	16600	590^g^	28	164
2-methylbutanal	40.2	1.5^g^	27	31
phenylacetic acid	452	68^g^	6.6	48
ethyl decanoate	741	122^f^	6.1	595
decanoic acid	2460	500^f^	4.9	489
hexan-1-ol	2710	590^g^	4.6	657
phenylacetaldehyde	21.5	5.2^g^	4.1	185
2-phenylethyl acetate	682	360^g^	1.9	607
2-methylpropan-1-ol	33000	19000^g^	1.7	486
3-methylbutanoic acid	814	490^g^	1.7	264
2-methylpropyl acetate	101	66^f^	1.5	241
hexanoic acid	4060	4800^g^	<1	533
benzaldehyde	108	150^g^	<1	323
butan-1-ol	1120	1900^g^	<1	259
butanoic acid	1180	2400^g^	<1	241
octan-1-ol	44.7	110^f^	<1	169
ethyl 2-phenylacetate	53.8	155.55^f^	<1	208
2-methylbutanoic acid	545	3100^g^	<1	64
ethyl dodecanoate	269	3500^f^	<1	247
propanoic acid	1490	20000^g^	<1	64
2-methylpropanoic acid	2180	60000^g^	<1	235

a Compounds in order of decreasing OAVs.

b Arithmetic mean of individual values resulting from the literature survey; individual values are available in the Supporting Information, Table S6.

c Orthonasal odor threshold concentration in water.

d Odor activity value; approximated as ratio of the mean concentration in wine to the orthonasal odor threshold concentration in water.

e Number of concentration values used to calculate the mean.

f Data from literature.^44−49^

g Data taken from the Leibniz-LSB@TUM Odorant Database.^39^

Aroma models used in the concentration leveling tests were prepared on the basis of either the beer aroma base model or the wine aroma base model; however, the concentrations of defined groups of odorants were adjusted to the levels present in the aroma base model of the other beverage.

### Sensory Evaluations

All sensory tests were carried out in separate booths of a room exclusively dedicated to sensory evaluations. The room temperature was 22 ± 2 °C. Samples (10 mL) were provided in cylindrical polytetrafluoroethylene vessels (5.7 cm height, 3.5 cm inner diameter, 50 mL nominal volume) with lids (Bohlender; Grünsfeld, Germany). The sample temperature was 10 °C. Samples were evaluated orthonasally.

Three initial sensory sessions were dedicated to training and panel member selection. In session 1, 15 assessors recruited from the sensory panel of the Leibniz-LSB@TUM were asked to familiarize themselves with the aroma characteristics of beer and wine using nine test samples. Samples 1–4 consisted of a commercial beer sample and three commercial wine samples. The beer was made with a single hop addition at the beginning of the boil and thus did not show a hoppy aroma character. The wines did not show distinct varietal characters and were not barrel-aged. The beer was additionally presented after defoaming by filtration through a paper filter (sample 5). Samples 6–9 were volatile isolates of the four commercial beverages obtained by automated solvent-assisted flavor evaporation (aSAFE)^29^ at 40 °C using valve open/closed time combinations of 0.2 s/30 s (beer) and 0.2 s/60 s (wine). In training session 2, the ability of the assessors to correctly assign the samples used in session 1 to beer or wine was tested. For this purpose, the samples were presented in a random order and tested blindly. Likewise, in training session 3, the ability of the assessors to correctly assign the aroma base models of beer and wine was tested. Nine assessors with 100% correct answers in training sessions 2 and 3 were finally selected to perform the concentration leveling tests. This expert panel consisted of four males and five females aged 29–54. From time to time, they were retested according to training session 3 to ensure their expert status.

In the concentration leveling tests, the assessors were provided with the test sample, as well as with the aroma base models as references. They were asked to evaluate the test sample for its similarity with beer and wine and accordingly mark a position on a 20 cm ruler, of which the left margin was defined as 100% beer-like and 0% wine-like, represented by the beer aroma base model, whereas the right margin was defined as 100% wine-like and 0% beer-like and was represented by the wine aroma base model. The information and evaluation sheets given to the assessors are available in the Supporting Information. The marks on the ruler were finally converted to percentages of beer-like and wine-like depending on the exact position of the mark between the extremes. From the results of the individual assessors, the panel result was calculated as an arithmetic mean.

## Results and Discussion

### Meta-Analysis

The literature survey initially resulted in a total of ∼950 and ∼6650 articles for beer and wine, respectively. After applying the sample and quality criteria filters detailed in the Materials and Methods section, the numbers substantially decreased to 32 papers containing beer odorant concentrations and 252 papers containing wine odorant concentrations (cf. Supporting Information, Tables S1 and S4). The papers covered a total of 160 beer samples and 904 wine samples (cf. Supporting Information, Tables S2 and S5). Concentration data were subsequently extracted for all odorants that were considered to contribute to the aroma of beer or wine in general, i.e., independently of special raw materials and processing variants.

Compounds *not* considered included wine odorants associated with specific grape varieties and wine odorants originating from barrel aging. For example, TDN characterizes wines made from Riesling grapes, 4-methyl-4-sulfanylpentan-2-one characterizes Sauvignon Blanc wines, and odor-active amounts of *cis*-whisky lactone are associated with barrel aging.^16,21,30^ Likewise, beer odorants simply transferred from hops were excluded from the meta-analysis because due to evaporation they do not appear in beers brewed with a single hop dosage at the beginning of wort boiling. For this reason, e.g., linalool and geraniol were not considered. Hop-derived 3-methylbut-2-ene-1-thiol, however, was included in the meta-analysis, as it is formed in beer from nonvolatile hop bitter constituents. Among the compounds finally classified as basic beer and wine odorants were, as expected, particularly compounds formed during fermentation.^31−37^ For example, compounds 2-phenylethan-1-ol, 3-methylbutan-1-ol, and 2-methylbutan-1-ol are typical fermentation byproducts.

The total numbers of compounds classified as basic beer and wine odorants were 42 for beer and 42 for wine. The vast majority, namely, 40 odorants, were common to both beverages. The concentration data extracted from the literature for the compounds consisted of 14 845 individual concentration values. These are compiled in the Supporting Information, Tables S3 and S6. For each odorant, a mean concentration value was then calculated in beer and wine, respectively (Tables 1 and 2). The overall range of the mean odorant concentrations in beer was 6.45 ng/L (3-methylbut-2-ene-1-thiol) to 311 mg/L (acetic acid), whereas in wine, the values ranged from 14.1 μg/L (dimethyl sulfide) to 219 mg/L (acetic acid).

To assess the aroma relevance of the individual compounds, odor activity values (OAVs) were calculated from the concentration values (cf. Tables 1 and 2, fourth column). This approach resulted in 13 beer odorants and 10 wine odorants whose mean concentrations were below the OTCs, i.e., showed OAVs <1. These compounds were thus not further considered, leaving 29 beer odorants and 32 wine odorants that were considered essential for the aroma base models. Among them, 27 odorants were common to beer and wine. The compound with the highest mean OAV was ethyl acetate in both beer (OAV 4700) and wine (OAV 14 000). The mean OAVs of nine of these 27 odorants were quite similar in beer and wine, i.e., they did not differ by more than a factor of 2. Only two compounds, dimethyl sulfide and 2-methylpropyl acetate, showed slightly higher OAVs in beer, whereas 16 odorants showed higher OAVs in wine. Particularly high differences (factor wine/beer >10) were obtained for butane-2,3-dione, ethyl 2-methylbutanoate, ethyl 2-methylpropanoate, acetaldehyde, and ethyl 3-methylbutanoate.

Of the 32 wine odorants with mean OAVs >1 in our study, 22 were also included in the aroma base of wine suggested by Ferreira.^22^ Among the five additional compounds included in Ferreira’s set were ethanol, which we treated as a matrix component rather than an odorant (cf. section below) and the four carboxylic acids hexanoic, butanoic, 2-methylbutanoic, and 2-methylpropanoic acid, which were part of our initial compound selection but were finally discarded because their mean OAVs were <1. On the other hand, our wine base compound selection included 10 additional odorants not considered by Ferreira, namely 3-methylbutanal (OAV 240), 2-methylpropanal (OAV 74), 2-methylbutan-1-ol (OAV 58), dimethyl sulfide (OAV 47), 3-(methylsulfanyl)propanal (OAV 34), ethyl propanoate (OAV 30), 3-hydroxybutan-2-one (OAV 28), 2-methylbutanal (OAV 27), phenylacetic acid (OAV 6.6), and phenylacetaldehyde (OAV 4.1). Five of these additional compounds were aldehydes formed by amino acid degradation.^36,38^

### Beer and Wine Aroma Base Models

The outcome of the literature survey and the subsequent meta-analysis, i.e., the 29 beer and 32 wine odorants showing OAVs >1 and their corresponding mean concentrations (cf. Tables 1 and 2), formed the data basis for our aroma base models. The models were hydroalcoholic solutions of the odorants and additionally included major acids, glycerol, pH adjustment, and, in the case of the beer model, carbonation (Supporting Information, Table S7). An expert panel of nine trained assessors was repeatedly able to correctly assign the aroma base models in blind tests to beer and wine, respectively. Thus, the aroma base models for beer and wine reflected the basic olfactory difference between the two. This was the prerequisite for the subsequent concentration leveling tests.

### Concentration Leveling Tests

To get an idea of which compounds contribute to the aroma difference between the beer and wine aroma base models, the concentration levels of selected odorants in one model were adjusted to the concentration levels in the other model, and the effect on the overall odor was evaluated in a sensory test. For this purpose, the odorants were classified into seven groups according to their predominant odor character,^39^ namely “buttery”, “fruity”, “malty”, “honey”, “sweaty”, “sulfury”, or “miscellaneous” (Table 3). Based on the OAV sums, the biggest difference between the beer and wine aroma models was in the buttery group, followed by the fruity, malty, and miscellaneous groups. No substantial difference between beer and wine was found in the OAV sums of the honey, sweaty, and sulfury groups.

**Table 3:** OAV Sums of Odorant Groups in the Aroma Base Models of Beer and Wine

		OAV sum^b^		
group^a^	odorant	beer	wine	OAV sum ratio wine/beer^c^
“buttery”	butane-2,3-dione	17	1400	86
	3-hydroxybutan-2-one			

“fruity”	1,1-diethoxyethane	810	5200	6.4
	ethyl butanoate			
	ethyl decanoate			
	ethyl hexanoate			
	ethyl 2-methylbutanoate			
	ethyl 3-methylbutanoate			
	ethyl 2-methylpropanoate			
	ethyl octanoate			
	ethyl propanoate			
	3-methylbutyl acetate			
	2-methylpropyl acetate			

“malty”	2-methylbutanal	280	1200	4.2
	3-methylbutanal			
	2-methylbutan-1-ol			
	3-methylbutan-1-ol			
	2-methylpropanal			
	2-methylpropan-1-ol			

“miscellaneous”	acetaldehyde	4900	17000	3.5
	acetic acid			
	ethyl acetate			
	hexan-1-ol			

“honey”	phenylacetaldehyde	200	220	1.1
	phenylacetic acid			
	2-phenylethan-1-ol			
	2-phenylethyl acetate			

“sweaty”	decanoic acid	36	36	1.0
	3-methylbutanoic acid			
	octanoic acid			

“sulfury”	dimethyl sulfide	130	120	0.89
	3-methylbut-2-ene-1-thiol			
	3-(methylsulfanyl)propanal			
	3-(methylsulfanyl)propan-1-ol			

a Odorants were grouped into “buttery”, “fruity”, “malty”, “honey”, “sweaty”, “sulfury”, or “miscellaneous” according to their predominant odor qualities.^39^

b Sum of the OAVs of the individual odorants within the group.

c For each group, a ratio was calculated by dividing the OAV sum in wine by the OAV sum in beer.

**Figure 1:**
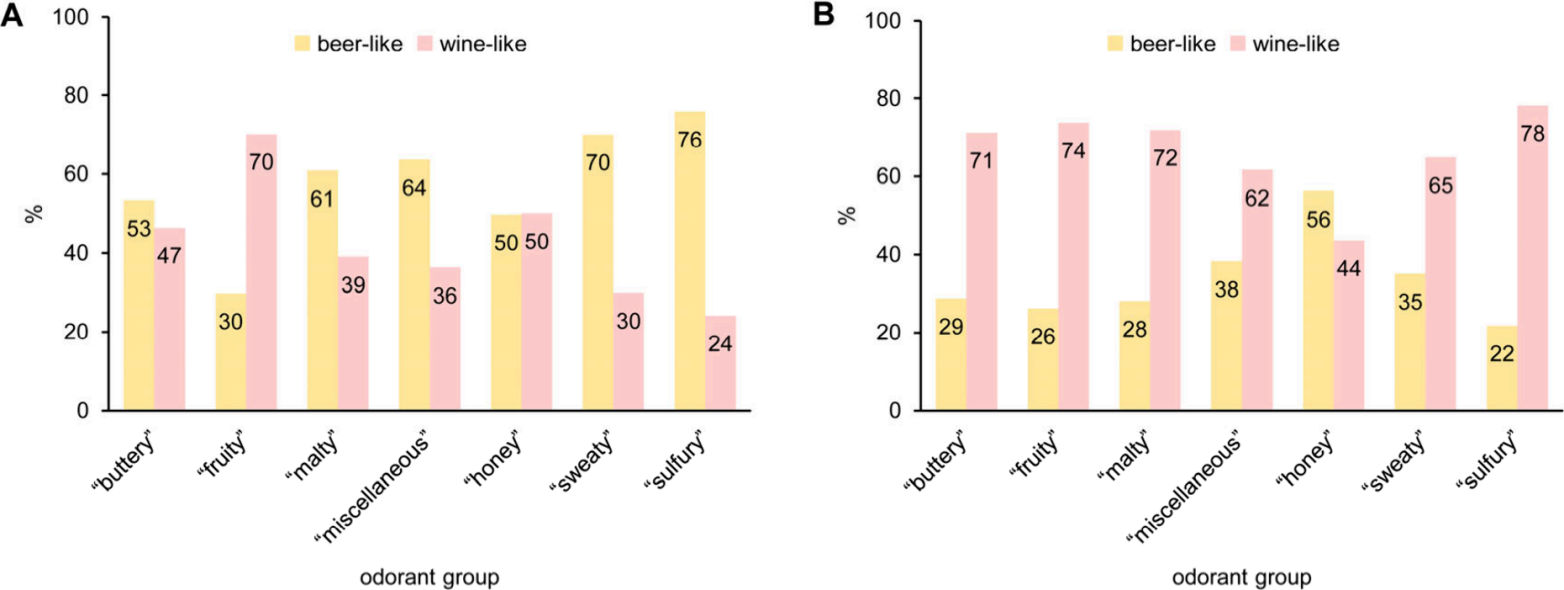
Results of the concentration leveling tests based on the beer (A) and wine (B) aroma base models. Assessors rated the odor similarity of test samples to the beer and wine aroma base models.

The results of the concentration leveling tests are summarized in Figure 1. Figure 1A depicts the results of the sensory tests based on the beer aroma base model. This means the test samples equaled the beer aroma base model in all matrix compound concentrations (including the ethanol concentration) and all odorant concentrations except those assigned to the odorant group given on the *x*-axis. As detailed in Figure 1A, when the concentrations of the sulfury, sweaty, miscellaneous, or malty odorants were adjusted to the levels in the wine aroma base model, the panel still evaluated the overall odor as more beer-like, with percentages of 61–76%. This was considered a clear result, given that even an experiment with the full beer aroma base model as a test sample, i.e., the test sample was identical to the reference sample defining 100% beer-like, did not give a result of 100% but only of 88% (data not shown). No clear assignment to beer or wine was possible after the buttery or honey-like odorants were adjusted to the wine aroma base levels. However, the biggest effect was observed when the concentrations of the fruity odorants, which were clearly more odor-active in the wine model (cf. Table 3), were adjusted. In this case, the test sample was rated more wine-like (70%) than beer-like (30%), suggesting higher ester levels in wine as a crucial parameter for the aroma difference to beer.

The overall olfactory impression of the wine aroma base model (Figure 1B) was even more stable than that of the beer aroma base model. Six of the seven leveling tests still resulted in a clear assignment to wine, even including the test in which the fruity compounds were adjusted to the beer aroma base levels. The corresponding percentages obtained for wine-like ranged from 62 to 78%; the full wine aroma base model as a test sample resulted in 83% wine-like (data not shown). The only leveling test using the wine aroma base model that resulted in an inversion of the sensory evaluation was the one in which the honey-like odorants were adjusted to the levels in the beer aroma base model. In this case, the test sample was rated slightly more beer-like than wine-like. This result, on the one hand, was surprising because the concentration differences between the beer and wine aroma base models in the four honey-like smelling odorants were only minor, but, on the other hand, was in line with the result obtained with the beer aroma base model depicted in Figure 1A.

To answer the question of to what extent the matrix composition contributed to the assignment of a test sample to beer or wine, two further sensory experiments were conducted. Test sample 1 combined the matrix composition of the wine aroma base model with all the odorant concentrations in the beer aroma base model, and test sample 2 combined the matrix composition of the beer aroma base model with all the odorant concentrations in the wine aroma base model. The test sample with the beer aroma base odorants in the wine matrix was evaluated as more beer-like (65%), and the test sample with the wine aroma base odorants in the beer matrix was evaluated as more wine-like (66%). Thus, percentages were somewhat lower than those obtained for the models with the beer aroma base odorants in the beer matrix (88%) and the wine aroma base odorants in the wine matrix (83%). This indicated an influence of the matrix. However, in both cases, the odorant composition and not the matrix composition determined whether the model was evaluated as more beer-like or more wine-like. In other words, the odorant composition dominated over the matrix composition. The influence of the matrix composition has been studied for beer^40^ and wine^41−43^ before, however, only separately and never in combination with a simultaneous assessment of the beer- and wine-like aroma character.

In summary, this study provided an extensive literature survey of basic odor-active compounds in beer and wine and their concentration ranges. The majority of these basic odorants originate in the fermentation step and are common to both beer and wine; however, substantial differences exist between beer and wine in the concentrations of some odorants. The sensory experiments revealed that beer and wine can be olfactorily differentiated on the basis of the basic odorants and that particularly higher ester concentrations in wine are a crucial discriminating parameter, whereas the impact of the matrix composition, including the different alcohol content, was only minor. In real life, however, further factors not investigated in this study may additionally contribute to the different perceptions of beer and wine aroma. For example, the drinking temperature of beer is normally lower than the drinking temperature of wine, whereas in our study an intermediate temperature of 10 °C was used for all sensory tests. Furthermore, the effect of beer foam on the release of odorants was also not considered, as no foaming agent was present in our beer model. Nevertheless, the finding that the odorant composition dominated over the matrix composition might be used in the beverage industry to develop novel and innovative low-alcohol beverages and to meet changing consumer preferences.

The concentration leveling tests used in our study proved to be a promising tool to clarify the molecular background of aroma differences between two samples in general and are therefore predestined for more applications in the future. These may include the comparison of fresh and stored samples, differently processed samples, samples of different varieties, and samples with and without off-flavor.

## Supporting Information Available

References used for data extraction, sample characteristics, and extracted odorant concentrations, ethanol concentrations, and pH data; matrix compositions of the aroma base models; additional information on sensory analyses (PDF)

## Abbreviations

aSAFE: automated solvent-assisted flavor evaporation
FID: flame ionization detector
GC: gas chromatography
LC: liquid chromatography
MS: mass spectrometry
O: olfactometry
OAV: odor activity value
OTC: odor threshold concentration
TDN: 1,1,6-trimethyl-1,2-dihydronaphthalene
**Nomenclature**
citric acid monohydrate: 2-hydroxypropane-1,2,3-tricarboxylic acid hydrate
dimethyl sulfide: (methylsulfanyl)methane
glycerol: propane-1,2,3-triol
(2*R*,3*R*): tartaric acid,
(2*R*,3*R*)-2: 3-dihydroxybutanedioic
*cis*-whisky lactone: (4*R*,5*R*)/(4*S*,5*S*)-5-butyl-4-methyloxolan-2-one
