# Supplementary material for: Molecular Insights into the Aroma Difference between Beer and Wine: A Meta-Analysis-Based Sensory Study Using Concentration Leveling Tests
Source: J Agric Food Chem. 2024 Sep 30;72(40):22250–7. doi: 10.1021/acs.jafc.4c06838 (PMC11468751; doi:10.1021/acs.jafc.4c06838)
Supplement: Supplementary file 1 — jf4c06838_si_001.pdf [file jf4c06838_si_001.pdf]

Supporting Information

Molecular Insights into the Aroma Difference between Beer and Wine: a Meta-Analysis-Based Sensory Study Using Concentration Leveling Tests

Xingjie Wang,[1,2] Stephanie Frank,[2,*] and Martin Steinhaus[2,1,*]

[1] Technical University of Munich, TUM School of Natural Sciences, Department of Chemistry, Lichtenbergstraße 4, 85748 Garching, Germany
[2] Leibniz Institute for Food Systems Biology at the Technical University of Munich (Leibniz-LSB@TUM), Lise-Meitner-Straße 34, 85354 Freising, Germany

*E-mail: s.frank.leibniz-lsb@tum.de, m.steinhaus.leibniz-lsb@tum.de

Overview

Additional Tables

Table S1. References on Beer Odorants Used for Data Extraction

Table S2. Beer Samples Used for Data Extraction

Table S3. Individual Odorant Concentrations, Ethanol Concentrations, and pH in the Selected Beer Samples (cf. Table S2) as Extracted from the Literature (cf. Table S1)

Table S4. References on Wine Odorants Used for Data Extraction

Table S5. Wine Samples Used for Data Extraction

Table S6. Individual Odorant Concentrations, Ethanol Concentrations, and pH in the Selected Wine Samples (cf. Table S5) as Extracted from the Literature (cf. Table S4)

Table S7. Matrix Compositions of the Beer and Wine Aroma Base Models

Additional Information on Sensory Analyses

Instructions Provided to the Assessors

Form to Be Filled by the Assessors

Table S1. References on Beer Odorants Used for Data Extraction

no.	reference	no.	reference
1	*J. Agric. Food Chem.* **2005**, *53*, 7544–7551.	17	*Food Res. Int.* **2019**, *123*, 75–87.
2	*J. Agric. Food Chem.* **2021**, *69*, 8190–8199.	18	*Food Res. Int.* **2019**, *126*, 108680.
3	*Foods* **2020**, *9*, 255.	19	*Food Anal. Method.* **2016**, *9*, 3230–3241.
4	*Brew. Sci.* **2020**, *73*, 26–40.	20	*J. Agric. Food Chem.* **2016**, *64*, 8035–8044.
5	*Food Anal. Method.* **2019**, *12*, 2293–2305.	21	*Brew. Sci.* **2016**, *69*, 73–84.
6	*Eur. Food Res. Technol.* **2021**, *247*, 427–437.	22	*LWT–Food Sci. Technol.* **2016**, *66*, 390–397.
7	*J. Am. Soc. Brew. Chem.* **2014**, *72*, 154–161.	23	*Talanta* **2013**, *117*, 523–531.
8	*J. Agric. Food Chem.* **2010**, *58*, 3107–3115.	24	*Int. J. Food Microbiol.* **2013**, *161*, 76–83.
9	*Z. Lebensm.-Unters. Forsch.* **1991**, *193*, 558–565.	25	*J. Am. Soc. Brew. Chem.* **2011**, *69*, 50–56.
10	*Food Anal. Method.* **2012**, *5*, 1386–1397.	26	*Food Chem.* **2008**, *107*, 242–249.
11	*J. Am. Soc. Brew. Chem.* **2019**, *77*, 113–118.	27	*Food Chem.* **2009**, *114*, 1206–1215.
12	*J. Biosci. Bioeng.* **2018**, *126*, 330–338.	28	*J. Chromatogr. A* **2008**, *1190*, 342–349.
13	*J. Inst. Brew.* **2018**, *124*, 244–253.	29	*J. Am. Soc. Brew. Chem.* **2006**, *64*, 52–60.
14	*J. Agric. Food Chem.* **2006**, *54*, 8855–8861.	30	*J. Am. Soc. Brew. Chem.* **2002**, *60*, 88–96.
15	*LWT–Food Sci. Technol.* **2021**, *148*, 111755.	31	*J. Agric. Food Chem.* **2020**, *68*, 8602–8612.
16	*Beverages* **2021**, *7*, 4.	32	*J. Agric. Food Chem.* **2016**, *64*, 646–652.

Table S2. Beer Samples Used for Data Extraction

no.	beer sample	no.	beer sample
1	Bavarian Pilsner beer	81	Spanish regular Lager beer 5
2	German beer	82	Spanish regular Lager beer 6
3	Lager beer	83	Spanish regular Lager beer 7
4	New England IPA beer 1	84	Spanish regular Lager beer 8
5	New England IPA beer 2	85	Spanish regular Lager beer 9
6	Spanish Lager beer 1	86	Spanish regular Lager beer 10
7	Spanish Lager beer 2	87	Portuguese regular Lager beer
8	Spanish Lager beer 3	88	German regular Lager beer 1
9	Spanish Lager beer 4	89	Dutch regular Lager beer
10	Spanish Lager beer 5	90	German regular Lager beer 2
11	Spanish Lager beer 6	91	Belgian regular Lager beer
12	Spanish Lager beer 7	92	German regular Lager beer 3
13	Spanish Lager beer 8	93	Pilsner beer (fresh)
14	Spanish Lager beer 9	94	Pilsner beer (aged)
15	Spanish Lager beer 10	95	Lager beer 1 (fresh)
16	Spanish Lager beer 11	96	Lager beer 2 (fresh)
17	Czech Lager beer 1	97	Spanish Lager beer 1
18	Czech Lager beer 2	98	Spanish Lager beer 2
19	Czech Lager beer 3	99	Spanish Lager beer 3
20	Czech Lager beer 4	100	Spanish Lager beer 4
21	Czech Lager beer 5	101	Spanish Lager beer 5
22	Czech Lager beer 6	102	Spanish Lager beer 6
23	Czech Lager beer 7	103	Spanish Lager beer 7
24	Czech Lager beer 8	104	Spanish Lager beer 8
25	Czech Lager beer 9	105	Spanish Lager beer 9
26	Czech Lager beer 10	106	Spanish Lager beer 10
27	Czech Lager beer 11	107	Spanish Lager beer 11
28	Czech Lager beer 12	108	Spanish Lager beer 12
29	Chinese Tsingtao beer 1	109	Czech Lager beer 1
30	Chinese Tsingtao beer 2	110	Czech Lager beer 2
31	Chinese Tsingtao beer 3	111	Czech Lager beer 3
32	Chinese Tsingtao beer 4	112	Czech Lager beer 4
33	Japanese Pilsner beer 1	113	Czech Lager beer 5
34	Japanese Pilsner beer 2	114	Czech Lager beer 6
35	Japanese Pilsner beer 3	115	Czech Lager beer 7
36	Japanese Pilsner beer 4	116	Czech Lager beer 8
37	Japanese dark Larger beer	117	Czech Lager beer 9
38	Belgian Lager beer (fresh)	118	Czech Lager beer 10
39	Belgian Lager beer (aged)	119	Czech Lager beer 11
40	Bavarian pale Lager beer	120	Czech Lager beer 12
41	Bavarian dark Lager beer	121	Lager beer 1
42	Spanish Pilsner beer (fresh)	122	Lager beer 2
43	Spanish Pilsner beer (aged 1)	123	Lager beer 3
44	Spanish Pilsner beer (aged 2)	124	Lager beer 4
45	Spanish Pilsner beer (aged 3)	125	Lager beer 5
46	Spanish Pilsner beer (aged 4)	126	Lager beer 6
47	Spanish Pilsner beer (aged 5)	127	Lager beer 7
48	Spanish Pilsner beer (aged 6)	128	Lager beer 8
49	Spanish Pilsner beer (aged 7)	129	Lager beer 9
50	Spanish Pilsner beer (aged 8)	130	Lager beer 10
51	Spanish Pilsner beer (aged 9)	131	Lager beer
52	Spanish Pilsner beer (aged 10)	132	Lager beer (fresh)
53	USA beer 1	133	Lager beer (aged)
54	USA beer 2	134	Pilsner beer (fresh)
55	USA beer 3	135	Pilsner beer (aged 1)
56	USA beer 4	136	Pilsner beer (aged 2)
57	USA beer 5	137	Belgian Lager beer 1 (fresh)
58	USA beer 6	138	Belgian Lager beer 2 (fresh)
59	USA beer 7	139	Belgian Lager beer 3 (fresh)
60	USA beer 8	140	Pilsner beer
61	USA beer 9	141	bottom-fermented beer 1
62	Japanese Pilsner beer	142	bottom-fermented beer 2
63	Czech Lager beer 1	143	Belgian Lager beer 1
64	Czech Lager beer 2	144	Belgian Lager beer 2
65	Czech Lager beer 3	145	Dutch Lager beer 1
66	Czech Lager beer 4	146	Dutch Lager beer 2
67	Czech Lager beer 5	147	German Lager beer
68	Czech Lager beer 6	148	Danish Lager beer
69	Japanese unhopped beer	149	French Lager beer
70	Japanese hopped beer 1	150	New Zealand Lager beer 1
71	Japanese hopped beer 2	151	New Zealand Lager beer 2
72	Japanese hopped beer 3	152	New Zealand Lager beer 3
73	Czech bottom-fermented beer	153	USA Lager beer 1
74	English beer	154	USA Lager beer 2
75	Brazilian Lager beer	155	Canadian Lager beer
76	Lager beer	156	Mexican Lager beer
77	Spanish regular Lager beer 1	157	Chinese Lager beer
78	Spanish regular Lager beer 2	158	Japanese Lager beer
79	Spanish regular Lager beer 3	159	bottom-fermented beer
80	Spanish regular Lager beer 4	160	Pilsner beer

Table S3. Individual Odorant Concentrations, Ethanol Concentrations, and pH in the Selected Beer Samples (cf. Table S2) as Extracted from the Literature (cf. Table S1)

		reference no.	1	2	3	4	4	5	5	5	5	5	5	5	5	5	5
		beer sample no.	1	2	3	4	5	6	7	8	9	10	11	12	13	14	15
matrix		mean															
ethanol (%, ALC/VOL)		5.0		5.08				6.5	3.5	5.4	4.8	6.4	4.7	5.0	6.4	5.5	5.2
pH		4.5															
odorant	OTC (µg/kg) in water	mean concentration (µg/L)															
ethyl acetate	5	23700				49900	47900	32040	22350	27350	39930	24850	38880	31200	35160	28380	62400
3-methylbutyl acetate	7.2	2070		1.8#	2610	1480	1470	2840	1680	2770	4100	2400	2410	4670	4160	2770	7250
ethyl hexanoate	1.2	239	205	8.4#	307.14	566	588	250	100	160	1050	210	230	200	1060	300	600
2-phenylethan-1-ol	140	25700	15100	14000#		35800	35600	37540	14430	20830	27850	50300	32650	36370	45030	34550	31080
3-methylbutan-1-ol	220	30000	49600	1200#	51320	113000	117000	21720	9360	16750	21270	26680	10860	15140	32230	19700	18410
acetaldehyde	16	1800	5100														
ethyl 3-methylbutanoate	0.023	2.41			4.08												
dimethyl sulfide	0.30	31.0	59														
ethyl butanoate	0.76	70.6	198		107.85	135	153	40	40	50	90	50	60	40	80	40	100
3-methylbutanal	0.50	35.0	4.0														
ethyl octanoate	8.7	581	160		139.08	246	266	50	30	40	130	60	20	70	260	50	110
2-methylpropanal	0.49	28.4															
acetic acid	5600	311000		630000#													
ethyl 2-methylpropanoate	0.089	3.37	3.2														
octanoic acid	190	5930			199.88			2140	1990	1670	12710	2280	3860	2880	18720	3500	21360
butane-2,3-dione	1.0	16.6															
phenylacetic acid	68	821	257	640#													
3-(methylsulfanyl)propan-1-ol	36	421	991	610#													
ethyl 2-methylbutanoate	0.13	1.30		4.1#													
3-(methylsulfanyl)propanal	0.43	3.94		2.7#													
2-methylbutan-1-ol	1200	10300	14400	370#		10400	9200	9580	5460	12340	15490	19160	5670	12010	23570	9670	13120
ethyl propanoate	10	85.8															
3-methylbut-2-ene-1-thiol	0.00076	0.00645	0.01														
2-methylbutanal	1.5	8.22															
decanoic acid	500	2360						1990	240	260	7710	270	590	310	22990	1240	9150
2-methylpropyl acetate	66	205				73	63	270	150	170	380	280	230	420	450	170	650
phenylacetaldehyde	5.2	13.8		10#													
2-phenylethyl acetate	360	788			360.93	367	375	750	390	510	910	830	930	1520	1000	720	2500
1,1-diethoxyethane	25	50	50														
ethyl decanoate	122	84.8			50.07	28	34	10	10	10	90	10		10	120	10	20
butanoic acid	2400	1380	1800	1400#													
2-methylpropan-1-ol	19000	9600		780#		18000	14600	11240	6520	12200	15440	15030	5910	11500	27190	9630	12350
3-methylbutanoic acid	490	245	855														
hexanoic acid	4800	1780						1630	1010	1110	4080	1480	1850	1250	4160	1500	7970
octan-1-ol	110	35.2			5.47												
2-methylbutanoic acid	3100	561	438														
hexan-1-ol	590	35.1			11.26												
benzaldehyde	150	8.37			7.87												
ethyl dodecanoate	3500	35.0			17.38												
2-methylpropanoic acid	60000	448		850#	6.05												
ethyl 2-phenylacetate	155.55	0.66															
butan-1-ol	1900	1.54															

#value in µg/kg was converted to µg/L with a factor of 1.

*value was considered false high and excluded from mean calculation.

		reference no.	5	5	5	5	5	5	5	5	5	5	5	5	5	6	6
		beer sample no.	16	17	18	19	20	21	22	23	24	25	26	27	28	29	30
matrix		mean															
ethanol (%, ALC/VOL)		5.0	5.5	4.0	4.0	4.0	4.4	5.0	5.1	4.4	5.0	3.8	4.7	7.5	5.2		
pH		4.5															
odorant	OTC (µg/kg) in water	mean concentration (µg/L)															
ethyl acetate	5	23700	34870	27130	30650	55170	24440	56830	49800	24930	30270	50190	50940	169230	114290	19.8#	32.6#
3-methylbutyl acetate	7.2	2070	4410	2330	1860	3930	1260	5020	2450	2420	3520	4170	5750	10200	7640	690#	732#
ethyl hexanoate	1.2	239	330	100	190	310	290	250	370	400	210	270	260	410	350	380#	473#
2-phenylethan-1-ol	140	25700	24260	18470	19790	12610	11000	25250	27920	39980	21910	17320	14760	25690	9670	2549#	3959#
3-methylbutan-1-ol	220	30000	15530	12630	8590	8030	6340	12250	8750	14040	16760	8300	11100	15660	10870		
acetaldehyde	16	1800															
ethyl 3-methylbutanoate	0.023	2.41															
dimethyl sulfide	0.30	31.0															
ethyl butanoate	0.76	70.6	60	40	30	60	50	60	60	40	60	70	80	150	110	19.8#	24.6#
3-methylbutanal	0.50	35.0															
ethyl octanoate	8.7	581	30	20	30	20	40	20	80	30	20	10	10	20	20	2517#	3364#
2-methylpropanal	0.49	28.4															
acetic acid	5600	311000															
ethyl 2-methylpropanoate	0.089	3.37															
octanoic acid	190	5930	5450	3610	6840	8820	17360	9290	18400	17190	4350	15700	19170	23110	18230	1338#	2345#
butane-2,3-dione	1.0	16.6															
phenylacetic acid	68	821															
3-(methylsulfanyl)propan-1-ol	36	421															23.1#
ethyl 2-methylbutanoate	0.13	1.30															
3-(methylsulfanyl)propanal	0.43	3.94															
2-methylbutan-1-ol	1200	10300	7890	6750	5900	4350	4410	4170	5960	6940	9270	4610	5350	6330	7040		
ethyl propanoate	10	85.8															
3-methylbut-2-ene-1-thiol	0.00076	0.00645															
2-methylbutanal	1.5	8.22															
decanoic acid	500	2360	950	2000	450	640	2410	360	4510	8570	310	1000	1120	1580	2060	490#	812#
2-methylpropyl acetate	66	205	330	90	100	160	90	310	190	130	180	330	560	640	640		
phenylacetaldehyde	5.2	13.8															
2-phenylethyl acetate	360	788	920	580	820	1070	390	2040	1360	2140	600	1730	1630	3670	2000	968#	1388#
1,1-diethoxyethane	25	50															
ethyl decanoate	122	84.8		40	10	20	60	20	240	50	120	110	10	40	150	376#	589#
butanoic acid	2400	1380															5.67#
2-methylpropan-1-ol	19000	9600	6310	5590	3740	2970	4940	5860	5580	10190	10480	5080	4450	6910	6980		
3-methylbutanoic acid	490	245															
hexanoic acid	4800	1780	2130	1090	760	190	310	890	2950	1260	650	190	840	650	780	76.8#	119#
octan-1-ol	110	35.2															44.1#
2-methylbutanoic acid	3100	561															
hexan-1-ol	590	35.1															
benzaldehyde	150	8.37														1.48#	5.2#
ethyl dodecanoate	3500	35.0															
2-methylpropanoic acid	60000	448															
ethyl 2-phenylacetate	155.55	0.66															
butan-1-ol	1900	1.54															

#value in µg/kg was converted to µg/L with a factor of 1.
*value was considered false high and excluded from mean calculation.

		reference no.	6	6	7	7	7	7	7	8	8	9	9	10	10	10	10
		beer sample no.	31	32	33	34	35	36	37	38	39	40	41	42	43	44	45
matrix		mean															
ethanol (%, ALC/VOL)		5.0												5.4			
pH		4.5															
odorant	OTC (µg/kg) in water	mean concentration (µg/L)															
ethyl acetate	5	23700	59.6#	94#	40600	22600	21112	16240	21924					15050	16045	15801	15586
3-methylbutyl acetate	7.2	2070	780#	861#	7820	2190	3753.6	1798.6	2267.8	356	216			1908	2087	1947	1747
ethyl hexanoate	1.2	239	522#	583#	156	123	156	126.36	106.08			149	75	115	140	109	146
2-phenylethan-1-ol	140	25700	4734#	5097#	20600	9410	21836	12154	9682			17500	9100	17130	20791	19025	11405
3-methylbutan-1-ol	220	30000			72400	44900	47060	39820	40544			49600	42900				
acetaldehyde	16	1800								588	2426						
ethyl 3-methylbutanoate	0.023	2.41			0.85	0.61	0.8925	0.935	1.513	0.70	2.56						
dimethyl sulfide	0.30	31.0		2.18#	456*	345*	560.88*	387.6*	551.76*								
ethyl butanoate	0.76	70.6	25.4#	26#	103	71	103	157.59	92.7			61	43	94	158	150	113
3-methylbutanal	0.50	35.0								8.8	25.9						
ethyl octanoate	8.7	581	4995#	5238#	881	476	255.49	273.11	211.44					126	170	150	170
2-methylpropanal	0.49	28.4								11	71						
acetic acid	5600	311000															
ethyl 2-methylpropanoate	0.089	3.37															
octanoic acid	190	5930	2540#	3396#										1326	1862	1678	1117
butane-2,3-dione	1.0	16.6								6.0	33.2						
phenylacetic acid	68	821															
3-(methylsulfanyl)propan-1-ol	36	421	34.2#	47.9#													
ethyl 2-methylbutanoate	0.13	1.30			2.0	1.9	1.42	2.62	2.52	0.41	1.12						
3-(methylsulfanyl)propanal	0.43	3.94								2.00	4.94						
2-methylbutan-1-ol	1200	10300			18100	10900	13575	13756	9593								
ethyl propanoate	10	85.8		5.3#	140	62	71.4	79.8	247.8					72	114	103	95
3-methylbut-2-ene-1-thiol	0.00076	0.00645															
2-methylbutanal	1.5	8.22								2.9	10.6						
decanoic acid	500	2360	947#	1269#													
2-methylpropyl acetate	66	205		7.17										67	106	101	104
phenylacetaldehyde	5.2	13.8			24	15	11.76	10.56	33.84	17	63						
2-phenylethyl acetate	360	788	1623#	2015#	889	173	862.33	284.48	293.37					332	438	372	292
1,1-diethoxyethane	25	50															
ethyl decanoate	122	84.8	663#	1010#										10	18	15	17
butanoic acid	2400	1380										1170	2190				
2-methylpropan-1-ol	19000	9600	27#		8990	6130	11327.4	6113.2	6382.9					13480	15228	16868	16612
3-methylbutanoic acid	490	245										244	744				
hexanoic acid	4800	1780	152#	360#										774	935	903	759
octan-1-ol	110	35.2	52.6#	60.2#													
2-methylbutanoic acid	3100	561															
hexan-1-ol	590	35.1												19	21	22	17
benzaldehyde	150	8.37	6.67#	11.5#						1.0	5.9						
ethyl dodecanoate	3500	35.0															
2-methylpropanoic acid	60000	448															
ethyl 2-phenylacetate	155.55	0.66															
butan-1-ol	1900	1.54															

#value in µg/kg was converted to µg/L with a factor of 1.
*value was considered false high and excluded from mean calculation.

		reference no.	10	10	10	10	10	10	10	11	11	11	11	11	11	11	11
		beer sample no.	46	47	48	49	50	51	52	53	54	55	56	57	58	59	60
matrix		mean															
ethanol (%, ALC/VOL)		5.0															
pH		4.5															
odorant	OTC (µg/kg) in water	mean concentration (µg/L)															
ethyl acetate	5	23700	14042	18606	16332	12438	11941	12880	14200	103.4	55.4	66.1	69.5	77.2	71.6	43.9	61.6
3-methylbutyl acetate	7.2	2070	1624	1547	1512	1469	1347	1298	1245	9.62	16.67	7.41	1.85	2.24	2.51	2.09	1.44
ethyl hexanoate	1.2	239	133	120	101	106	94	87	88	5.49	5.15	5.02	6.05	6.42	6.89	4.44	4.52
2-phenylethan-1-ol	140	25700	10162	14291	13110	15696	18271	12860	14400								
3-methylbutan-1-ol	220	30000								135.8	187.4	180.1	186.1	223.7	209.7	188.3	204.2
acetaldehyde	16	1800															
ethyl 3-methylbutanoate	0.023	2.41															
dimethyl sulfide	0.30	31.0															
ethyl butanoate	0.76	70.6	106	104	92	94	90	87	85	2.25	2.03	2.17	2.22	2.47	2.48	1.17	1.3
3-methylbutanal	0.50	35.0															
ethyl octanoate	8.7	581	150	126	94	64	56	80	74	7.7	8.26	8.2	10.35	3.57	11.46	8.34	3.96
2-methylpropanal	0.49	28.4															
acetic acid	5600	311000															
ethyl 2-methylpropanoate	0.089	3.37															
octanoic acid	190	5930	1005	1207	1151	1172	1408	1204	1321	13.89	13.38	13.7	11.36	13.93	15.69	11.85	3.36
butane-2,3-dione	1.0	16.6								1.94	2.28	3.55	4.79	1.72	2.76	1.52	0.89
phenylacetic acid	68	821															
3-(methylsulfanyl)propan-1-ol	36	421															
ethyl 2-methylbutanoate	0.13	1.30															
3-(methylsulfanyl)propanal	0.43	3.94									0.01	0.01	0.01	0.01	0.01	0.01	0.01
2-methylbutan-1-ol	1200	10300								102	81.8	89.7	93.8	64.1	103	95.9	110.9
ethyl propanoate	10	85.8	88	102	85	57	58	70	70								
3-methylbut-2-ene-1-thiol	0.00076	0.00645															
2-methylbutanal	1.5	8.22															
decanoic acid	500	2360								1.63	1.81	1.88				1.92	2.43
2-methylpropyl acetate	66	205	96	88	75	57	53	68	63								
phenylacetaldehyde	5.2	13.8															
2-phenylethyl acetate	360	788	251	320	275	259	261	276	280								
1,1-diethoxyethane	25	50															
ethyl decanoate	122	84.8	16	13	11	14	14	12	11	0.22	0.29	0.28	0.29	0.29	0.32	0.17	0.25
butanoic acid	2400	1380								3.84	4.19	4.24	4.4	5.5	5.27	3.46	4.4
2-methylpropan-1-ol	19000	9600	15816	17068	17755	13099	11937	17070	18560	33.54	24.56	31.12	48.58	63.35	53.48	52.15	71.81
3-methylbutanoic acid	490	245								1.91	1.78	1.82	1.35	1.72	1.71	2.19	2.63
hexanoic acid	4800	1780	693	688	655	818	973	790	784	7.1	9.62	9.41	9.24	10.58	10.98	7.95	8.75
octan-1-ol	110	35.2															
2-methylbutanoic acid	3100	561															
hexan-1-ol	590	35.1	17	19	18	18	19	17	17								
benzaldehyde	150	8.37															
ethyl dodecanoate	3500	35.0															
2-methylpropanoic acid	60000	448															
ethyl 2-phenylacetate	155.55	0.66															
butan-1-ol	1900	1.54								1.19	1.11	1.38	0.8	0.52	0.68	2.08	2.94

#value in µg/kg was converted to µg/L with a factor of 1.
*value was considered false high and excluded from mean calculation.

		reference no.	11	12	13	13	13	13	13	13	14	14	14	14	15	16	17
		beer sample no.	61	62	63	64	65	66	67	68	69	70	71	72	73	74	75
matrix		mean															
ethanol (%, ALC/VOL)		5.0		5.2											3.66	4.8	
pH		4.5														4.48	
odorant	OTC (µg/kg) in water	mean concentration (µg/L)															
ethyl acetate	5	23700	54.5	15300.0	17300	22010	16100	13140	19570	17130					7800	19590	
3-methylbutyl acetate	7.2	2070	2.18	1230.0	2900	2940	2680	2320	2380	2260					770	1170	1960
ethyl hexanoate	1.2	239	4.53	119.2	390	510	500	60	290	160					90	110	
2-phenylethan-1-ol	140	25700		27669.0	14040	15560	9560	10980	14250	12700					12000	35730	
3-methylbutan-1-ol	220	30000	202.5	59600.0	23230	16670	15030	24230	13150	16630					31200	63240	6290
acetaldehyde	16	1800													3000	2990	
ethyl 3-methylbutanoate	0.023	2.41		0.41							0.01	5.32	2.66	2.13			
dimethyl sulfide	0.30	31.0														31.76	
ethyl butanoate	0.76	70.6	1.15	73.1	70	90	70	50	50	50					90	70	
3-methylbutanal	0.50	35.0		4.9											3.65		
ethyl octanoate	8.7	581	6.21	159.5	160	130	140	180	230	250					250	260	15860
2-methylpropanal	0.49	28.4													1.52		
acetic acid	5600	311000															302200
ethyl 2-methylpropanoate	0.089	3.37		0.91							0.27	3.98	8.01	6.39		0.83	
octanoic acid	190	5930	10.8	1990.0	8830	10170	7820	8660	13010	14090							
butane-2,3-dione	1.0	16.6	1.19	13.0													
phenylacetic acid	68	821		2080.0													
3-(methylsulfanyl)propan-1-ol	36	421		822.0													
ethyl 2-methylbutanoate	0.13	1.30		0.21							0.02	1.67	1.83	1.20			
3-(methylsulfanyl)propanal	0.43	3.94	0.11	1.2													
2-methylbutan-1-ol	1200	10300	96.7		10530	14790	13120	16860	9560	23660							
ethyl propanoate	10	85.8		96.2												13	
3-methylbut-2-ene-1-thiol	0.00076	0.00645		0.0029													
2-methylbutanal	1.5	8.22		1.7											1.37		
decanoic acid	500	2360	2.33	370.0	3420	2970	3420	3560	4090	4030							
2-methylpropyl acetate	66	205													50	150	
phenylacetaldehyde	5.2	13.8		4.5											3.18		
2-phenylethyl acetate	360	788		382.5	350	400	300	280	440	450					10	570	
1,1-diethoxyethane	25	50															
ethyl decanoate	122	84.8	0.22		50	20	30	100	210	200					10		
butanoic acid	2400	1380	4.24	440.0													
2-methylpropan-1-ol	19000	9600	66.75	12400.0	8390	10080	8190	6010	7290	12220					3800	13760	
3-methylbutanoic acid	490	245	2.04	454.9													
hexanoic acid	4800	1780	7.91	910.0	9270	6670	5760	13320	6240	7220							
octan-1-ol	110	35.2															
2-methylbutanoic acid	3100	561															
hexan-1-ol	590	35.1															250
benzaldehyde	150	8.37		1.6											2.20		
ethyl dodecanoate	3500	35.0													10		90
2-methylpropanoic acid	60000	448															
ethyl 2-phenylacetate	155.55	0.66															
butan-1-ol	1900	1.54	3.16														

#value in µg/kg was converted to µg/L with a factor of 1.
*value was considered false high and excluded from mean calculation.

		reference no.	18	19	19	19	19	19	19	19	19	19	19	19	19	19	19
		beer sample no.	76	77	78	79	80	81	82	83	84	85	86	87	88	89	90
matrix		mean															
ethanol (%, ALC/VOL)		5.0		5.50	6.50	5.40	5.50	4.60	5.00	5.40	4.80	5.20	5.00	5.20	5.60	5.00	5.00
pH		4.5															
odorant	OTC (µg/kg) in water	mean concentration (µg/L)															
ethyl acetate	5	23700	4456.70	20380	19530	14960	21930	8820	20380	19380	13320	15270	22060	10440	9330	23170	14260
3-methylbutyl acetate	7.2	2070	3352.86	2480	1840	1580	1680	800	3990	1440	2190	1940	4400	990	1080	1770	1230
ethyl hexanoate	1.2	239	166.76														
2-phenylethan-1-ol	140	25700	16575.96	38130	40320	24410	46100	42370	47350	51180	25470	40340	35180	31820	20120	37220	35260
3-methylbutan-1-ol	220	30000	85819.94	44620	52720	43460	57320	59460	43380	53960	54120	48900	41390	42250	31610	42630	31310
acetaldehyde	16	1800															
ethyl 3-methylbutanoate	0.023	2.41	4.44														
dimethyl sulfide	0.30	31.0															
ethyl butanoate	0.76	70.6	221.41														
3-methylbutanal	0.50	35.0															
ethyl octanoate	8.7	581	157.98														
2-methylpropanal	0.49	28.4															
acetic acid	5600	311000	34.32														
ethyl 2-methylpropanoate	0.089	3.37															
octanoic acid	190	5930	4768.24														
butane-2,3-dione	1.0	16.6															
phenylacetic acid	68	821															
3-(methylsulfanyl)propan-1-ol	36	421															
ethyl 2-methylbutanoate	0.13	1.30															
3-(methylsulfanyl)propanal	0.43	3.94															
2-methylbutan-1-ol	1200	10300		13580	17040	16270	18980	23450	20000	19870	28950	5350	19160	14220	10040	15520	6540
ethyl propanoate	10	85.8															
3-methylbut-2-ene-1-thiol	0.00076	0.00645															
2-methylbutanal	1.5	8.22															
decanoic acid	500	2360	702.54														
2-methylpropyl acetate	66	205	73.35														
phenylacetaldehyde	5.2	13.8															
2-phenylethyl acetate	360	788	466.18														
1,1-diethoxyethane	25	50															
ethyl decanoate	122	84.8	9.39														
butanoic acid	2400	1380															
2-methylpropan-1-ol	19000	9600		9710	12710	13170	15380	15840	14250	13590	20420	10530	16940	10950	7000	12610	6540
3-methylbutanoic acid	490	245	489.31														
hexanoic acid	4800	1780	453.81														
octan-1-ol	110	35.2	13.46														
2-methylbutanoic acid	3100	561															
hexan-1-ol	590	35.1	26.79														
benzaldehyde	150	8.37	14.72														
ethyl dodecanoate	3500	35.0	22.64														
2-methylpropanoic acid	60000	448	246.16														
ethyl 2-phenylacetate	155.55	0.66															
butan-1-ol	1900	1.54															

#value in µg/kg was converted to µg/L with a factor of 1.

*value was considered false high and excluded from mean calculation.

		reference no.	19	19	20	20	21	21	22	22	22	22	22	22	22	22	22
		beer sample no.	91	92	93	94	95	96	97	98	99	100	101	102	103	104	105
matrix		mean															
ethanol (%, ALC/VOL)		5.0	5.00	4.80			4.99	4.67	6.50	3.50	5.40	4.80	6.40	4.70	4.80	5.00	6.40
pH		4.5															
odorant	OTC (µg/kg) in water	mean concentration (µg/L)															
ethyl acetate	5	23700	20280	30390													
3-methylbutyl acetate	7.2	2070	990	2360													
ethyl hexanoate	1.2	239															
2-phenylethan-1-ol	140	25700	68000	37590													
3-methylbutan-1-ol	220	30000	50210	39820													
acetaldehyde	16	1800															
ethyl 3-methylbutanoate	0.023	2.41															
dimethyl sulfide	0.30	31.0															
ethyl butanoate	0.76	70.6															
3-methylbutanal	0.50	35.0			4.9	7.5	5.0	3.9	2.30	2.18	3.79	2.51	4.45	5.01	47.23	6.68	8.22
ethyl octanoate	8.7	581															
2-methylpropanal	0.49	28.4							2.42	8.47	14.10	4.40	5.63	11.50	229.00	19.12	17.87
acetic acid	5600	311000															
ethyl 2-methylpropanoate	0.089	3.37															
octanoic acid	190	5930															
butane-2,3-dione	1.0	16.6							12.67	7.17	8.15	20.41	20.48	14.65	52.19	12.90	129.49
phenylacetic acid	68	821															
3-(methylsulfanyl)propan-1-ol	36	421															
ethyl 2-methylbutanoate	0.13	1.30															
3-(methylsulfanyl)propanal	0.43	3.94			2.8	4.9	2.3	2.5									
2-methylbutan-1-ol	1200	10300	15810	9620													
ethyl propanoate	10	85.8															
3-methylbut-2-ene-1-thiol	0.00076	0.00645															
2-methylbutanal	1.5	8.22			2.4	4.7	2.9	2.4	3.18	2.56	3.38	1.80	3.04	7.10	60.41	6.26	6.28
decanoic acid	500	2360															
2-methylpropyl acetate	66	205															
phenylacetaldehyde	5.2	13.8			8.3	14.2	2.6	1.9									
2-phenylethyl acetate	360	788															
1,1-diethoxyethane	25	50															
ethyl decanoate	122	84.8															
butanoic acid	2400	1380															
2-methylpropan-1-ol	19000	9600	13010	8690													
3-methylbutanoic acid	490	245															
hexanoic acid	4800	1780															
octan-1-ol	110	35.2															
2-methylbutanoic acid	3100	561															
hexan-1-ol	590	35.1															
benzaldehyde	150	8.37			1.2	2.0			11.55	13.11	12.86	13.02	12.82	8.72	16.96	8.63	20.35
ethyl dodecanoate	3500	35.0															
2-methylpropanoic acid	60000	448															
ethyl 2-phenylacetate	155.55	0.66															
butan-1-ol	1900	1.54															

#value in µg/kg was converted to µg/L with a factor of 1.
*value was considered false high and excluded from mean calculation.

		reference no.	22	22	22	22	22	22	22	22	22	22	22	22	22	22	22
		beer sample no.	106	107	108	109	110	111	112	113	114	115	116	117	118	119	120
matrix		mean															
ethanol (%, ALC/VOL)		5.0	5.50	5.20	5.50	4.00	4.00	4.00	4.40	5.00	5.10	4.40	5.00	3.80	4.70	7.50	5.20
pH		4.5															
odorant	OTC (µg/kg) in water	mean concentration (µg/L)															
ethyl acetate	5	23700															
3-methylbutyl acetate	7.2	2070															
ethyl hexanoate	1.2	239															
2-phenylethan-1-ol	140	25700															
3-methylbutan-1-ol	220	30000															
acetaldehyde	16	1800															
ethyl 3-methylbutanoate	0.023	2.41															
dimethyl sulfide	0.30	31.0															
ethyl butanoate	0.76	70.6															
3-methylbutanal	0.50	35.0	4.56	9.91	4.03	6.94	7.05	6.11	10.09	7.54	9.91	38.20	13.63	4.33	22.03	13.15	32.38
ethyl octanoate	8.7	581															
2-methylpropanal	0.49	28.4	12.29	30.83	10.09	11.16	11.26	8.72	14.50	18.54	15.53	57.96	38.16	8.15	49.49	35.75	53.28
acetic acid	5600	311000															
ethyl 2-methylpropanoate	0.089	3.37															
octanoic acid	190	5930															
butane-2,3-dione	1.0	16.6	90.62	88.79	19.58	3.44	5.18	4.01	3.61	4.21	4.12	14.12	5.71	6.78	8.17	14.74	16.11
phenylacetic acid	68	821															
3-(methylsulfanyl)propan-1-ol	36	421															
ethyl 2-methylbutanoate	0.13	1.30															
3-(methylsulfanyl)propanal	0.43	3.94															
2-methylbutan-1-ol	1200	10300															
ethyl propanoate	10	85.8															
3-methylbut-2-ene-1-thiol	0.00076	0.00645															
2-methylbutanal	1.5	8.22	4.18	6.03	5.24	4.29	6.62	4.73	9.00	6.95	8.28	34.51	9.92	3.30	25.17	10.84	29.55
decanoic acid	500	2360															
2-methylpropyl acetate	66	205															
phenylacetaldehyde	5.2	13.8															
2-phenylethyl acetate	360	788															
1,1-diethoxyethane	25	50															
ethyl decanoate	122	84.8															
butanoic acid	2400	1380															
2-methylpropan-1-ol	19000	9600															
3-methylbutanoic acid	490	245															
hexanoic acid	4800	1780															
octan-1-ol	110	35.2															
2-methylbutanoic acid	3100	561															
hexan-1-ol	590	35.1															
benzaldehyde	150	8.37	16.12	18.07	8.41	12.81	18.59	12.74	13.66	14.25	14.19	21.76	15.03	11.61	15.91	16.13	15.5
ethyl dodecanoate	3500	35.0															
2-methylpropanoic acid	60000	448															
ethyl 2-phenylacetate	155.55	0.66															
butan-1-ol	1900	1.54															

#value in µg/kg was converted to µg/L with a factor of 1.
*value was considered false high and excluded from mean calculation.

		reference no.	23	23	23	23	23	23	23	23	23	23	24	25	25	26	26
		beer sample no.	121	122	123	124	125	126	127	128	129	130	131	132	133	134	135
matrix		mean															
ethanol (%, ALC/VOL)		5.0												5.1		5.3	5.4
pH		4.5														4.5	4.5
odorant	OTC (µg/kg) in water	mean concentration (µg/L)															
ethyl acetate	5	23700											35140				
3-methylbutyl acetate	7.2	2070											3680	450	410	528	281
ethyl hexanoate	1.2	239											370			365	365
2-phenylethan-1-ol	140	25700											22950				
3-methylbutan-1-ol	220	30000											36470				
acetaldehyde	16	1800												943	1335		
ethyl 3-methylbutanoate	0.023	2.41														3.8	6.8
dimethyl sulfide	0.30	31.0															
ethyl butanoate	0.76	70.6															
3-methylbutanal	0.50	35.0	28.9	25.6	35.7	60.7	92.3	68.7	62.0	66.3	44.8	39.0		11	18	244	317
ethyl octanoate	8.7	581											220				
2-methylpropanal	0.49	28.4	13.0	5.11	7.96	26.6	42.5	26.2	69.7	68.2	46.2	33.9		17	46		
acetic acid	5600	311000															
ethyl 2-methylpropanoate	0.089	3.37															
octanoic acid	190	5930															
butane-2,3-dione	1.0	16.6	23.4	14.5	29.6	13.9	12.9	10.3	14.4	30.1	7.40	12.8		10	24		
phenylacetic acid	68	821															
3-(methylsulfanyl)propan-1-ol	36	421															
ethyl 2-methylbutanoate	0.13	1.30															
3-(methylsulfanyl)propanal	0.43	3.94	24.2				21.8			15.7				1.2	2.6		
2-methylbutan-1-ol	1200	10300															
ethyl propanoate	10	85.8															
3-methylbut-2-ene-1-thiol	0.00076	0.00645															
2-methylbutanal	1.5	8.22	5.35	4.56	5.38	8.89	14.6	9.11	12.4	11.5	7.37	6.48		2.4	4.9		
decanoic acid	500	2360															
2-methylpropyl acetate	66	205															
phenylacetaldehyde	5.2	13.8	12.8	7.33	6.58	4.60	5.29	3.99	21.6	7.36	12.2	2.18		15	38		
2-phenylethyl acetate	360	788											120				
1,1-diethoxyethane	25	50															
ethyl decanoate	122	84.8											140				
butanoic acid	2400	1380															
2-methylpropan-1-ol	19000	9600											14860				
3-methylbutanoic acid	490	245															
hexanoic acid	4800	1780															
octan-1-ol	110	35.2															
2-methylbutanoic acid	3100	561															
hexan-1-ol	590	35.1															
benzaldehyde	150	8.37	1.17	0.627	0.704	0.608	0.691	0.636	1.01	0.853	1.06	0.592				1.3	1.6
ethyl dodecanoate	3500	35.0															
2-methylpropanoic acid	60000	448															
ethyl 2-phenylacetate	155.55	0.66															
butan-1-ol	1900	1.54															

#value in µg/kg was converted to µg/L with a factor of 1.
*value was considered false high and excluded from mean calculation.

		reference no.	26	27	27	27	28	29	29	30	30	30	30	30	30	30	30
		beer sample no.	136	137	138	139	140	141	142	143	144	145	146	147	148	149	150
matrix		mean															
ethanol (%, ALC/VOL)		5.0	5.3							4.9	5.2	5	5	5	5	5.2	5
pH		4.5	4.5					4.46	4.39								
odorant	OTC (µg/kg) in water	mean concentration (µg/L)															
ethyl acetate	5	23700															
3-methylbutyl acetate	7.2	2070	222	473	703	1452	337										
ethyl hexanoate	1.2	239	384														
2-phenylethan-1-ol	140	25700								37000	34000	16000	41000	21000	41000	60000	45000
3-methylbutan-1-ol	220	30000															
acetaldehyde	16	1800		952	202	505											
ethyl 3-methylbutanoate	0.023	2.41	10.8	0.54	0.72	0.28	0.41										
dimethyl sulfide	0.30	31.0															
ethyl butanoate	0.76	70.6															
3-methylbutanal	0.50	35.0	283	36	24	12	9.12										
ethyl octanoate	8.7	581															
2-methylpropanal	0.49	28.4		7.9	5.2		6.68										
acetic acid	5600	311000															
ethyl 2-methylpropanoate	0.089	3.37															
octanoic acid	190	5930															
butane-2,3-dione	1.0	16.6		13.5	7.9	5.6		6.3	2.5								
phenylacetic acid	68	821															
3-(methylsulfanyl)propan-1-ol	36	421															
ethyl 2-methylbutanoate	0.13	1.30		0.61	0.54	0.28	0.45										
3-(methylsulfanyl)propanal	0.43	3.94		2.7	1.8		1.07										
2-methylbutan-1-ol	1200	10300															
ethyl propanoate	10	85.8															
3-methylbut-2-ene-1-thiol	0.00076	0.00645															
2-methylbutanal	1.5	8.22		2.7	2.1		2.39										
decanoic acid	500	2360															
2-methylpropyl acetate	66	205															
phenylacetaldehyde	5.2	13.8		22	15	8	13.12										
2-phenylethyl acetate	360	788					62.5										
1,1-diethoxyethane	25	50															
ethyl decanoate	122	84.8															
butanoic acid	2400	1380															
2-methylpropan-1-ol	19000	9600															
3-methylbutanoic acid	490	245															
hexanoic acid	4800	1780															
octan-1-ol	110	35.2															
2-methylbutanoic acid	3100	561															
hexan-1-ol	590	35.1															
benzaldehyde	150	8.37	2.5				0.96										
ethyl dodecanoate	3500	35.0															
2-methylpropanoic acid	60000	448															
ethyl 2-phenylacetate	155.55	0.66					0.66										
butan-1-ol	1900	1.54															

#value in µg/kg was converted to µg/L with a factor of 1.
*value was considered false high and excluded from mean calculation.

		reference no.	30	30	30	30	30	30	30	30	31	32
		beer sample no.	151	152	153	154	155	156	157	158	159	160
matrix		mean										
ethanol (%, ALC/VOL)		5.0	5	5	4.7	5	5	4.6	5	4.5	4.9	
pH		4.5										
odorant	OTC (µg/kg) in water	mean concentration (µg/L)										
ethyl acetate	5	23700										
3-methylbutyl acetate	7.2	2070									915	
ethyl hexanoate	1.2	239									98.8	
2-phenylethan-1-ol	140	25700	24000	16000	54000	26000	40000	48000	23000	39000		
3-methylbutan-1-ol	220	30000									51200	
acetaldehyde	16	1800										
ethyl 3-methylbutanoate	0.023	2.41									2.55	
dimethyl sulfide	0.30	31.0										
ethyl butanoate	0.76	70.6									64.7	
3-methylbutanal	0.50	35.0										
ethyl octanoate	8.7	581										
2-methylpropanal	0.49	28.4										
acetic acid	5600	311000										
ethyl 2-methylpropanoate	0.089	3.37										
octanoic acid	190	5930										
butane-2,3-dione	1.0	16.6										
phenylacetic acid	68	821									306	
3-(methylsulfanyl)propan-1-ol	36	421										
ethyl 2-methylbutanoate	0.13	1.30									0.54	1.20
3-(methylsulfanyl)propanal	0.43	3.94										
2-methylbutan-1-ol	1200	10300										8170
ethyl propanoate	10	85.8										
3-methylbut-2-ene-1-thiol	0.00076	0.00645										
2-methylbutanal	1.5	8.22										2.85
decanoic acid	500	2360										
2-methylpropyl acetate	66	205										
phenylacetaldehyde	5.2	13.8										
2-phenylethyl acetate	360	788									695	
1,1-diethoxyethane	25	50										
ethyl decanoate	122	84.8										
butanoic acid	2400	1380									15000	
2-methylpropan-1-ol	19000	9600										
3-methylbutanoic acid	490	245									864	
hexanoic acid	4800	1780										
octan-1-ol	110	35.2										
2-methylbutanoic acid	3100	561									968	276.5
hexan-1-ol	590	35.1										
benzaldehyde	150	8.37										
ethyl dodecanoate	3500	35.0										
2-methylpropanoic acid	60000	448									690	
ethyl 2-phenylacetate	155.55	0.66										
butan-1-ol	1900	1.54										

#value in µg/kg was converted to µg/L with a factor of 1.
*value was considered false high and excluded from mean calculation.

Table S4. References on Wine Odorants Used for Data Extraction

no.	reference	no.	reference
1	*J. Agric. Food Chem.* **2002**, *50*, 4048–4054.	85	*Aust. J. Grape Wine Res.* **2021**, *27*, 118–127.
2	*J. Agric. Food Chem.* **2016**, *64*, 3838–3848.	86	*Aust. J. Grape Wine Res.* **2021**, *27*, 348–359.
3	*Food Chem.* **2016**, *202*, 507–517.	87	*Fermentation* **2021**, *7*, 231.
4	*J. Agric. Food Chem.* **2011**, *59*, 8866–8874.	88	*OENO One* **2021**, *55*, 181–195.
5	*J. Agric. Food Chem.* **2019**, *67*, 4011–4022.	89	*LWT* **2021**, *154*, 112711.
6	*J. Agric. Food Chem.* **2014**, *62*, 4528–4536.	90	*Foods* **2021**, *10*, 1164.
7	*Molecules* **2017**, *22*, 1045.	91	*Food Chem.* **2021**, *351*, 129308.
8	*Molecules* **2019**, *24*, 2978.	92	*Fermentation* **2021**, *7*, 97.
9	*Molecules* **2019**, *24*, 1122.	93	*Molecules* **2021**, *26*, 4979.
10	*Molecules* **2018**, *23*, 1096.	94	*Beverages* **2021**, *7*, 29.
11	*Molecules* **2010**, *15*, 9184–9196.	95	*Fermentation* **2020**, *6*, 15.
12	*J. Agric. Food Chem.* **2004**, *52*, 2339–2346.	96	*J. Agric. Food Chem.* **2020**, *68*, 11512–11523.
13	*Eur. Food Res. Technol.* **2008**, *227*, 287–292.	97	*Molecules* **2020**, *25*, 2141.
14	*Aust. J. Grape Wine Res.* **2012**, *18*, 329–343.	98	*J. Sci. Food Agric.* **2020**, *100*, 836–845.
15	*Am. J. Enol. Vitic.* **2012**, *63*, 62–72.	99	*Processes* **2020**, *8*, 1000.
16	*Molecules* **2015**, *20*, 21609–21625.	100	*Int. J. Food Microbiol.* **2020**, *312*, 108373.
17	*Int. J. Food Prop.* **2015**, *18*, 1584–1596.	101	*Food Chem.* **2018**, *259*, 196–206.
18	*Molecules* **2014**, *19*, 12173–12193.	102	*Foods* **2020**, *9*, 1496.
19	*Food Res. Int.* **2013**, *54*, 562–568.	103	*Eur. Food Res. Technol.* **2020**, *246*, 1153–1165.
20	*Food Res. Int.* **2013**, *51*, 482–489.	104	*Foods* **2020**, *9*, 996.
21	*J. Agric. Food Chem.* **2012**, *60*, 5045–5056.	105	*J. Agric. Food Chem.* **2016**, *64*, 870–880.
22	*Food Chem.* **2011**, *129*, 890–898.	106	*Innovative Food Sci. Emerging Technol.* **2020**, *66*, 102521.
23	*J. Agric. Food Chem.* **2011**, *59*, 7916–7924.	107	*Molecules* **2020**, *25*, 3917.
24	*Food Chem.* **2011**, *127*, 516–522.	108	*Food Control* **2019**, *105*, 265–276.
25	*J. Agric. Food Chem.* **2004**, *52*, 3516–3524.	109	*Food Res. Int.* **2019**, *119*, 135–142.
26	*Food Microbiol.* **2009**, *26*, 204–211.	110	*J. Agric. Food Chem.* **2019**, *67*, 3502–3510.
27	*J. Agric. Food Chem.* **2008**, *56*, 10829–10837.	111	*Eur. Food Res. Technol.* **2019**, *245*, 2157–2171.
28	*LWT* **2020**, *129*, 109575.	112	*Molecules* **2019**, *24*, 2777.
29	*Food Chem.* **2016**, *196*, 1048–1057.	113	*Molecules* **2019**, *24*, 836.
30	*J. Agric. Food Chem.* **2013**, *61*, 1542–1553.	114	*Am. J. Enol. Vitic.* **2017**, *68*, 390–399.
31	*Food Chem.* **2014**, *154*, 217–229.	115	*J. Food Process. Preserv.* **2019**, *43*, e13969.
32	*J. Agric. Food Chem.* **2008**, *56*, 2477–2484.	116	*Food Anal. Method.* **2019**, *12*, 1285–1297.
33	*J. Agric. Food Chem.* **2012**, *60*, 2874–2883.	117	*Beverages* **2019**, *5*, 70.
34	*Foods* **2020**, *9*, 1419.	118	*Czech J. Food Sci.* **2019**, *36*, 459–469.
35	*Talanta* **2017**, *174*, 752–766.	119	*Food Chem.* **2019**, *285*, 305–315.
36	*Food Chem.* **2020**, *308*, 125555.	120	*Food Chem.* **2018**, *239*, 495–501.
37	*Food Chem.* **2020**, *312*, 126046.	121	*Food Res. Int*. **2018**, *108*, 119–127.
38	*Foods* **2018**, *7*, 127.	122	*J. Agric. Food Chem.* **2018**, *66*, 2838–2850.
39	*Beverages* **2018**, *4*, 76.	123	*J. Agric. Food Chem.* **2015**, *63*, 3394–3401.
40	*Food Res. Int.* **2018**, *111*, 715–723.	124	*Aust. J. Grape Wine Res.* **2014**, *20*, 223–233.
41	*Food Chem.* **2018**, *240*, 707–716.	125	*J. Food Qual.* **2018**, 512380.
42	*J. Sci. Food Agric.* **2019**, *99*, 904–914.	126	*J. Food Sci. Technol.* **2018**, *55*, 424–430.
43	*Food Chem.* **2018**, *242*, 352–361.	127	*LWT* **2018**, *87*, 515–522.
44	*J. Chromatogr. A* **2018**, *1534*, 130–138.	128	*Food Sci. Technol.* **2018**, *39*, 735–746.
45	*Food Chem.* **2017**, *226*, 41–50.	129	*S. Afr. J. Enol. Vitic.* **2018**, *39*, 235–245.
46	*Front. Chem.* **2017**, *5*, 57.	130	*Am. J. Enol. Vitic.* **2018**, *69*, 266–277.
47	*Chemosens. Percept.* **2017**, *10*, 149–160.	131	*Am. J. Enol. Vitic.* **2018**, *69*, 371–385.
48	*J. Agric. Food Chem.* **2016**, *64*, 1344–1354.	132	*Molecules* **2018**, *23*, 1983.
49	*LWT–Food Sci. Technol.* **2015**, *60*, 400–411.	133	*S. Afr. J. Enol. Vitic.* **2018**, *39*, 246–270.
50	*Food Res. Int.* **2015**, *71*, 108–117.	134	*J. Agric. Food Chem.* **2018**, *66*, 7121–7130.
51	*World J. Microbiol. Biotechnol.* **2015**, *31*, 277–293.	135	*Food Res. Int.* **2018**, *106*, 22–28.
52	*J. Chromatogr. A* **2014**, *1369*, 33–42.	136	*Food Microbiol.* **2018**, *70*, 214–223.
53	*Food Res. Int.* **2014**, *59*, 85–99.	137	*Appl. Microbiol. Biotechnol.* **2015**, *99*, 1911–1922.
54	*J. Agric. Food Chem.* **2014**, *62*, 10015–10027.	138	*J. Agric. Food Chem.* **2007**, *55*, 6674–6684.
55	*Food Res. Int.* **2013**, *53*, 391–402.	139	*Int. J. Food Prop.* **2017**, *20*, 2181–2196.
56	*J. Agric. Food Chem.* **2012**, *60*, 6293–6302.	140	*Eur. Food Res. Technol.* **2017**, *243*, 501–510.
57	*Food Res. Int.* **2012**, *48*, 725–735.	141	*Front. Chem.* **2017**, *5*, 48.
58	*World J. Microbiol. Biotechnol.* **2012**, *28*, 1143–1153.	142	*Food Chem.* **2017**, *224*, 251–261.
59	*LWT–Food Sci. Technol.* **2011**, *44*, 2077–2086.	143	*Food Chem.* **2017**, *214*, 736–744.
60	*Food Technol. Biotechnol.* **2011**, *49*, 529.	144	*LWT* **2017**, *86*, 361–370.
61	*J. Agric. Food Chem.* **2010**, 58, 12417–12425.	145	*Food Res. Int.* **2017**, *98*, 40–49.
62	*Food Res. Int.* **2018**, *112*, 17–24.	146	*LWT* **2017**, *80*, 423–429.
63	*Food Chem.* **2007**, *100*, 1464–1473.	147	*Metabolomics* **2014**, *10*, 556–573.
64	*J. Agric. Food Chem.* **2007**, *55*, 4501–4510.	148	*LWT–Food Sci. Technol.* **2017**, *79*, 111–118.
65	*J. Agric. Food Chem.* **2007**, *55*, 876–881.	149	*Food Chem.* **2017**, *228*, 550–559.
66	*Food Chem.* **2007**, *103*, 536–545.	150	*Am. J. Enol. Vitic.* **2017**, *68*, 30–38.
67	*Food Control* **2007**, *18*, 398–403.	151	*Food Chem.* **2016**, *213*, 90–97.
68	*Food Chem.* **2007**, *103*, 631–640.	152	*Food Chem.* **2012**, *132*, 2155–2164.
69	*Food Chem.* **2006**, *99*, 350–359.	153	*J. Agric. Food Chem.* **2016**, *64*, 608–617.
70	*J. Agric. Food Chem.* **2006**, *54*, 909–915.	154	*Food Chem.* **2016**, *208*, 326–335.
71	*J. Agric. Food Chem.* **2006**, *54*, 3973–3981.	155	*J. Food Sci.* **2016**, *81*, M935–M943.
72	*Food Chem.* **2006**, *95*, 279–289.	156	*Eur. Food Res. Technol.* **2016**, *242*, 609–623.
73	*J. Agric. Food Chem.* **2005**, *53*, 4166–4177.	157	*J. Agric. Food Chem.* **2016**, *64*, 7979–7993.
74	*Ann. Chim.* **2005**, *95*, 383–394.	158	*Food Chem.* **2016**, *196*, 682–693.
75	*J. Agric. Food Chem.* **2003**, *51*, 2700–2707.	159	*Food Chem.* **2016**, 196, 1163–1171.
76	*J. Agric. Food Chem.* **2001**, *49*, 2924–2929.	160	*Molecules* **2016**, *21*, 1485.
77	*J. Sci. Food Agric.* **2000**, *80*, 1659–1667.	161	*Int. J. Food Prop.* **2016**, *19*, 2417–2431.
78	*Food Microbiol.* **2022**, *103*, 103960.	162	*Ferment. Technol.* **2015**, *4*, 1000117.
79	*Molecules* **2021**, *26*, 6010.	163	*Food Chem.* **2015**, *183*, 181–189.
80	*Molecules* **2021**, *26*, 6256.	164	*Int. J. Food Microbiol.* **2015**, *207*, 40–48.
81	*Foods* **2021**, *10*, 2474.	165	*Microchem. J.* **2015**, *122*, 20–28.
82	*Beverages* **2021**, *7*, 49.	166	*Food Res. Int.* **2015**, *69*, 26–37.
83	*Molecules* **2021**, *26*, 2127.	167	*S. Afr. J. Enol. Vitic.* **2015**, *36*, 296–303.
84	*Foods* **2021**, *10*, 1047.	168	*Aust. J. Grape Wine Res.* **2015**, *21*, 425–429.

no.	reference	no.	reference
169	*Sci. Hortic.* **2015**, *194*, 237–245.	211	*J. Food Qual.* **2011**, *34*, 100–110.
170	*Food Chem.* **2014**, *162*, 192–205.	212	*J. Agric. Food Chem.* **2011**, *59*, 12171–12182.
171	*Food Chem.* **2014**, *153*, 52–59.	213	*Food Chem.* **2011**, *127*, 1153–1162.
172	*Eur. Food Res. Technol.* **2015**, *240*, 999–1012.	214	*Food Res. Int.* **2011**, *44*, 397–403.
173	*LWT–Food Sci. Technol.* **2014**, *58*, 35–48.	215	*J. Agric. Food Chem.* **2010**, *58*, 12890–12898.
174	*J. Agric. Food Chem.* **2014**, *62*, 5378–5389.	216	*J. Food Sci.* **2010**, *75*, S206–S211.
175	*Int. J. Food Prop.* **2014**, *17*, 987–1001.	217	*Food Res. Int.* **2010**, *43*, 1423–1428.
176	*Am. J. Enol. Vitic.* **2014**, *65*, 293–304.	218	*Anal. Chim. Acta* **2010**, *660*, 149–157.
177	*Aust. J. Grape Wine Res.* **2014**, *20*, 340–346.	219	*J. Agric. Food Chem.* **2010**, *58*, 12976–12985.
178	*Rev. Chim. (Bucharest)* **2014**, *65*, 168–173	220	*Ann. Microbiol.* **2009**, *59*, 733–740.
179	*Food Chem.* **2013**, *138*, 1696–1705.	221	*Food Chem.* **2009**, *117*, 473–484.
180	*Food Chem.* **2013**, *136*, 224–236.	222	*Int. J. Food Microbiol.* **2009**, *135*, 68–74.
181	*Food Res. Int.* **2013**, *51*, 790–796.	223	*J. Agric. Food Chem.* **2009**, *57*, 4948–4955.
182	*Food Chem.* **2013**, *141*, 1673–1680.	224	*Aust. J. Grape Wine Res.* **2009**, *15*, 238–248.
183	*J. Sci. Food Agric.* **2013**, *93*, 1485–1481.	225	*Food Res. Int.* **2009**, *42*, 1281–1286.
184	*Food Chem.* **2013**, *140*, 245–254.	226	*J. Agric. Food Chem.* **2009**, *57*, 1915–1920.
185	*Vitis* **2013**, *52*, 41–48.	227	*J. Agric. Food Chem.* **2008**, *56*, 9175–9182.
186	*Food Res. Int.* **2013**, *51*, 855–865.	228	*J. Agric. Food Chem.* **2008**, *56*, 4555–4563.
187	*J. Food Sci.* **2013**, *78*, C507–C513.	229	*J. Food Agric. Environ.* **2008**, *6*, 28.
188	*Anal. Chim. Acta* **2012**, *720*, 104–111.	230	*Metab. Eng.* **2008**, *10*, 78–86.
189	*Food Anal. Method.* **2012**, *5*, 1427–1434.	231	*Anal. Chim. Acta* **2008**, *617*, 107–118.
190	*Food Chem.* **2012**, *133*, 284–292.	232	*Innovative Food Sci. Emerging Technol.* **2008**, *9*, 469–476.
191	*J. Agric. Sci. Technol. B* **2012**, *2*, 691–702.	233	*Int. J. Food Microbiol.* **2008**, *121*, 169–177.
192	*J. Agric. Food Chem.* **2012**, *60*, 12371–12383.	234	*Eur. Food Res. Technol.* **2008**, *226*, 1317–1323.
193	*J. Food Compos. Anal.* **2008**, *21*, 724–730.	235	*Food Chem.* **2008**, *108*, 213–219.
194	*Food Chem.* **2012**, *135*, 486–493.	236	*Talanta* **2008**, *76*, 929–935.
195	*Food Biotechnol.* **2012**, *26*, 307–325.	237	*J. Agric. Food Chem.* **2007**, *55*, 5205–5212.
196	*Afr. J. Biotechnol.* **2012**, *11*, 8280–8287.	238	*J. Food Sci.* **2007**, *72*, S314–S318.
197	*Aust. J. Grape Wine Res.* **2012**, *18*, 91–99.	239	*Food Control* **2007**, *18*, 1501–1506.
198	*Food Chem.* **2012**, *133*, 124–131.	240	*J. Chromatogr. A* **2007**, *1165*, 151–157.
199	*J. Agric. Food Chem.* **2012**, *60*, 7050–7063.	241	*J. Food Sci.* **2003**, *68*, 158–163.
200	*Food Sci. Technol. Int.* **2012**, *18*, 103–112.	242	*Anal. Chim. Acta* **2006**, *563*, 145–153.
201	*Acta Agric. Slov.* **2011**, *97*, 285–293.	243	*Anal. Chim. Acta* **2006**, *563*, 165–172.
202	*Int. J. Food Sci. Technol.* **2011**, *46*, 1801–1808.	244	*Food Chem.* **2005**, *90*, 357–363.
203	*Am. J. Enol. Vitic.* **2011**, *62*, 527–535.	245	*J. Agric. Food Chem.* **2005**, *53*, 5682–5690.
204	*Food Res. Int*. **2011**, *44*, 2788–2797.	246	*J. Agric. Food Chem.* **2005**, *53*, 3503–3509.
205	*Food Microbiol.* **2011**, *28*, 873–882.	247	*Food Chem.* **2005**, *92*, 627–635.
206	*Food Chem.* **2011**, *129*, 1193–1200.	248	*J. Agric. Food Chem.* **2004**, *52*, 1241–1247.
207	*Appl. Microbiol. Biotechnol.* **2011**, *91*, 603–612.	249	*Chromatographia* **2004**, *59*, 733–738.
208	*S. Afr. J. Enol. Vitic.* **2011**, *32*, 190–203.	250	*J. Agric. Food Chem.* **2000**, *48*, 885–889.
209	*J. Agric. Food Chem.* **2011**, *59*, 4171–4182.	251	*J. Agric. Food Chem.* **2014**, *62*, 5005–5010.
210	*LWT–Food Sci. Technol.* **2011**, *44*, 866–874.	252	*J. Agric. Food Chem.* **2016**, *64*, 646–652.

Table S5. Wine Samples Used for Data Extraction

no.	wine sample	no.	wine sample
1	Spanish Grenache rosé wine	85	Chinese Cabernet Sauvignon wine
2	Australian Shiraz rosé wine	86	Spanish Maccabeo white wine
3	Australian Cabernet Sauvignon rosé wine	87	Australian Sauvignon Blanc wine 1
4	Australian rosé wine 1	88	Australian Sauvignon Blanc wine 2
5	Australian rosé wine 2	89	Australian Sauvignon Blanc wine 3
6	Australian rosé wine 3	90	Australian Sauvignon Blanc wine 4
7	Australian rosé wine 4	91	Australian Sauvignon Blanc wine 5
8	Australian rosé wine 5	92	Australian Sauvignon Blanc wine 6
9	Australian rosé wine 6	93	Australian Sauvignon Blanc wine 7
10	Australian rosé wine 7	94	Australian Sauvignon Blanc wine 1
11	Australian rosé wine 8	95	Australian Sauvignon Blanc wine 2
12	Australian rosé wine 9	96	Australian Sauvignon Blanc wine 3
13	Australian rosé wine 10	97	Romanian Muscat Ottonel wine 1
14	Australian rosé wine 11	98	Romanian Muscat Ottonel wine 2
15	French rosé wine 1	99	Romanian Muscat Ottonel wine 3
16	French rosé wine 2	100	Romanian Muscat Ottonel wine 4
17	Chinese rosé wine 1	101	Romanian Muscat Ottonel wine 5
18	German Dornfelder red wine	102	Romanian Muscat Ottonel wine 6
19	Australian Cabernet Sauvignon red wine	103	Romanian Muscat Ottonel wine 7
20	Australian Shiraz wine 1	104	Croatian Gewürztraminer wine
21	Australian Shiraz wine 2	105	Chinese Meili wine
22	Chinese Syrah wine 1	106	Chinese Cabernet Sauvignon wine 1
23	Chinese Syrah wine 2	107	Chinese Cabernet Sauvignon wine 2
24	Chinese Marselan red wine	108	Spanish white wine (young)
25	Chinese Cabernet Sauvignon red wine 1	109	Canadian Riesling wine
26	Chinese Cabernet Sauvignon red wine 2	110	Spanish Treixadura white wine (aged 1)
27	Chinese Cabernet Sauvignon red wine 3	111	Spanish Treixadura white wine (aged 2)
28	Chinese Cabernet Sauvignon red wine 4	112	Spanish Treixadura white wine (aged 3)
29	Chinese Cabernet Sauvignon red wine 5	113	Spanish Treixadura white wine (aged 4)
30	Chinese Cabernet Franc wine 1	114	Brazilian Merlot red wine 1
31	Chinese Cabernet Franc wine 2	115	Brazilian Merlot red wine 2
32	Chinese Cabernet Franc wine 3	116	Brazilian Merlot red wine 3
33	Chinese Cabernet Sauvignon wine (young)	117	Brazilian Merlot red wine 4
34	Chinese Cabernet Gernischet wine (young)	118	Brazilian Merlot red wine 5
35	Chinese Chardonnay wine (young)	119	Brazilian Merlot red wine 6
36	USA Chardonnary wine	120	Brazilian Merlot red wine 7
37	Australian Chardonnary wine	121	Brazilian Merlot red wine 8
38	Chinese Chardonnay white wine	122	Brazilian Merlot red wine 9
39	New Zealand Sauvignon Blanc wine 1	123	Brazilian Merlot red wine 10
40	New Zealand Sauvignon Blanc wine 2	124	Brazilian Merlot red wine 11
41	New Zealand Sauvignon Blanc wine 1	125	Brazilian Merlot red wine 12
42	New Zealand Sauvignon Blanc wine 2	126	Spanish Macabeo and Chardonnay blend wine
43	New Zealand Sauvignon Blanc wine 3	127	Croatian Istrian Malvasia white wine 1
44	Australian Sauvignon Blanc wine 1	128	Croatian Istrian Malvasia white wine 2
45	South African Sauvignon Blanc wine 1	129	Australian Shiraz wine
46	French Sauvignon Blanc wine 1	130	Australian Chardonnay wine
47	USA Sauvignon Blanc wine 1	131	Spanish Mencía wine
48	New Zealand Sauvignon Blanc wine 4	132	Spanish Godello white wine 1
49	New Zealand Sauvignon Blanc wine 5	133	Spanish Godello white wine 2
50	New Zealand Sauvignon Blanc wine 6	134	Spanish Godello white wine 3
51	Australian Sauvignon Blanc wine 2	135	Spanish Treixadura white wine 1
52	South African Sauvignon Blanc wine 2	136	Spanish Treixadura white wine 2
53	French Sauvignon Blanc wine 2	137	Spanish Treixadura white wine 3
54	USA Sauvignon Blanc wine 2	138	Croatian Istrian Malvasia wine 1
55	Danish Solaris white wine	139	Croatian Istrian Malvasia wine 2
56	Chinese Cabernet Sauvignon wine 1	140	Croatian Istrian Malvasia wine 3
57	Chinese Cabernet Sauvignon wine 2	141	Italian Sangiovese wine
58	Chinese Cabernet Sauvignon wine 3	142	Spanish red, white and rosé wines
59	Chinese Cabernet Sauvignon wine 4	143	Chinese Chardonnay white wine
60	Chinese Cabernet Sauvignon wine 5	144	German White Riesling wine
61	Spanish Tempranillo red wine 1	145	German Red Riesling wine
62	Spanish Tempranillo red wine 2	146	German Gewürztraminer wine
63	Spanish Tempranillo red wine 3	147	USA Merlot wine
64	Spanish Graciano red wine 1	148	Spanish Tempranillo red wine 1
65	Spanish Graciano red wine 2	149	Spanish Tempranillo red wine 2
66	Spanish Graciano red wine 3	150	Spanish Tempranillo red wine 3
67	Spanish Loureira white wine	151	Spanish and French red wines
68	Spanish Blanco lexítimo white wine	152	Spanish Tempranillo wine
69	Spanish Torrontés white wine	153	Spanish Graciano wine
70	Spanish Treixadura white wine	154	Italian Soave white wine
71	Spanish Albariño white wine	155	Italian Chardonnary white wine
72	Chinese Cabernet Sauvignon wine 1	156	white wine 1
73	Chinese Cabernet Sauvignon wine 2	157	white wine 2
74	Chinese Cabernet Sauvignon wine 3	158	red wine 1 (aged)
75	Chinese Cabernet Sauvignon wine 4	159	red wine 2 (aged)
76	Chinese Merlot wine 1	160	red wine 3 (young)
77	Chinese Merlot wine 2	161	red wine 4 (young)
78	Chinese Merlot wine 3	162	Brazilian Chardonnay wine
79	Chinese Merlot wine 4	163	Spanish red wine
80	Spanish red wine 1	164	Spanish Petit Verdot wine
81	Spanish red wine 2	165	New Zealand Sauvignon Blanc wine
82	Spanish red wine 3	166	USA Chardonnay wine 1
83	Spanish Godello white wine	167	USA Chardonnay wine 2
84	Spanish red wine (aged)	168	USA Chardonnay wine 3

no.	wine sample	no.	wine sample
169	USA Chardonnay wine 4	255	Italian Corvinone wine 1
170	USA Chardonnay wine 5	256	Italian Corvinone wine 2
171	USA Chardonnay wine 6	257	Italian Corvinone wine 3
172	German Riesling wine	258	Italian Corvinone wine 4
173	German Chardonnay wine	259	Italian Corvinone wine 5
174	Italian Amarone wine	260	Italian Corvinone wine 6
175	Australian Shiraz wine 1	261	Italian Corvinone wine 7
176	Australian Shiraz wine 2	262	Italian Corvinone wine 8
177	Spanish Tempranillo red wine	263	Spanish white wine 1
178	Spanish Zalema white wine (young)	264	Spanish white wine 2
179	Spanish red wine 1 (aged)	265	Spanish white wine 3
180	Spanish red wine 2 (aged)	266	Spanish white wine 4
181	Spanish red wine 3 (aged)	267	Spanish white wine 5
182	Spanish Tannat red wine 1 (aged)	268	Spanish rosé wine 1
183	Spanish Tannat red wine 2 (aged)	269	Spanish rosé wine 2
184	Spanish red wine 1 (young)	270	Spanish rosé wine 3
185	Spanish white wine (young)	271	Spanish rosé wine 4
186	Spanish red wine 2 (aged)	272	Italian Lugana white wine 1
187	Spanish red wine 3 (aged)	273	Italian Lugana white wine 2
188	French white wine 1	274	Italian Lugana white wine 3
189	French white wine 2	275	Italian Lugana white wine 4
190	French white wine 3	276	Italian Lugana white wine 5
191	French white wine 4	277	Italian Lugana white wine 6
192	French white wine 5	278	Italian Verdicchio white wine 1
193	Spanish Muscat “a petit grains” wine 1	279	Italian Verdicchio white wine 2
194	Spanish Muscat “a petit grains” wine 2	280	Italian Verdicchio white wine 3
195	Spanish Albillo wine 1	281	Italian Verdicchio white wine 4
196	Spanish Albillo wine 2	282	Italian Verdicchio white wine 5
197	Spanish Albillo wine	283	Italian Verdicchio white wine 6
198	Spanish Chardonnay wine	284	Italian Verdicchio white wine 7
199	Slovak Devín white wine	285	Italian Verdicchio white wine
200	Spanish wine 1	286	Australian Pinot Noir wine
201	Spanish wine 2	287	Australian Shiraz wine
202	Spanish wine 3	288	Chinese Cabernet Sauvignon wine
203	Spanish wine 4	289	Spanish Tempranillo red wine 1 (young)
204	Spanish wine 5	290	Spanish Tempranillo red wine 2 (young)
205	Spanish wine 6	291	Merlot wine
206	Spanish wine 7	292	Spanish Cabernet Sauvignon red wine
207	Spanish wine 8	293	Chinese Cabernet Sauvignon wine 1
208	Spanish wine 9	294	Chinese Riesling wine 1
209	Spanish wine 10	295	Chinese Cabernet Sauvignon wine 2
210	Spanish wine 11	296	Chinese Riesling wine 2
211	Spanish wine 12	297	Australian Chardonnay wine 1
212	Spanish wine 13	298	Australian Chardonnay wine 2
213	Spanish wine 14	299	Australian Chardonnay wine
214	Spanish wine 15	300	Australian Chardonnay wine (aged 1)
215	Spanish wine 16	301	Australian Chardonnay wine (aged 2)
216	Spanish wine 17	302	New Zealand Sauvignon Blanc wine
217	Spanish wine 18	303	Chinese Cabernet Gernischt wine
218	Spanish wine 19	304	French Syrah wine 1
219	Spanish wine 20	305	French Syrah wine 2
220	Spanish Muscat “a petit grains” wine	306	Italian Corvina red wine 1 (young)
221	Spanish red wine (aged 1)	307	Italian Corvina red wine 2 (young)
222	Spanish red wine (aged 2)	308	Italian Corvina red wine 3 (young)
223	Spanish red wine (aged 3)	309	Chilean Carménère wine 1
224	Spanish red wine (aged 4)	310	Chilean Carménère wine 2
225	Spanish red wine (aged 5)	311	Romanian Muscat Ottonel white wine
226	Spanish red wine (aged 6)	312	Romanian Muscat Ottonel white wine (aged 1)
227	Spanish red wine (aged 7)	313	Romanian Muscat Ottonel white wine (aged 2)
228	Spanish red wine (aged 8)	314	Romanian Muscat Ottonel white wine (aged 3)
229	Spanish red wine (aged 9)	315	Romanian Muscat Ottonel white wine (aged 4)
230	Spanish red wine (aged 10)	316	Romanian Muscat Ottonel white wine (aged 5)
231	Spanish red wine (aged 11)	317	Romanian Muscat Ottonel white wine (aged 6)
232	Italian Primitivo red wine 1	318	Romanian Muscat Ottonel white wine (aged 7)
233	Italian Primitivo red wine 2	319	Romanian Muscat Ottonel white wine (aged 8)
234	Italian Aglianico red wine 1	320	Romanian Muscat Ottonel white wine (aged 9)
235	Italian Aglianico red wine 2	321	Australian Viognier wine
236	Italian Cabernet Sauvignon red wine	322	Australian Cabernet Sauvignon wine
237	Italian Merlot red wine	323	Italian Sangiovese wine
238	Spanish red wine (aged)	324	Spanish Petit Verdot red wine 1
239	Spanish red wine (aged)	325	Spanish Petit Verdot red wine 2
240	Spanish red wine (young)	326	Spanish Petit Verdot red wine 3
241	Pinot Noir wine	327	Croatian Sauvignon Blanc wine
242	USA wine 1	328	Australian Shiraz wine
243	USA wine 2	329	Chilean Sauvignon Blanc wine 1
244	USA wine 3	330	Chilean Sauvignon Blanc wine 2
245	USA wine 4	331	Chilean Sauvignon Blanc wine 3
246	German Riesling white wine	332	Chilean Sauvignon Blanc wine 4
247	Italian Corvina wine 1	333	Spanish Verdejo white wine 1
248	Italian Corvina wine 2	334	Spanish Verdejo white wine 2
249	Italian Corvina wine 3	335	South African Chenin Blanc wine 1
250	Italian Corvina wine 4	336	South African Chenin Blanc wine 2
251	Italian Corvina wine 5	337	South African Chenin Blanc wine 3
252	Italian Corvina wine 6	338	South African Chenin Blanc wine 4
253	Italian Corvina wine 7	339	South African Chenin Blanc wine 5
254	Italian Corvina wine 8	340	South African Chenin Blanc wine 6

no.	wine sample	no.	wine sample
341	Spanish Chelva wine 1	427	Australian Chardonnay wine 6 (aged)
342	Spanish Chelva wine 2	428	Australian Chardonnay wine 7
343	Spanish Chelva wine 3	429	Australian Chardonnay wine 8
344	Spanish Chelva wine 4	430	Australian Chardonnay wine 9
345	Spanish Chelva wine 5	431	Australian Chardonnay wine 10
346	Australian red wine 1	432	Australian Chardonnay wine 11 (aged)
347	Australian red wine 2	433	Australian Chardonnay wine 12 (aged)
348	Australian red wine 3	434	Australian Chardonnay wine 13
349	Australian red wine 4	435	Australian Chardonnay wine 14
350	Australian red wine 5	436	Australian Chardonnay wine 15
351	Australian red wine 6	437	Australian Chardonnay wine 16
352	Australian red wine 7	438	Australian Chardonnay wine 17 (aged)
353	Australian red wine 8	439	Australian Chardonnay wine 18 (aged)
354	Australian red wine 9	440	Australian Shiraz wine 1
355	Australian red wine 10	441	Australian Shiraz wine 2
356	Australian white wine 1	442	Australian Shiraz wine 3 (aged)
357	Australian white wine 2	443	Australian Shiraz wine 4
358	Australian white wine 3	444	Australian Shiraz wine 5
359	Australian white wine 4	445	Australian Shiraz wine 6 (aged)
360	Australian white wine 5	446	Australian Shiraz wine 7
361	Australian white wine 6	447	Australian Shiraz wine 8
362	Australian white wine 7	448	Australian Shiraz wine 9 (aged)
363	Australian white wine 8	449	Croatian Pošip white wine
364	Australian white wine 9	450	Croatian Škrlet white wine
365	Australian white wine 10	451	Croatian Malvazija Istarska white wine 1 (after fermentation)
366	Spanish Albariño wine 1	452	Croatian Malvazija Istarska white wine 2 (after fermentation)
367	Spanish Albariño wine 2	453	Croatian Malvazija Istarska white wine 1 (after protein stabilization)
368	Spanish Albariño wine 3	454	Croatian Malvazija Istarska white wine 2 (after protein stabilization)
369	Spanish Albariño wine 4	455	Chinese Cabernet Gernischt red wine
370	Spanish Albariño wine 5	456	Chinese Ecolly white wine
371	Spanish Albariño wine 6	457	Chinese Cabernet Sauvignon wine
372	Chinese Cabernet Sauvignon wine 1	458	Australian and French Viognier wines
373	Chinese Cabernet Sauvignon wine 2	459	Australian Chardonnay wine
374	Chinese Cabernet Sauvignon wine 3	460	Australian Semillon wine 1
375	Chinese Cabernet Sauvignon wine 4	461	Australian Semillon wine 2
376	Chinese Chardonnay white wine	462	Australian Semillon wine 3
377	Chinese Chardonnay white wine (aged 1)	463	Australian Semillon wine 4
378	Chinese Chardonnay white wine (aged 2)	464	Australian Semillon wine 5
379	Chinese Chardonnay white wine (aged 3)	465	Australian Semillon wine 6
380	Chinese Chardonnay white wine (aged 4)	466	Australian Sauvignon Blanc wine
381	Chinese Chardonnay white wine (aged 5)	467	Australian Pinot Blanc wine 1
382	Chinese Chardonnay white wine (aged 6)	468	Australian Pinot Blanc wine 2
383	Chinese Chardonnay white wine (aged 7)	469	Australian Pinot Blanc wine 3
384	Chinese Chardonnay white wine (aged 8)	470	Australian Pinot Blanc wine 4
385	Chinese Chardonnay white wine (aged 9)	471	Portuguese white wine
386	Chinese Chardonnay white wine (aged 10)	472	Portuguese white wine (aged 1)
387	Chinese Chardonnay white wine (aged 11)	473	Portuguese white wine (aged 2)
388	Chinese Chardonnay white wine (aged 12)	474	Portuguese white wine (aged 3)
389	Chinese Chardonnay white wine (aged 13)	475	Portuguese white wine (aged 4)
390	Chinese Chardonnay white wine (aged 14)	476	Portuguese white wine (aged 5)
391	Chinese Chardonnay white wine (aged 15)	477	Portuguese white wine (aged 6)
392	Chinese Chardonnay white wine (aged 16)	478	Cabernet Sauvignon red wine
393	Chinese Chardonnay white wine (aged 17)	479	Chinese Cabernet Sauvignon red wine 1
394	Chinese Chardonnay white wine (aged 18)	480	Chinese Cabernet Sauvignon red wine 2
395	Chinese Chardonnay white wine (aged 19)	481	Chinese Cabernet Sauvignon red wine 3
396	Chinese Chardonnay white wine (aged 20)	482	Chinese Cabernet Sauvignon red wine 4
397	Chinese Chardonnay white wine (aged 21)	483	Chinese Cabernet Sauvignon red wine 5
398	Chilean Carignan wine 1	484	Spanish Tempranillo wine 1
399	Chilean Carignan wine 2	485	Spanish Tempranillo wine 2
400	Chilean Carignan wine 3	486	Trebbiano wine
401	Chilean Carignan wine 4	487	USA Pinot Noir wine 1
402	Chilean Carignan wine 5	488	USA Pinot Noir wine 2
403	Chilean Carignan wine 6	489	USA Pinot Noir wine 3
404	Croatian Malvazija Istarska wine 1	490	Chinese Cabernet Sauvignon wine 1
405	Croatian Malvazija Istarska wine 2	491	Chinese Cabernet Sauvignon wine 2
406	Australian red wine 1	492	Canadian Pinot Gris wine 1
407	Australian red wine 2	493	Canadian Pinot Gris wine 2
408	Australian red wine 3	494	Canadian Pinot Gris wine 3
409	Australian red wine 4	495	Canadian Pinot Gris wine 4
410	Australian red wine 5	496	Canadian Pinot Gris wine 5
411	Australian red wine 6	497	Canadian Pinot Gris wine 6
412	Australian red wine 7	498	Canadian Riesling wine 1
413	Australian red wine 8	499	Canadian Riesling wine 2
414	Australian white wine 1	500	Canadian Riesling wine 3
415	Australian white wine 2	501	Canadian Riesling wine 4
416	Australian white wine 3	502	Canadian Riesling wine 5
417	Australian white wine 4	503	Canadian Riesling wine 6
418	Australian white wine 5	504	Australian Cabernet Sauvignon wine
419	Australian white wine 6	505	Pinot Noir wine
420	Australian white wine 7	506	Spanish Viura-Malvasía white wine
421	Australian white wine 8	507	Spanish Tempranillo wine
422	Australian Chardonnay wine 1	508	Spanish Macabeo wine 1
423	Australian Chardonnay wine 2	509	Spanish Macabeo wine 2
424	Australian Chardonnay wine 3	510	Spanish Macabeo wine 3
425	Australian Chardonnay wine 4	511	Chinese Cabernet Gernischet wine 1
426	Australian Chardonnay wine 5 (aged)	512	Chinese Cabernet Gernischet wine 2

no.	wine sample	no.	wine sample
513	Italian Aglianico red wine	599	Brazilian Sauvignon Blanc wine 1
514	Australian Shiraz wine 1	600	Brazilian Sauvignon Blanc wine 2
515	Australian Shiraz wine 2	601	Brazilian Vermentino wine 1
516	Croatian Teran red wine	602	Brazilian Vermentino wine 2
517	USA Pinot Noir wine 1	603	Brazilian Viogner wine 1
518	USA Pinot Noir wine 2	604	Brazilian Viogner wine 2
519	USA Pinot Noir wine 3	605	white wine 1
520	USA Pinot Noir wine 4	606	white wine 2
521	Chinese Ecolly white wine	607	white wine 3
522	Spanish Airén white wine	608	white wine 4
523	Spanish Airén white wine (aged)	609	Chinese Chardonnay wine (aged 1)
524	Australian Sauvignon Blanc wine 1	610	Chinese Chardonnay wine (aged 2)
525	Australian Sauvignon Blanc wine 2	611	Chinese Chardonnay wine (aged 3)
526	Australian Sauvignon Blanc wine 3	612	Chinese Chardonnay wine (aged 4)
527	Australian Sauvignon Blanc wine 4	613	Chinese Chardonnay wine (aged 5)
528	Australian Sauvignon Blanc wine 5	614	Portuguese Vinhão wine 1
529	Australian Sauvignon Blanc wine 6	615	Portuguese Vinhão wine 2
530	New Zealand Sauvignon Blanc wine	616	Chinese Merlot wine 1
531	Spanish Verdejo white wine	617	Chinese Cabernet Sauvignon wine 1
532	Chinese Ecolly white wine	618	Chinese Merlot wine 2
533	Spanish Sauvignon Blanc wine 1	619	Chinese Cabernet Sauvignon wine 2
534	Spanish Sauvignon Blanc wine 2	620	Italian Groppello red wine 1
535	Spanish Sauvignon Blanc wine 3	621	Italian Groppello red wine 2
536	Spanish Sauvignon Blanc wine 4	622	Australian Pinot Noir wine 1
537	Spanish red wine (young 1)	623	Australian Pinot Noir wine 2
538	Spanish red wine (young 2)	624	Australian Pinot Noir wine 3
539	Italian Negroamaro red wine 1	625	Australian Pinot Noir wine 4
540	Italian Negroamaro red wine 2	626	Spanish Macabeo white wine
541	Italian Negroamaro red wine 3	627	Italian Negroamaro wine 1
542	Italian Negroamaro red wine 4	628	Italian Negroamaro wine 2
543	Italian Negroamaro red wine 5	629	French Cabernet Sauvignon wine 1
544	Italian Negroamaro red wine 6	630	French Cabernet Sauvignon wine 2
545	Italian Negroamaro red wine 7	631	French Cabernet Sauvignon wine 3
546	Italian Negroamaro red wine 8	632	French Cabernet Sauvignon wine 4
547	Italian Negroamaro red wine 9	633	French Cabernet Sauvignon wine 5
548	Italian Negroamaro red wine 10	634	French Cabernet Sauvignon wine 6
549	Spanish red wine	635	French Cabernet Sauvignon wine 7
550	Spanish rosé and white wines	636	French Cabernet Sauvignon wine 8
551	New Zealand Sauvignon Blanc wine 1	637	French Cabernet Sauvignon wine 9
552	New Zealand Sauvignon Blanc wine 2	638	French Cabernet Sauvignon wine 10
553	New Zealand Sauvignon Blanc wine 3	639	Spanish Grenache rosé wine (aged 1)
554	New Zealand Sauvignon Blanc wine 4	640	Spanish Grenache rosé wine (aged 2)
555	New Zealand Sauvignon Blanc wine 5	641	Spanish Grenache rosé wine (aged 3)
556	Chinese Pinot Noir wine	642	Spanish Grenache rosé wine (aged 4)
557	Chinese Merlot wine 1	643	French red wine (young)
558	Chinese Merlot wine 2	644	French red wine (aged)
559	South African Sauvignon Blanc wine	645	French Beaujolais Nouveau wine
560	South African Sauvignon Blanc wine (aged 1)	646	French white wine (young)
561	South African Sauvignon Blanc wine (aged 2)	647	French white wine (aged)
562	South African Sauvignon Blanc wine (aged 3)	648	French rosé wine
563	South African Sauvignon Blanc wine (aged 4)	649	Turkish Çalkarası rosé wine
564	South African Sauvignon Blanc wine (aged 5)	650	Romanian Sauvignon white wine (young)
565	Australian rosé wine 1	651	Romanian Riesling Italian white wine (young)
566	Australian rosé wine 2	652	Romanian Feteasca Regala white wine (young)
567	Australian rosé wine 3	653	Romanian Muscat Ottonel white wine (young)
568	Australian rosé wine 4	654	Romanian Chardonnay white wine (young)
569	Australian rosé wine 5	655	Romanian Merlot red wine (young)
570	Australian rosé wine 6	656	Romanian Cabernet Sauvignon red wine (young)
571	Australian rosé wine 7	657	Romanian Burgundy red wine (young)
572	Australian rosé wine 8	658	Romanian Feteasca Neagra red wine (young)
573	Australian rosé wine 9	659	Romanian Pinot Noir red wine (young)
574	Australian rosé wine 10	660	Australian Cabernet Sauvignon wine 1
575	Australian rosé wine 11	661	Australian Cabernet Sauvignon wine 2
576	Australian rosé wine 12	662	Australian Cabernet Sauvignon wine 3
577	Australian rosé wine 13	663	Australian Cabernet Sauvignon wine 4
578	Australian rosé wine 14	664	Australian Cabernet Sauvignon wine 5
579	Australian rosé wine 15	665	Spanish red wine 1 (young)
580	Australian rosé wine 16	666	Spanish red wine 2 (young)
581	Australian rosé wine 17	667	Spanish red wine 1 (aged 1)
582	Australian rosé wine 18	668	Spanish red wine 2 (aged 1)
583	Australian rosé wine 19	669	Spanish red wine 1 (aged 2)
584	Australian rosé wine 20	670	Spanish red wine 2 (aged 2)
585	Australian rosé wine 21	671	Spanish red wine 3 (aged 1)
586	Australian rosé wine 22	672	Spanish red wine 4 (aged 1)
587	Australian rosé wine 23	673	Spanish red wine 3 (aged 2)
588	Australian rosé wine 24	674	Spanish red wine 4 (aged 2)
589	Australian rosé wine 25	675	Spanish red wine 3 (aged 3)
590	Australian rosé wine 26	676	Spanish red wine 4 (aged 3)
591	Australian Granite Belt Verdelho white wine	677	Spanish Pedro Ximenez white wine (young)
592	Chilean White Muscat wine	678	Australian Cabernet Sauvignon wine
593	Chinese Cabernet Sauvignon wine	679	Australian Chardonnay wine
594	Spanish Monastrell red wine	680	Spanish Tempranillo wine
595	New Zealand Sauvignon Blanc wine 1	681	Chinese Cabernet Gernischt wine
596	New Zealand Sauvignon Blanc wine 2	682	Chinese Meili red wine
597	French Sauvignon Blanc wine	683	Chinese Cabernet Sauvignon wine (young)
598	French Merlot wine	684	Spanish Chardonnay white wine (young)

no.	wine sample	no.	wine sample
685	Spanish Chardonnay white wine (aged)	771	Australian Shiraz wine 2
686	Spanish Mencía red wine	772	Australian Chardonnay wine
687	Spanish Merenzao red wine	773	Spanish red wine (aged)
688	Spanish Sousón red wine	774	Italian Raboso Piave red wine (aged 1)
689	Spanish Brancellao red wine	775	Italian Raboso Piave red wine (aged 2)
690	Spanish Mouratón red wine	776	Italian Raboso Piave red wine (aged 3)
691	Hungarian Kékfrankos red wine 1	777	Italian Raboso Piave red wine (aged 4)
692	Hungarian Kékfrankos red wine 2	778	Italian Raboso Piave red wine (aged 5)
693	Hungarian Kékfrankos red wine 3	779	Italian Raboso Piave red wine (aged 6)
694	Hungarian Kékfrankos red wine 4	780	Italian Raboso Piave red wine (aged 7)
695	Hungarian Kékfrankos red wine 5	781	Italian Raboso Piave red wine (aged 8)
696	Spanish rosé wine 1	782	Italian Raboso Piave red wine (aged 9)
697	Spanish rosé wine 2	783	Italian Raboso Piave red wine (aged 10)
698	Thailandian Muscat wine 1	784	Italian Raboso Piave red wine (aged 11)
699	Thailandian Muscat wine 2	785	Italian Raboso Piave red wine (aged 12)
700	French and Swiss red wines	786	Italian Raboso Piave red wine (aged 13)
701	Spanish Tempranillo red wine	787	Italian Raboso Piave red wine (aged 14)
702	New Zealand Sauvignon Blanc wine	788	Italian Raboso Piave red wine (aged 15)
703	New Zealand Sauvignon Blanc wine 1 (aged 1)	789	Australian Shiraz wine
704	New Zealand Sauvignon Blanc wine 1 (aged 2)	790	Greek Assyrtiko white wine 1
705	New Zealand Sauvignon Blanc wine 1 (aged 3)	791	Greek Assyrtiko white wine 2
706	New Zealand Sauvignon Blanc wine 2	792	Croatian Sauvignon Blanc wine
707	New Zealand Sauvignon Blanc wine 3	793	Croatian Riesling wine
708	New Zealand Sauvignon Blanc wine 4	794	Spanish Parellada white wine
709	New Zealand Sauvignon Blanc wine 5	795	Spanish Monastrell red wine 1
710	New Zealand Sauvignon Blanc wine 6	796	Spanish Monastrell red wine 2
711	New Zealand Sauvignon Blanc wine 7	797	Spanish Monastrell red wine 3
712	Singaporean Sugraone wine	798	Spanish Monastrell red wine 4
713	Chinese Cabernet Sauvignon wine 1	799	Spanish Monastrell red wine 5
714	Chinese Cabernet Sauvignon wine 2	800	Spanish Monastrell red wine 6
715	New Zealand Sauvignon Blanc wine 1 (aged)	801	Spanish Monastrell red wine 7
716	New Zealand Sauvignon Blanc wine 2 (aged)	802	Spanish Parellada wine
717	New Zealand Sauvignon Blanc wine 3 (aged)	803	Spanish Parellada wine (aged 1)
718	Spanish Albariño wine	804	Spanish Parellada wine (aged 2)
719	Spanish Verdejo white wine 1	805	Spanish Parellada wine (aged 3)
720	Spanish Verdejo white wine 2	806	Spanish Parellada wine (aged 4)
721	Spanish Verdejo white wine 3	807	Spanish Moscatell wine 1
722	Spanish Tempranillo wine 1	808	Spanish Moscatell wine 2
723	Spanish Tempranillo wine 2	809	Spanish 'claret' wine
724	Spanish Cabernet Sauvignon wine 1	810	Spanish rosé wine
725	Spanish Cabernet Sauvignon wine 2	811	Spanish red wine
726	Slovenian Zelen wine	812	Spanish Chardonnay wine 1
727	Slovenian Ribolla Gialla white wine	813	Spanish Chardonnay wine 2
728	Slovenian Malvasia Istriana white wine	814	Spanish Zalema white wine (young)
729	Spanish red wine 1 (young)	815	Spanish Palomino Fino white wine (young)
730	Spanish red wine 2	816	Spanish Colombard white wine (young)
731	New Zealand Sauvignon Blanc wine	817	Spanish Syrah red wine (young)
732	Austrian Sauvignon Blanc wine	818	Italian Aglianico del Vulture wine 1
733	French Sauvignon Blanc wine	819	Italian Aglianico del Vulture wine 2
734	wine 1	820	Italian Aglianico del Vulture wine 3
735	wine 2	821	Italian Aglianico del Vulture wine 4
736	wine 3	822	Italian Aglianico del Vulture wine 5
737	Italian Sauvignon Blanc wine	823	Italian Aglianico del Vulture wine 6
738	Italian Sauvignon Blanc wine (aged 1)	824	Italian Aglianico del Vulture wine 7
739	Italian Sauvignon Blanc wine (aged 2)	825	Italian Aglianico del Vulture wine 8
740	Australian Chardonnay wine	826	Italian Aglianico del Vulture wine 9
741	Slovenian Zelen white wine	827	Italian Aglianico del Vulture wine 10
742	Slovenian Ribolla Gialla white wine	828	Italian Aglianico del Vulture wine 11
743	Slovenian Malvasia Istriana white wine	829	Italian Aglianico del Vulture wine 12
744	Spanish Chardonnay wine	830	Italian Aglianico del Vulture wine 13
745	Spanish Chardonnay wine (aged 1)	831	Italian Aglianico del Vulture wine 14
746	Spanish Chardonnay wine (aged 2)	832	Italian Aglianico del Vulture wine 15
747	Spanish Cencibel red wine	833	Italian Aglianico del Vulture wine 16
748	Spanish Zalema white wine	834	Italian Aglianico del Vulture wine 17
749	Spanish Airén white wine	835	Italian Aglianico del Vulture wine 18
750	Spanish Listán Blanco white wine 1	836	Portuguese white wine 1
751	Spanish Listán Blanco white wine 2	837	Portuguese white wine 2
752	Spanish Gual white wine 1	838	Portuguese white wine 3
753	Spanish Gual white wine 2	839	Portuguese white wine 4
754	Spanish Godello white wine 1	840	Spanish Parellada white wine
755	Spanish Godello white wine 2	841	Spanish Parellada white wine (aged 1)
756	Spanish Godello white wine 3	842	Spanish Parellada white wine (aged 2)
757	Spanish Godello white wine 4	843	Spanish red and white wines
758	USA Merlot Wine 1	844	Spanish Airén wine
759	USA Merlot Wine 2	845	Spanish Macabeo wine
760	USA Merlot Wine 3	846	Spanish white wine (young)
761	Chardonnay wine	847	Spanish rosé wine (young)
762	white, red and rosé wines	848	Spanish red wine (young)
763	Australian Semillon wine 1	849	Spanish wine 1
764	Australian Semillon wine 2	850	Spanish wine 2
765	Spanish white wine	851	Spanish wine 3
766	Spanish red wine	852	Spanish wine 4
767	Colombian Montepulciano red wine	853	Spanish wine 5
768	Spanish Mencía red wine	854	Spanish wine 6
769	Spanish Bobal red wine	855	Spanish wine 7
770	Australian Shiraz wine 1	856	Spanish wine 8

no.	wine sample	no.	wine sample
857	Spanish wine 9	881	French Muscadet wine 2
858	Spanish wine 10	882	French Muscadet wine 3
859	Spanish wine 11	883	French Muscadet wine 4
860	Spanish wine 12	884	French Muscadet wine 5
861	Spanish wine 13	885	French Muscadet wine 6
862	Spanish wine 14	886	Spanish Albillo wine
863	Spanish wine 15	887	Spanish Airén wine
864	Spanish wine 16	888	Spanish Macabeo wine
865	Spanish wine 17	889	Spanish Chardonnay wine
866	Spanish wine 18	890	Spanish Airén white wine 1
867	Spanish wine 19	891	Spanish Airén white wine 2
868	Spanish wine 20	892	Spanish Airén white wine 3
869	Spanish wine 21	893	Spanish wine 1
870	Spanish wine 22	894	Spanish wine 2
871	Spanish wine 23	895	Spanish wine 3
872	Spanish Mencia wine 1	896	Spanish wine 4
873	Spanish Mencia wine 2	897	Spanish wine 5
874	Spanish Albariño white wine	898	Spanish Airén wine
875	Spanish Godello white wine	899	red wine 1
876	Spanish Malvasía white wine	900	red wine 2
877	Spanish Parellada white wine	901	red wine 3
878	Spanish Treixadura white wine	902	red wine 4
879	Spanish Verdejo white wine	903	red wine
880	French Muscadet wine 1	904	white wine

Table S6. Individual Odorant Concentrations, Ethanol Concentrations, and pH in the Selected Wine Samples (cf. Table S5) as Extracted from the Literature (cf. Table S4)

		reference no.	1	2	2	3	3	3	3	3	3	3	3	3	3	3	3
		wine sample no.	1	2	3	4	5	6	7	8	9	10	11	12	13	14	15
matrix		mean															
ethanol (%, ALC/VOL)		12.9		11.7	11.7	12.4	12.7	13.4	12.6	13.2	12.4	11.9	12.9	14.0	13.4	11.7	12.7
pH		3.4		3.10	3.17	3.26	3.16	3.29	3.18	3.46	3.69	3.39	3.29	3.39	3.70	3.35	3.44
odorant	OTC (µg/kg) in water	mean concentration (µg/L)															
ethyl acetate	5	69100	39000			49594	112120	101760	116710	131070	164620	148590	109570	92050	187440	77370	109090
acetaldehyde	16	49100	65														
butane-2,3-dione	1.0	1400	60	626.4	1297												
ethyl hexanoate	1.2	1570	542	1940	699	1466	1776	1037	1676	1403	567	1372	1068	1217	1504	1359	771
ethyl 3-methylbutanoate	0.023	27.5	3.1	11.1	21.1	45.2	18.1	30.7	30	31.3	13.2	7.9	54.3	53.5	48.3	29.7	36.3
ethyl 2-methylpropanoate	0.089	93.5	17.9	39.8	45.6												
3-methylbutan-1-ol	220	172000	171200	136977	168369	35148	61748	68921	30183	33812	41670	39321	75692	60934	112550	46258	78979
3-methylbutyl acetate	7.2	3650	1260	3235	2623	968	10035	1204	6502	4409	5819	10730	2462	2679	8090	5569	6623
ethyl butanoate	0.76	374	196	410.3	448.4	373	456	362	618	534	143	287	280	341	666	452	258
ethyl 2-methylbutanoate	0.13	42.7	1.2	0.66	1.39	30.9	8.2	20.4	16.5	15.5	10.3	6.9	58.6	35.4	37	18.6	17.1
ethyl octanoate	8.7	2460	206	5587	2903	3273	4333	2614	5009	3484	1535	3392	2682	3728	3826	3703	2676
3-methylbutanal	0.50	119		5.8	11.0												
2-phenylethan-1-ol	140	28700	21600	12653	12520	8993	6316	11574	6035	6394	6978	4086	8451	11091	16741	7470	18818
2-methylpropanal	0.49	36.5		12.0	16.0												
2-methylbutan-1-ol	1200	70100															
dimethyl sulfide	0.30	14.1															
acetic acid	5600	219000	80000	189115	95969	133000	105390	128110	127810	146370	282020	280650	178110	150910	232920	92180	195550
3-(methylsulfanyl)propan-1-ol	36	1360	1807	311	424												
3-(methylsulfanyl)propanal	0.43	14.6			0.43												
ethyl propanoate	10	295															
octanoic acid	190	5580	2560	7412	3001	1925	4005		5569	2879		3070	1003	1649	2514	4244	
3-hydroxybutan-2-one	590	16600	830	646.0	322.1												
2-methylbutanal	1.5	40.2															
phenylacetic acid	68	452	40														
ethyl decanoate	122	741	500	3407	1194	1987	4258	791	4133	1623	544	1519	982	2749	2100	2544	1418
decanoic acid	500	2460	620	3372	2099	98.5	285	133	369	156	166	253	181	250	303	360	146
hexan-1-ol	590	2710	2230	2754	2191	4714	3702	7606	8554	3234	3126	8619	1488	2047	7275	1448	1058
phenylacetaldehyde	5.2	21.5		10.00	8.40												
2-phenylethyl acetate	360	682	81	143.8	53.8	119	15.4	233	184	187	193	177	88.4	117	250		184
2-methylpropan-1-ol	19000	33000	49800	6800	11540	1369	1508	4631	1509		7165	976	5457	3747	6261	5258	2395
3-methylbutanoic acid	490	814	687	93.7	114.2												
2-methylpropyl acetate	66	101	83.4														
hexanoic acid	4800	4060	2080	4236	1777	3890	3969	1922	4352	3768	1846	3923	2862	3038	3347	4443	2059
benzaldehyde	150	108		105.0	81.0	125	132	83	107	104	37.2	85.9	92.3	117	50.5	114	124
butan-1-ol	1900	1120		619	531												
butanoic acid	2400	1180	1842	1780	850.6												
octan-1-ol	110	44.7				36.7	30.2	31.1	17.1	22.4	23	12.8	7.2	28.6	45.4	11.4	14.1
ethyl 2-phenylacetate	155.55	53.8				5.8	3.4	5	5.5	7.9	4.4	2.1	7.2	7.3	19.9	4.7	12.1
2-methylbutanoic acid	3100	545															
ethyl dodecanoate	3500	269		61.9	46.4	44	232	164	230	184	184	220	43.8	270	150	191	91.2
propanoic acid	20000	1490															
2-methylpropanoic acid	60000	2180	1323	516.9	437.3												

		reference no.	3	3	4	5	6	6	7	7	8	9	9	9	9	9	10
		wine sample no.	16	17	18	19	20	21	22	23	24	25	26	27	28	29	30
matrix		mean															
ethanol (%, ALC/VOL)		12.9	12.7	12.2	13		14.6	14.2	14.1	13.6		12.8	12.7	12.4	12.7	13.2	11.8
pH		3.4	3.36	3.52	3.8		3.50	3.50	3.48	3.56		3.29	3.28	3.32	3.26	3.32	3.2
odorant	OTC (µg/kg) in water	mean concentration (µg/L)															
ethyl acetate	5	69100	111150	81962			164400	103100	52086	81445		1670.70	2301.28	1541.92	1760.18	3247.69	64571.6
acetaldehyde	16	49100			12100				22343	31905							
butane-2,3-dione	1.0	1400			2040	2240					1909.2	730.61	227.67	198.72	44.23	251.12	183.2
ethyl hexanoate	1.2	1570	828	953	307	480	369	391	533	151		15.33	33.85	29.87	28.23	23.73	51090.6
ethyl 3-methylbutanoate	0.023	27.5	77.5	30.9	55.1	73.0	54	100	131	82	56.8	5.69	34.43	16.35	14.16	12.59	
ethyl 2-methylpropanoate	0.089	93.5			385	259	207	353	264	164	1847.6						
3-methylbutan-1-ol	220	172000	82555	46062	307200	275000	161250	208400	123435	105594	382940.8	1677.67	1866.42	1851.10	1651.68	2010.54	51052.5
3-methylbutyl acetate	7.2	3650	3554	1009		267	508	465	408	244	1667.2	166.00	940.16	423.69	355.32	342.54	2238.0
ethyl butanoate	0.76	374	285	293	239	250	305	234	167	120	400.8						
ethyl 2-methylbutanoate	0.13	42.7	40.7	19.4	50.6	121	36	76	79.6	48.2	186.7						
ethyl octanoate	8.7	2460	2775	2135		448	378	439	275	58		4420.23	4378.07	4130.39	3107.50	3175.38	4383.9
3-methylbutanal	0.50	119															
2-phenylethan-1-ol	140	28700	20589	6663	79040	30400	26350	56450	13793	9628	30823.2	12543.37	16110.19	16156.39	18903.91	19803.92	11434.8
2-methylpropanal	0.49	36.5															
2-methylbutan-1-ol	1200	70100			77680	99800	40150	76000									
dimethyl sulfide	0.30	14.1					130	37									
acetic acid	5600	219000	199290	224520	641900	546000	680000	510000				32912.94	31904.99	26595.79	15357.73	26864.24	
3-(methylsulfanyl)propan-1-ol	36	1360				3420	2018	3061			1514.2	6294.29	9312.64	14386.98	11737.56	9413.23	2041.4
3-(methylsulfanyl)propanal	0.43	14.6			3.3	2.50					5.1	20.65	23.73	21.57	21.87	20.32	
ethyl propanoate	10	295				358	723	318	204	184							
octanoic acid	190	5580				2150	1480	1680	650	579		2183.02	806.54	949.62	1192.04	1732.84	1834.4
3-hydroxybutan-2-one	590	16600										483.35	529.67	578.98	602.23	411.03	2343.2
2-methylbutanal	1.5	40.2															
phenylacetic acid	68	452			102	97.4											
ethyl decanoate	122	741	2138				127	115	99.1	16.3		384.84	806.36	1115.42	540.06	522.64	861.8
decanoic acid	500	2460	180	38	476	1030	224	350	122	72							501.0
hexan-1-ol	590	2710	2206				2030	2008	1046	1279							1304.2
phenylacetaldehyde	5.2	21.5															55.0
2-phenylethyl acetate	360	682	41.1	28.7	53.5	56.6	35	53	33.3	20.3	114.7	38.17	78.57	69.42	49.43	55.21	8.1
2-methylpropan-1-ol	19000	33000	2942	12530		59600	26750	56200	52872	47188	10999.4	2476.18	2354.35	1292.78	1384.78	3701.12	10060.8
3-methylbutanoic acid	490	814			1740	823	438	946			465.2						
2-methylpropyl acetate	66	101					67	85	78.1	61.0							
hexanoic acid	4800	4060	2295	2936			1840	1900	1489	1076							1737.4
benzaldehyde	150	108	131	107							77.3	170.89	101.30	132.20	139.71	197.35	42.0
butan-1-ol	1900	1120					2625	1939									1332.0
butanoic acid	2400	1180			1380	1350	433	174	550	484		1286.30	1279.59	846.21	741.70	687.95	
octan-1-ol	110	44.7	18.3	17.1													17.6
ethyl 2-phenylacetate	155.55	53.8	21.6	17.3					5.51	5.92		9.53	31.16	11.91	8.71	5.29	
2-methylbutanoic acid	3100	545			957	2116	335	907									
ethyl dodecanoate	3500	269	211	38.6							37.1						698.5
propanoic acid	20000	1490															
2-methylpropanoic acid	60000	2180			1690	1370	929	1634	730	544							

		reference no.	10	10	11	11	11	12	12	13	14	14	15	15	15	15	15
		wine sample no.	31	32	33	34	35	36	37	38	39	40	41	42	43	44	45
matrix		mean															
ethanol (%, ALC/VOL)		12.9	11.6	12.1	11.8	12.2	12.7	14.5	13.5		12.8	13.9					
pH		3.4	3.2	3.1	3.0	3.1	3.4				3.35	3.23					
odorant	OTC (µg/kg) in water	mean concentration (µg/L)															
ethyl acetate	5	69100	107130.0	123756.8	52205.7	69820.2	32920.7			5000							
acetaldehyde	16	49100															
butane-2,3-dione	1.0	1400	805.8	791.2					172.7								
ethyl hexanoate	1.2	1570	63860.7	64627.6	4107.2	2985.1	5535.6	757.2	737.5	650	1113	1126					
ethyl 3-methylbutanoate	0.023	27.5						9.2	19.9		16	17					
ethyl 2-methylpropanoate	0.089	93.5						72.2	99.9		60	58					
3-methylbutan-1-ol	220	172000	55280.9	67458.6	185587.5	144784.9	103021.5	253591	356725	150000	192000	192000					
3-methylbutyl acetate	7.2	3650	1569.5	2051.5	14733.8	13999.9	24849.9	943.7	163.5	60	2567	2665	2531	2515	2892	1430	1732
ethyl butanoate	0.76	374		265.8				263.0	341.5	680	346	345	548	576	510	381	480
ethyl 2-methylbutanoate	0.13	42.7															
ethyl octanoate	8.7	2460	6263.5	5741.8	5107.9	4284.0	13767.8			1200	1062	1121	1371	1455	1631	1116	864
3-methylbutanal	0.50	119															
2-phenylethan-1-ol	140	28700	31433.8	30600.5	14504.8	18164.6	9165.8	12415	24971	34000	24200	24600	38000	33000	43000	22000	17000
2-methylpropanal	0.49	36.5															
2-methylbutan-1-ol	1200	70100															
dimethyl sulfide	0.30	14.1									4.3	4.5					
acetic acid	5600	219000			18901.2	11538.3	23016.1	434232	489370								
3-(methylsulfanyl)propan-1-ol	36	1360	2796.6	2900.4	1990.2	2006.8		563.4	795.6		1103	1061					
3-(methylsulfanyl)propanal	0.43	14.6															
ethyl propanoate	10	295															
octanoic acid	190	5580	4094.4	3875.1	5205.9	3848.5	11208.7			26000	95	92					
3-hydroxybutan-2-one	590	16600	4009.7	3064.3	3378.5	5595.8	2645.7										
2-methylbutanal	1.5	40.2															
phenylacetic acid	68	452						34.6	90.0								
ethyl decanoate	122	741	1148.7	1200.5	1451.2	1522.5	7962.0			300	312	352	368	362	464	271	194
decanoic acid	500	2460	1483.5	1404.1	2877.7	2477.4	12089.7			2600	26	24					
hexan-1-ol	590	2710	2851.6	1559.3	4017.7	2542.8	1163.7			1200	2411	2372	3081	2901	3105	2710	1672
phenylacetaldehyde	5.2	21.5															
2-phenylethyl acetate	360	682	31.8	36.9		37.2	310.4			650							
2-methylpropan-1-ol	19000	33000	14970.7	19902.2	22004.1	19778.1	9379.0			4900	79000	80000					
3-methylbutanoic acid	490	814				1961.2	898.0	588.0	561.6								
2-methylpropyl acetate	66	101					339.4				89	86					
hexanoic acid	4800	4060	4113.2	3961.0	1709.8	1155.1	1714.3			5300	396	391					
benzaldehyde	150	108	11.7	11.4	348.6	95.2	349.9										
butan-1-ol	1900	1120	1487.7	1207.7	1587.6	1083.4	945.2			240							
butanoic acid	2400	1180						1839	1611								
octan-1-ol	110	44.7	39.8	3.3	44.2	25.2	17.0										
ethyl 2-phenylacetate	155.55	53.8															
2-methylbutanoic acid	3100	545															
ethyl dodecanoate	3500	269	1672.4	3375.1	222.7	264.5	2028.6			90							
propanoic acid	20000	1490			213.3	907.2											
2-methylpropanoic acid	60000	2180	24.7		2916.4	2335.3	1798.4										

		reference no.	15	15	15	15	15	15	15	15	15	16	17	17	17	17	17
		wine sample no.	46	47	48	49	50	51	52	53	54	55	56	57	58	59	60
matrix		mean															
ethanol (%, ALC/VOL)		12.9										12.2	13.2	12.4	13.4	13.4	13.7
pH		3.4										3.18	3.6	3.1	3.5	3.0	3.4
odorant	OTC (µg/kg) in water	mean concentration (µg/L)															
ethyl acetate	5	69100											69786.18	36511.28	38453.62	41562.79	42578.02
acetaldehyde	16	49100															
butane-2,3-dione	1.0	1400															
ethyl hexanoate	1.2	1570			1168	1137	1330	1248	1250	1042	1155	994	250.35	330.38	303.32	377.32	318.78
ethyl 3-methylbutanoate	0.023	27.5			14	21	22	19	24	20	12	6.17					
ethyl 2-methylpropanoate	0.089	93.5			69	96	105	85	113	97	70	28.6					
3-methylbutan-1-ol	220	172000			201000	219000	238000	229000	259000	251000	211000	>10,000	193538.98	446662.77	271292.73	272105.44	238934.03
3-methylbutyl acetate	7.2	3650	1547	999	2630	2009	1919	1556	3690	1214	2704	10300	289.57	678.73	988.98	508.50	602.59
ethyl butanoate	0.76	374	378	680	337	441	487	462	451	359	530	346	460.06	485.20	522.48	530.97	551.41
ethyl 2-methylbutanoate	0.13	42.7										1.79					
ethyl octanoate	8.7	2460	1265	790	1697	1695	2505	1950	1650	1629	1578	617					
3-methylbutanal	0.50	119										13.6					
2-phenylethan-1-ol	140	28700	46000	19000	23000	23000	28000	38000	23000	18000	18000	18500	177482.08	64313.19	28524.58	26060.12	15604.60
2-methylpropanal	0.49	36.5															
2-methylbutan-1-ol	1200	70100															
dimethyl sulfide	0.30	14.1															
acetic acid	5600	219000															
3-(methylsulfanyl)propan-1-ol	36	1360			534	529	607	567	728	563	632						
3-(methylsulfanyl)propanal	0.43	14.6															
ethyl propanoate	10	295										202					
octanoic acid	190	5580											400.90	790.77	770.65	698.13	544.67
3-hydroxybutan-2-one	590	16600										2.98					
2-methylbutanal	1.5	40.2															
phenylacetic acid	68	452															
ethyl decanoate	122	741	227	78	581	574	608	497	450	416	435	180	44.48		147.35	74.33	71.64
decanoic acid	500	2460											127.06	227.66	191.73	189.73	162.32
hexan-1-ol	590	2710	1732	2872	2533	2506	2506	2363	1524	1723	2100	3250	1176.30	2144.75	1666.48	2681.69	1799.98
phenylacetaldehyde	5.2	21.5															
2-phenylethyl acetate	360	682										361	22.10	48.07	28.69	31.87	25.55
2-methylpropan-1-ol	19000	33000			15000	16000	21000	18000	19000	27000	16000	1020	20180.99	26707.39	16374.69	17187.89	21106.17
3-methylbutanoic acid	490	814											843.87	2158.70	929.77	1883.52	1002.61
2-methylpropyl acetate	66	101			81	60	94	57	84	64	85	87.6					
hexanoic acid	4800	4060											871.45	1271.76	1024.33	1196.27	1005.07
benzaldehyde	150	108										3.84	137.07				
butan-1-ol	1900	1120										251	5715.46	5518.39	4750.73	5646.75	6164.33
butanoic acid	2400	1180															
octan-1-ol	110	44.7										7.38	231.36	121.98		333.23	221.66
ethyl 2-phenylacetate	155.55	53.8										1.90					
2-methylbutanoic acid	3100	545															
ethyl dodecanoate	3500	269										3.50			8.00		
propanoic acid	20000	1490															
2-methylpropanoic acid	60000	2180											843.87	3588.63	5435.96		5201.42

		reference no.	18	18	18	18	18	18	19	19	19	19	19	20	20	20	20
		wine sample no.	61	62	63	64	65	66	67	68	69	70	71	72	73	74	75
matrix		mean															
ethanol (%, ALC/VOL)		12.9	14.3	13.9	14.5	14.2	13.9	14.5	13.50	11.25	11.50	11.85	12.27	12.0	11.0	12.2	13.4
pH		3.4	3.67	3.56	3.91	3.51	3.35	3.69	3.06	2.83	3.55	3.20	3.27	3.1	3.1	3.6	3.5
odorant	OTC (µg/kg) in water	mean concentration (µg/L)															
ethyl acetate	5	69100												40599.4	83063.5	40698.7	116484.0
acetaldehyde	16	49100															
butane-2,3-dione	1.0	1400							582	637	713	690	583				
ethyl hexanoate	1.2	1570	427	526	336	587	309	331	327	323	410	407	280	322.3	12237.4	518.4	1006.6
ethyl 3-methylbutanoate	0.023	27.5							8.36	12.35	2.70	5.46	6.63				
ethyl 2-methylpropanoate	0.089	93.5															
3-methylbutan-1-ol	220	172000	169570	169948	132191	133225	104536	141672	100710	100347	87927	93130	99320	49988.3	57452.2	36694.4	49266.5
3-methylbutyl acetate	7.2	3650	462	605	265	410	248	250	637	163	1130	917	210	939.0	16288.3	1666.4	2375.4
ethyl butanoate	0.76	374							160	120	193	153	137		123.2		
ethyl 2-methylbutanoate	0.13	42.7							3.36	5.57	1.06	1.68	3.06				
ethyl octanoate	8.7	2460	495	500	330	270	238	252	370	397	473	487	270	7608.9	8938.6	141.4	2260.4
3-methylbutanal	0.50	119															
2-phenylethan-1-ol	140	28700	52910	49544	30315	53576	29182	28644	22410	51950	14663	13460	37440	12140.8	20486.1	26253.8	99956.8
2-methylpropanal	0.49	36.5															
2-methylbutan-1-ol	1200	70100															
dimethyl sulfide	0.30	14.1															
acetic acid	5600	219000	229	223	176	446	399	560						25325.5	86927.4	33155.9	28340.6
3-(methylsulfanyl)propan-1-ol	36	1360							111	447	260	172	294	1487.0	2123.4	1862.2	3032.6
3-(methylsulfanyl)propanal	0.43	14.6															
ethyl propanoate	10	295															
octanoic acid	190	5580	571	566	419	290	166	237	2737	2550	3850	3293	2913	216.2	3614.8	727.7	1281.8
3-hydroxybutan-2-one	590	16600							2233	2883	4987	1263	1170				
2-methylbutanal	1.5	40.2															
phenylacetic acid	68	452															
ethyl decanoate	122	741	44	48	50	39	43	42	621	372	802	842	688	28.8	1504.0	58.3	545.9
decanoic acid	500	2460							713	977	1103	1057	703		1334.2	91.7	269.2
hexan-1-ol	590	2710	2534	2389	1583	2745	3611	2720	370	340	370	290	460	2516.2	3828.8	819.9	3608.9
phenylacetaldehyde	5.2	21.5														4.4	26.6
2-phenylethyl acetate	360	682	19	18	13	0.84	10	11	178	146	136	121	49	5.8	118.6	9.3	300.9
2-methylpropan-1-ol	19000	33000	567	516	438	545	491	642	11087	8033	9673	9993	8657	56007.6	15289.5	38885.4	52203.0
3-methylbutanoic acid	490	814	909	844	691	719	678	851	62	99	60	74	67				
2-methylpropyl acetate	66	101															
hexanoic acid	4800	4060	2948	2065	1206	1170	763	863	4467	3323	5520	3463	3850	445.4	3891.4	1175.0	2124.7
benzaldehyde	150	108	97	113	96	161	463	355						1.7		4.2	0.4
butan-1-ol	1900	1120	44	357	414	342	389	405	497	273	543	467	380	791.2	966.8	1012.2	897.6
butanoic acid	2400	1180	751	856	771	512	532	557	923	760	830	813	717				
octan-1-ol	110	44.7	15	15	22	402	12	9.1							19.4	10.9	5.2
ethyl 2-phenylacetate	155.55	53.8															
2-methylbutanoic acid	3100	545							84	125	80	99	133				
ethyl dodecanoate	3500	269	10	6.4	9.4	37	26	18							2238.1	168.0	
propanoic acid	20000	1490															37.3
2-methylpropanoic acid	60000	2180	775	1043	1170	1265	1740	2080	687	453	473	520	457		22.3		

		reference no.	20	20	20	20	21	21	21	22	23	24	25	26	26	26	26
		wine sample no.	76	77	78	79	80	81	82	83	84	85	86	87	88	89	90
matrix		mean															
ethanol (%, ALC/VOL)		12.9	11.0	10.8	11.0	11.5				12.5		10.35	13		14.0	14.1	14.0
pH		3.4	3.0	3.3	3.6	3.5				3.26		3.14			3.28	3.26	3.28
odorant	OTC (µg/kg) in water	mean concentration (µg/L)															
ethyl acetate	5	69100	28219.6	17232.2	74474.5	80951.2	ND		67182			4580	84835	61217.8	82570.1	62768.1	80324.0
acetaldehyde	16	49100									10158						
butane-2,3-dione	1.0	1400															
ethyl hexanoate	1.2	1570	191.6	1963.2	696.1	702.3	121	154	169	569.47	120	1140	816	1233.5	1046.9	1378.2	1453.0
ethyl 3-methylbutanoate	0.023	27.5					41.9	37.5	25.6		43.5		5.2	4.2	2.4	3.7	3.3
ethyl 2-methylpropanoate	0.089	93.5					153	146	114		202		33	10.7	9.1	6.5	8.1
3-methylbutan-1-ol	220	172000	50458.5	82302.5	90514.3	42856.2	169545	173274	244047	70512.29		14200	207775	134598.8	93584.2	125661.9	99056.8
3-methylbutyl acetate	7.2	3650	1005.0	3551.3	160.3	137.1	200	215	290	486.69	190	1090	2500	5267.5	6262.7	4886.7	7979.3
ethyl butanoate	0.76	374		342.2			135	154	152	136.42	140		603	521.1	408.9	526.5	492.9
ethyl 2-methylbutanoate	0.13	42.7					25.4	20.8	13.7		28.1		4.5	1.5	1.5	1.1	1.6
ethyl octanoate	8.7	2460	3947.2	686.6	2052.1	1082.5	92.5	119	103	625.26	90.0	2940	699	1594.1	1441.9	1500.3	2981.7
3-methylbutanal	0.50	119															
2-phenylethan-1-ol	140	28700	20879.2	19238.8	57925.3	62708.8	38529	38987	48203	16951.20		1790	13974	12648.2	15362.5	14170.1	12527.5
2-methylpropanal	0.49	36.5															
2-methylbutan-1-ol	1200	70100												20311.0	18255.8	17174.6	21381.7
dimethyl sulfide	0.30	14.1					59.7	28.8	20.7		57.4						
acetic acid	5600	219000	19955.9	25461.5	55749.9	89155.9	451300		323038		385000		30000				
3-(methylsulfanyl)propan-1-ol	36	1360	1641.6	2092.6	2247.6	3136.6	339	298	1200	2903.20			1256				
3-(methylsulfanyl)propanal	0.43	14.6					8.62	1.22	1.91		8.43						
ethyl propanoate	10	295					142	186	260		140			87.4	113.1	106.7	93.3
octanoic acid	190	5580	151.7	1201.3	899.1	782.6	429	445	1439	5746.37	440		4981	7447.6	6831.7	8474.8	10495.1
3-hydroxybutan-2-one	590	16600					6025	9860	12886				763				
2-methylbutanal	1.5	40.2															
phenylacetic acid	68	452					38.8	43.9					265				
ethyl decanoate	122	741	39.1	93.9	291.6	57.4	36.7	58.0	72.2	273.65	60.6	1720	423				
decanoic acid	500	2460		59.7	26.7		190	207	131	2252.64	160	1100	737	3197.9	3802.5	3155.9	4860.5
hexan-1-ol	590	2710	797.9	1001.5	2703.9	1485.3	1012	1036	1940	1642.14	1000	2890	1391	1456.3	1010.9	1595.0	1130.0
phenylacetaldehyde	5.2	21.5	4.9			26.0	53.5	6.81	9.79		55.0						
2-phenylethyl acetate	360	682	7.0	11.1		9.1	32.3	28.0	475	216.55		423	89	359.0	835.2	459.9	589.8
2-methylpropan-1-ol	19000	33000	69451.8	20534.5	39045.6	976.8	39136	41011	51279			4620	56454	15790.4	12066.1	12110.6	12864.9
3-methylbutanoic acid	490	814					142	240	495	622.45	139		508	522.6	281.9	314.0	345.7
2-methylpropyl acetate	66	101					44.6	36.1	64.0				73	69.8	125.8	61.0	118.9
hexanoic acid	4800	4060	309.7	1328.0	1376.9	1137.9	1068	1172	2232	2823.36	1090	401	2505	4377.2	3921.5	5575.3	6040.8
benzaldehyde	150	108	1.6	94.6	3.6	79.8			10.1	138.18							
butan-1-ol	1900	1120	1083.4	853.9	1188.0	1057.9	755	862	1243	186.98		21					
butanoic acid	2400	1180					506	727	1542	782.90	580		1342				
octan-1-ol	110	44.7		3.7	62.7	18.4						65					
ethyl 2-phenylacetate	155.55	53.8															
2-methylbutanoic acid	3100	545					219	247			208			308.6	292.5	210.4	283.1
ethyl dodecanoate	3500	269	85.1	1025.5	259.7							181					
propanoic acid	20000	1490				14.1											
2-methylpropanoic acid	60000	2180		10.8			870	1093	1325		1695	60	936	790.7	764.8	637.1	613.2

		reference no.	26	26	26	27	27	27	28	28	28	28	28	28	28	29	30
		wine sample no.	91	92	93	94	95	96	97	98	99	100	101	102	103	104	105
matrix		mean															
ethanol (%, ALC/VOL)		12.9	14.0	14.0	13.9	12.9	12.9	12.9	13.1	13.2	13.4	13.7	13.6	13.7	13.7	13.98	
pH		3.4	3.27	3.28	3.27	3.27	3.28	3.33	3.32	3.42	3.46	3.50	3.47	3.50	3.40	3.93	
odorant	OTC (µg/kg) in water	mean concentration (µg/L)															
ethyl acetate	5	69100	77787.0	49303.9	64279.2	81000	91000	94000	37700	39000	49000	60000	64000	57000	52000	41100	30712
acetaldehyde	16	49100							79000	90000	82000	86300	86320	82900	88000	60800	
butane-2,3-dione	1.0	1400															
ethyl hexanoate	1.2	1570	1662.4	1251.2	1114.5	1600	1700	1400	1076	928	1282	1144	1145	1040	1096	751	827
ethyl 3-methylbutanoate	0.023	27.5	3.2	3.7	3.8	3.3	5.7	5.5								1.6	11
ethyl 2-methylpropanoate	0.089	93.5	17.8	16.0	13.0	19	11	17									150
3-methylbutan-1-ol	220	172000	137185.4	94932.8	108961.1	12	12	10	224000	246000	196000	204000	190000	195000	197000	156000	102417
3-methylbutyl acetate	7.2	3650	5628.2	3255.1	3820.3	4500	8600	9300	1673	1769	2000	1698	1597	1507	1847	2163	2357
ethyl butanoate	0.76	374	715.8	384.5	438.0	670	610	520	584	474	697	586	609	538	607	403	2327
ethyl 2-methylbutanoate	0.13	42.7	1.7	1.8	1.8	1.5	0.98	1.9								2.1	21
ethyl octanoate	8.7	2460	1880.6	3016.4	1621.0	1700	1900	2100	1790	1556	2232	1932	2032	1955	1166	941	689
3-methylbutanal	0.50	119															
2-phenylethan-1-ol	140	28700	14247.7	13785.6	11929.4	12000	13000	12000	28000	30000	25000	22300	22000	23000	19800	12700	34004
2-methylpropanal	0.49	36.5															
2-methylbutan-1-ol	1200	70100	24624.4	13950.1	23073.1	22000	15000	25000									
dimethyl sulfide	0.30	14.1															
acetic acid	5600	219000															
3-(methylsulfanyl)propan-1-ol	36	1360															
3-(methylsulfanyl)propanal	0.43	14.6															
ethyl propanoate	10	295	60.2	44.9	99.3	65	110	100								9.6	1604
octanoic acid	190	5580	9457.9	8315.1	6870.4	8500	10000	10000	11302	14560	13512	14453	15544	16129	13710	4005	2486
3-hydroxybutan-2-one	590	16600							26000	31000	34000	38000	38000	38000	34000		
2-methylbutanal	1.5	40.2															
phenylacetic acid	68	452															
ethyl decanoate	122	741				890	1100	1400	613	547	729	646	876	861	344	583	333
decanoic acid	500	2460	3991.7	3982.1	3381.7	3700	4000	5000	3311	4168	3327	3977	5455	5987	2567	944	551
hexan-1-ol	590	2710	1310.4	1687.9	1583.0	1200	1200	950	692	618	754	716	780	762.7	714.4	1361	97
phenylacetaldehyde	5.2	21.5															
2-phenylethyl acetate	360	682	553.5	460.2	292.0	340	540	510	103	128	97	87.3	71	71	84	1679	495
2-methylpropan-1-ol	19000	33000	22386.2	12737.4	13624.1	19000	13000	14000	28400	27500	24200	25000	23400	24040	24500	33800	23204
3-methylbutanoic acid	490	814	388.8	440.5	440.5	750	460	550									
2-methylpropyl acetate	66	101	128.9	55.8	54.9	100	120	190								91.4	111
hexanoic acid	4800	4060	6468.4	5289.4	4207.7	7600	7600	6600	11	10	3		10.2	8.3	10	3792	
benzaldehyde	150	108							93.5	116	122	126	118	119	117.5	32.6	1
butan-1-ol	1900	1120				800	1000	1700								900	
butanoic acid	2400	1180				1500	1300	1500	48		43		16	45	1.7	858	
octan-1-ol	110	44.7														5.8	16
ethyl 2-phenylacetate	155.55	53.8														3.3	
2-methylbutanoic acid	3100	545	351.8	337.2	368.8	400	240	380									
ethyl dodecanoate	3500	269				260	330	1200								219	
propanoic acid	20000	1490				1300	1400	910									
2-methylpropanoic acid	60000	2180	1275.3	1041.4	926.6	1300	650	1000									

		reference no.	31	31	32	33	34	34	34	34	35	35	35	35	35	35	35
		wine sample no.	106	107	108	109	110	111	112	113	114	115	116	117	118	119	120
matrix		mean															
ethanol (%, ALC/VOL)		12.9	13.9	13.8													
pH		3.4	3.68	3.73													
odorant	OTC (µg/kg) in water	mean concentration (µg/L)															
ethyl acetate	5	69100									3940.6	47127.4	14468.1	2634.1	18319.6	20596.8	2834.9
acetaldehyde	16	49100			2520												
butane-2,3-dione	1.0	1400			190												
ethyl hexanoate	1.2	1570	859.8	865.9	785	461	650	540	630	300	141.34	113.92	59.22	134.85	76.59	86.45	88.82
ethyl 3-methylbutanoate	0.023	27.5			15	24.1					22.62	23.74	35.88	22.37	24.66	22.74	27.24
ethyl 2-methylpropanoate	0.089	93.5			51	189					73.98	33.24	70.59	48.63	78.89	52.86	58.97
3-methylbutan-1-ol	220	172000	196475	231804	186650		164300	158000	159500	152300	658.46	989.66	410.32	84.15	621.64	427.57	613.88
3-methylbutyl acetate	7.2	3650	1350.32	1576.61	1605		2100	1400	1000	5000	39.99	51.11	61.47	87.55	80.94	42.45	66.87
ethyl butanoate	0.76	374	282.99	395.46	343	137	440	390	440	310	52.66	31.48	19.98	33.28	16.57	19.85	26.52
ethyl 2-methylbutanoate	0.13	42.7			9.8								40.40				
ethyl octanoate	8.7	2460	1341.7	1109.7	627	1025	1410	1480	1210	630	517.80	685.40	932.27	611.28	449.59	181.23	193.89
3-methylbutanal	0.50	119			2.1												
2-phenylethan-1-ol	140	28700	15253	11398	31183	16102	8500	7800	9100	3700	6203.49	6230.03	6979.33	13755.7	4898.57	5592.11	8292.00
2-methylpropanal	0.49	36.5															
2-methylbutan-1-ol	1200	70100															
dimethyl sulfide	0.30	14.1															
acetic acid	5600	219000															
3-(methylsulfanyl)propan-1-ol	36	1360	31.91	38.64													
3-(methylsulfanyl)propanal	0.43	14.6			0.99												
ethyl propanoate	10	295															
octanoic acid	190	5580	314.05	210.51			6400	6100	6900	3200	3807.39	6421.96	1822.28	4120.54	3418.59	2168.15	4197.20
3-hydroxybutan-2-one	590	16600	1191.7	2323.7			3100	2100	2600	1800							
2-methylbutanal	1.5	40.2															
phenylacetic acid	68	452															
ethyl decanoate	122	741	1091.6	732.1			580	630	630	300	90.74	418.01	504.42	445.71	304.19	82.62	104.78
decanoic acid	500	2460	211.69	206.56			2100	2000	2100	900	502.61	580.00	392.18	527.14	429.10	276.97	471.82
hexan-1-ol	590	2710	2035.3	1547.1		727	1300	1100	1400	700	883.41		190.75	427.96	693.24	263.11	1050.6
phenylacetaldehyde	5.2	21.5	2.55	2.51	3.9												
2-phenylethyl acetate	360	682	33.11	32.62	409	4.79	100	90	70	20							
2-methylpropan-1-ol	19000	33000	167819	283322	25083		20200	19200	22300	17900							
3-methylbutanoic acid	490	814	731.62	405.92	522		750	730	740	400		604.29	643.09	719.43	590.55	391.90	682.62
2-methylpropyl acetate	66	101			102												
hexanoic acid	4800	4060	638.7	416.39			4700	4200	4700	2200	2005.65	1036.98	554.53	2255.54	1269.00	687.95	1218.34
benzaldehyde	150	108	1.11	2.43			20	30	30	10							
butan-1-ol	1900	1120	1673.8	2220.2			1300	1300	1300	900				1171.2			
butanoic acid	2400	1180			1460		2880	2750	2900	1620							
octan-1-ol	110	44.7	11.51	7.22													
ethyl 2-phenylacetate	155.55	53.8	1.62	0.66		7.14											
2-methylbutanoic acid	3100	545															
ethyl dodecanoate	3500	269	885.7	477.3													
propanoic acid	20000	1490	1360.6	1211.7													
2-methylpropanoic acid	60000	2180	1671.84	1359.14			1010	930	930	560	2207.28	15542.6	11038.8	10495.4	24711.2		3577.02

		reference no.	35	35	35	35	35	36	37	37	38	38	39	40	40	40	41
		wine sample no.	121	122	123	124	125	126	127	128	129	130	131	132	133	134	135
matrix		mean															
ethanol (%, ALC/VOL)		12.9						10.23	12.2	13.4	13.7	12.2	13.2				13.5
pH		3.4						3.29	3.24	3.29			4.42				3.45
odorant	OTC (µg/kg) in water	mean concentration (µg/L)															
ethyl acetate	5	69100	3221.43	12725.9	18255	21274.6	32738.7	21000	25920	21510			50000	71000	23000	33000	56000
acetaldehyde	16	49100						87000					6000	50000	27000	3000	60000
butane-2,3-dione	1.0	1400															
ethyl hexanoate	1.2	1570	83.64	37.69	360.51	42.27	45.14		395.57	344.74	5.41	11.6	110	460	420	260	520
ethyl 3-methylbutanoate	0.023	27.5	28.19	21.33	20.15	17.68	25.63		18.18	9.34	0.08	0.05					
ethyl 2-methylpropanoate	0.089	93.5	37.29	36.26	23.06	22.90	67.10	3	3.27	2.55							
3-methylbutan-1-ol	220	172000	1139.43	397.39	350.11	1213.94	694.58		206470	249210	9.05	6.68	57000	211000	245000	217000	193000
3-methylbutyl acetate	7.2	3650		38.67	115.56	86.49	52.72	5000	2750	2300	0.51	0.21	600	200	400	1000	900
ethyl butanoate	0.76	374	23.63	13.39	36.68	18.04	14.36	2210	418.99	252.69	2.69	4.12	90	120		70	150
ethyl 2-methylbutanoate	0.13	42.7							6.71	6.17	0.47	0.25					
ethyl octanoate	8.7	2460	404.72	234.32	2036.60	266.69	376.58	2817	560.40	231.06	7.32	32.3	150	760	410	380	830
3-methylbutanal	0.50	119															
2-phenylethan-1-ol	140	28700	7838.43	5675.31	5506.71	6238.15	6201.87	49000	13680	34130	240	50	25000	28000	40000	32000	26000
2-methylpropanal	0.49	36.5															
2-methylbutan-1-ol	1200	70100											215000	51000	57000	49000	44000
dimethyl sulfide	0.30	14.1															
acetic acid	5600	219000															
3-(methylsulfanyl)propan-1-ol	36	1360									103	38.2	1230	370	360	350	220
3-(methylsulfanyl)propanal	0.43	14.6															
ethyl propanoate	10	295	1.72					160	2.23	3.16							
octanoic acid	190	5580	5756.39	2662.45	176.37	5869.04	3681.95	12400	5290	5030	9.01	35.2	1550	2660	1890	2430	3360
3-hydroxybutan-2-one	590	16600						19000					12000	11000	5000		56000
2-methylbutanal	1.5	40.2															
phenylacetic acid	68	452															
ethyl decanoate	122	741	448.32	329.65	1380.10	112.35	715.19	650	174.10	254.05	2.64	12.4	30	150	200	10	90
decanoic acid	500	2460	581.14	372.60	854.76	599.22	474.51	960	1240	1600	2.16	15.7	310	650	750	680	510
hexan-1-ol	590	2710	1092.52	782.53	294.74	176.58	778.49	1200	1150	860			1700	1200	1300	1300	800
phenylacetaldehyde	5.2	21.5															
2-phenylethyl acetate	360	682						750	1070	2350	0.05	0.04	250	50	150	80	110
2-methylpropan-1-ol	19000	33000						20000	23150	29400			43000	43000	31000	26000	53000
3-methylbutanoic acid	490	814	707.32	387.07	465.99	572.32	527.95						1040	1290	2620	200	610
2-methylpropyl acetate	66	101							60.85	41.40							
hexanoic acid	4800	4060	957.58	518.65	6642.60	691.88	387.49	140	5390	3710			2060	2960	2510	2130	3570
benzaldehyde	150	108						8	5.82	3.03							
butan-1-ol	1900	1120	746.78		940.68												
butanoic acid	2400	1180											1410	1840	1380	890	1980
octan-1-ol	110	44.7							23.31	18.58							
ethyl 2-phenylacetate	155.55	53.8															
2-methylbutanoic acid	3100	545															
ethyl dodecanoate	3500	269						0.8	10.79	5.71	0.12	0.3					
propanoic acid	20000	1490									220	180					
2-methylpropanoic acid	60000	2180	20143.21	16354.0	6841.64	12660.9	8427.95						1770	2840	4030	2350	2000

		reference no.	41	41	42	42	42	43	44	45	46	46	46	47	48	48	48
		wine sample no.	136	137	138	139	140	141	142	143	144	145	146	147	148	149	150
matrix		mean															
ethanol (%, ALC/VOL)		12.9	14.7	13.6	11.4	11.9	12.0			12.3	11.6	11.3	11.8	14.5	14.36	14.79	14.01
pH		3.4	3.40	3.48	3.15	3.18	3.21								3.70	3.69	3.72
odorant	OTC (µg/kg) in water	mean concentration (µg/L)															
ethyl acetate	5	69100	27000	34000					87300	14300				60940			
acetaldehyde	16	49100	33000	33000					6491					13770			
butane-2,3-dione	1.0	1400							990								
ethyl hexanoate	1.2	1570	480	280	208.5	284.2	303.2		357	790				353	760	256	441
ethyl 3-methylbutanoate	0.023	27.5			12.9	14.9	16.9		51.3					6.46	4.4	6.6	8.4
ethyl 2-methylpropanoate	0.089	93.5			26.6	30.9	30.6		141					50.1			
3-methylbutan-1-ol	220	172000	275000	226000				12448.25	245000	118000	203000	213100	195100				
3-methylbutyl acetate	7.2	3650	1200	1700	816.4	1060.9	1024.0		333	290				480	995	562	818
ethyl butanoate	0.76	374		100	191.0	226.2	281.1		120	1200	200	200	300	214			
ethyl 2-methylbutanoate	0.13	42.7			4.4	5.5	6.2		27.4		17.8	11.9	3.8	6.69	4.1	5.7	7.6
ethyl octanoate	8.7	2460	650	470	80.7	120.8	115.9		233	1030				251	515	231	350
3-methylbutanal	0.50	119															
2-phenylethan-1-ol	140	28700	58000	38000	15100	17200	18400	37026.81	40900	17100	32800	35800	14200	18155	40983	41161	50821
2-methylpropanal	0.49	36.5							45.5								
2-methylbutan-1-ol	1200	70100	63000	52000													
dimethyl sulfide	0.30	14.1							20.0					33.8			
acetic acid	5600	219000							451000		404000	443800	346900		144	194	127
3-(methylsulfanyl)propan-1-ol	36	1360	180	350													
3-(methylsulfanyl)propanal	0.43	14.6									1.7	2.2	1.3				
ethyl propanoate	10	295							220					197.3			
octanoic acid	190	5580	2520	3090	4800	5200	5600			6650				1403	1801	898	1164
3-hydroxybutan-2-one	590	16600	3000	3000					22300								
2-methylbutanal	1.5	40.2															
phenylacetic acid	68	452									8.3	9.9	11.9				
ethyl decanoate	122	741	210	160					79.2	62				120	24	11	16
decanoic acid	500	2460	720	1060	1300	1400	1600			305				516	382	236	323
hexan-1-ol	590	2710	1100	1200	910.5	980.3	963.6	627.37		5910				934	1611	1340	1591
phenylacetaldehyde	5.2	21.5															
2-phenylethyl acetate	360	682	520	180	915.6	1048.6	1053.1		27.3	145				40.8	30	38	37
2-methylpropan-1-ol	19000	33000	35000	33000					35400	33600				70830	622	670	392
3-methylbutanoic acid	490	814	2110	1480											1147	1049	1530
2-methylpropyl acetate	66	101			18.6	29.5	29.2		8.10	64				63.1			
hexanoic acid	4800	4060	2920	2430	3500	4500	4200		2290	1170	900	1700	2100	1287	1916	926	1291
benzaldehyde	150	108							11.1								
butan-1-ol	1900	1120							718	1150					542	349	258
butanoic acid	2400	1180	1540	1180					968		200	200	200	765	1054	586	790
octan-1-ol	110	44.7								75					18	13	15
ethyl 2-phenylacetate	155.55	53.8								125				2.42			
2-methylbutanoic acid	3100	545															
ethyl dodecanoate	3500	269								1					5.5	6.5	3.9
propanoic acid	20000	1490												2488			
2-methylpropanoic acid	60000	2180	2990	2300						20	1300	1300	1700	2371	997	1316	1427

		reference no.	49	50	50	51	51	52	52	52	52	52	52	53	54	55	56
		wine sample no.	151	152	153	154	155	156	157	158	159	160	161	162	163	164	165
matrix		mean															
ethanol (%, ALC/VOL)		12.9	14.32	14.2	12.4	12.1	12.9								13.28	15.79	13.16
pH		3.4	3.55	3.84	3.48	3.6	3.2								3.60	3.96	3.47
odorant	OTC (µg/kg) in water	mean concentration (µg/L)															
ethyl acetate	5	69100	94170											15608.09		29450	
acetaldehyde	16	49100	9700														
butane-2,3-dione	1.0	1400						60.9	74.7	672	711	5352	8082				
ethyl hexanoate	1.2	1570	310	462	263	589.1	547.6							385.96		834.8	770
ethyl 3-methylbutanoate	0.023	27.5	26.3											5.31			
ethyl 2-methylpropanoate	0.089	93.5	266											30.08			
3-methylbutan-1-ol	220	172000	282570												276000	276130	81500
3-methylbutyl acetate	7.2	3650	260	503	410	1869.8	1338.7							1536.07		183.3	5560
ethyl butanoate	0.76	374	200			126.1	110.2							15189.87		381.8	290
ethyl 2-methylbutanoate	0.13	42.7	20.1														
ethyl octanoate	8.7	2460	270	462	270	1048.7	836.5							75450.87		1776.7	1257
3-methylbutanal	0.50	119						6.26	7.02	16.8	27.3	5.75	9.30		26.20		
2-phenylethan-1-ol	140	28700	46760	52910	53576	37197.0	36443.2							356679.48	41500	53750	14200
2-methylpropanal	0.49	36.5						6.87	11.7	43.5	47.5	7.87	9.37		44.80		
2-methylbutan-1-ol	1200	70100															
dimethyl sulfide	0.30	14.1													11.60		
acetic acid	5600	219000	353430	229	446	270000	420000										
3-(methylsulfanyl)propan-1-ol	36	1360	1710			1480.4	878.6								1720		350
3-(methylsulfanyl)propanal	0.43	14.6						4.80	5.11	15.2	22.4	5.4	8.17		2.49		
ethyl propanoate	10	295	190											10450.44		10.8	
octanoic acid	190	5580	1870	2948	1170	4328.0	3707.5							160320.82		2530	2114
3-hydroxybutan-2-one	590	16600	20020														
2-methylbutanal	1.5	40.2						2.53	3.67	14.0	15.7	3.23	3.73		11.80		
phenylacetic acid	68	452															
ethyl decanoate	122	741	260	44	39	269.1	264.3							1950.32		826.7	
decanoic acid	500	2460	510	571	290	1200.9	1183.6							180.10		603	765
hexan-1-ol	590	2710	1840	2534	2745	595.6	1575.9							54.15	1960	2010	1500
phenylacetaldehyde	5.2	21.5				2.7	2.6	2.06	2.20	12.3	16.1	4.31	5.07		14.40	16.07	
2-phenylethyl acetate	360	682		19	0.84	357.5	265.9										280
2-methylpropan-1-ol	19000	33000	58203	567	545										53600	7290	
3-methylbutanoic acid	490	814	1830	908	676	237.5	212.2										
2-methylpropyl acetate	66	101	68.7													4773.1	60
hexanoic acid	4800	4060	1890	2622	2130	1958.4	1792.6							49.59		1407	5445
benzaldehyde	150	108	2.93			12.7	10.3	6.10	5.64	5.53	6.07	9.79	8.30		1.21	5.20	
butan-1-ol	1900	1120	1320	44	342										1280		
butanoic acid	2400	1180	1380	751	512	336.6	319.8										
octan-1-ol	110	44.7		15	4.2											372.34	
ethyl 2-phenylacetate	155.55	53.8				2.7	3.1							3420.04		38.6	
2-methylbutanoic acid	3100	545															
ethyl dodecanoate	3500	269		10	37											425.7	
propanoic acid	20000	1490															
2-methylpropanoic acid	60000	2180	3040	775	1265												

		reference no.	57	57	57	57	57	57	58	59	60	61	61	62	63	64	64
		wine sample no.	166	167	168	169	170	171	172	173	174	175	176	177	178	179	180
matrix		mean															
ethanol (%, ALC/VOL)		12.9							13.1			14.2	14.3	14.3			
pH		3.4							3.1	3.2		3.53	3.53				
odorant	OTC (µg/kg) in water	mean concentration (µg/L)															
ethyl acetate	5	69100	7761	6954	5703	6635	6245	8448	85430	82390		58440	50591		5000.0	22803	31500
acetaldehyde	16	49100								32275		49326	52953	11000	2962.7		
butane-2,3-dione	1.0	1400												690	81.8	1649	2501
ethyl hexanoate	1.2	1570	36	45	59	42	30	39	410	1050	489	541	413		783.3	227	76
ethyl 3-methylbutanoate	0.023	27.5													14.1	20	25
ethyl 2-methylpropanoate	0.089	93.5							36	5		179	226		220.1	254	168
3-methylbutan-1-ol	220	172000	5789	7167	7056	6723	6609	9292	120050	154420		190722	195728		149528.3	235696	257993
3-methylbutyl acetate	7.2	3650	93	109	59	118	107	220	1112	3915	16	571	571	2130	1090.9	141	156
ethyl butanoate	0.76	374	200	275	299	299	361	268	184	376		154	132		374.8	170	75
ethyl 2-methylbutanoate	0.13	42.7										21	22		11.2	13	17
ethyl octanoate	8.7	2460	35	48	59	45	30	36	764	1530	360	525	501		773.3	102	40
3-methylbutanal	0.50	119															
2-phenylethan-1-ol	140	28700	906	1254	1242	1070	879	1658	36210	20620	13355	71879	403885	47800	22547.9	46207	96292
2-methylpropanal	0.49	36.5															
2-methylbutan-1-ol	1200	70100	1150	1170	1420	1181	1249	1763	27720	21040		73153	77041				
dimethyl sulfide	0.30	14.1										2.5	2			60	44
acetic acid	5600	219000							8000000								
3-(methylsulfanyl)propan-1-ol	36	1360									176	2900	3054	1720	252.1	1905	2468
3-(methylsulfanyl)propanal	0.43	14.6															
ethyl propanoate	10	295							23	47		169	202	70			
octanoic acid	190	5580							5850	11050	1557	834	848	2570	9766.8	2135	1035
3-hydroxybutan-2-one	590	16600								2887				2370	2992.1	30259	11304
2-methylbutanal	1.5	40.2															
phenylacetic acid	68	452													103.6	25.0	55.3
ethyl decanoate	122	741	28	31	34	25	22	24	306	372	75				544.4	15.0	4.1
decanoic acid	500	2460							1520	1770	326				16861.3	1355	92.8
hexan-1-ol	590	2710	1918	1949	2212	1899	1811	1603	540	811	1580	310	396	1820	824.3	1478	1463
phenylacetaldehyde	5.2	21.5									0.2				1.5	1.5	
2-phenylethyl acetate	360	682							465	344	32	102	155	130	213.8	16.0	36
2-methylpropan-1-ol	19000	33000	6666	9744	7400	12829	11825	15259	27450	18080				31500	20639.0	57893	47966
3-methylbutanoic acid	490	814									258	2797	2205		349.3	1651	1860
2-methylpropyl acetate	66	101										154	44		70.9	38	43
hexanoic acid	4800	4060							5430	13380	1546	2007	1379		9499.1	3328	2557
benzaldehyde	150	108									12						
butan-1-ol	1900	1120	49	46	56	33	101	47						690	2065.7	2396	2002
butanoic acid	2400	1180									735				995.9		440
octan-1-ol	110	44.7															
ethyl 2-phenylacetate	155.55	53.8									5.2						
2-methylbutanoic acid	3100	545										6527	2263		233.4	265	89.0
ethyl dodecanoate	3500	269	1	5	10	3	2	4									
propanoic acid	20000	1490													1621.1	2793	3020
2-methylpropanoic acid	60000	2180													410.6	2082	670

		reference no.	64	64	64	65	65	65	65	66	66	66	66	66	67	67	67
		wine sample no.	181	182	183	184	185	186	187	188	189	190	191	192	193	194	195
matrix		mean															
ethanol (%, ALC/VOL)		12.9															
pH		3.4															
odorant	OTC (µg/kg) in water	mean concentration (µg/L)															
ethyl acetate	5	69100	37267	66600	10440										67900	24900	46000
acetaldehyde	16	49100													45200	44500	14300
butane-2,3-dione	1.0	1400	237	690	1000												
ethyl hexanoate	1.2	1570	100	29	31										1200	500	1200
ethyl 3-methylbutanoate	0.023	27.5	25	20	22												
ethyl 2-methylpropanoate	0.089	93.5	315	98	30												
3-methylbutan-1-ol	220	172000	277139	112800	118800										87000	167200	86200
3-methylbutyl acetate	7.2	3650	221	120	132										5300	1700	2900
ethyl butanoate	0.76	374	94	69	70												
ethyl 2-methylbutanoate	0.13	42.7	21	32	9.2												
ethyl octanoate	8.7	2460	69	24	33										1100	300	1000
3-methylbutanal	0.50	119				6.52	3.37	12.2	31.3								
2-phenylethan-1-ol	140	28700	79220	60300	58900										20100	15800	20200
2-methylpropanal	0.49	36.5				4.96	1.93	11.8	44.2								
2-methylbutan-1-ol	1200	70100													17900	42500	21400
dimethyl sulfide	0.30	14.1	104	19	18												
acetic acid	5600	219000															
3-(methylsulfanyl)propan-1-ol	36	1360	1624	3750	3410										500	900	400
3-(methylsulfanyl)propanal	0.43	14.6				2.14	1.08	9.82	24.4	14.5	5.4	2.9	4.2	5.1			
ethyl propanoate	10	295															
octanoic acid	190	5580	546	1910	1700										6200	3000	5500
3-hydroxybutan-2-one	590	16600	16555	55900	18300												
2-methylbutanal	1.5	40.2				16.4	9.16	18.8	90.2								
phenylacetic acid	68	452	114	108	63.0												
ethyl decanoate	122	741	8.2	6.6	6.2												
decanoic acid	500	2460	359	2430	3010										2700	700	1700
hexan-1-ol	590	2710	1180	780	1190										267.8	547.6	481.6
phenylacetaldehyde	5.2	21.5	9.9			11.0	4.63	17.5	91.2	32	14	20	17	12			
2-phenylethyl acetate	360	682	41	29	21										200	10	100
2-methylpropan-1-ol	19000	33000	89670	28700	33300										14900	28900	15200
3-methylbutanoic acid	490	814	1770	1670	2180												
2-methylpropyl acetate	66	101	57	40	10												
hexanoic acid	4800	4060	1031	2730	2730										4900	2000	4400
benzaldehyde	150	108														6.6	
butan-1-ol	1900	1120	2502	1999	1900												
butanoic acid	2400	1180			1360										900	700	400
octan-1-ol	110	44.7															
ethyl 2-phenylacetate	155.55	53.8															
2-methylbutanoic acid	3100	545	234	225	198												
ethyl dodecanoate	3500	269															
propanoic acid	20000	1490	3112	8450	5970												
2-methylpropanoic acid	60000	2180	2037	3140	4260										800	1500	1200

		reference no.	67	68	69	70	71	71	71	71	71	71	71	71	71	71	71
		wine sample no.	196	197	198	199	200	201	202	203	204	205	206	207	208	209	210
matrix		mean															
ethanol (%, ALC/VOL)		12.9		12.0													
pH		3.4															
odorant	OTC (µg/kg) in water	mean concentration (µg/L)															
ethyl acetate	5	69100	20900	27700													
acetaldehyde	16	49100	19800	23400													
butane-2,3-dione	1.0	1400															
ethyl hexanoate	1.2	1570	700	350	730	240											
ethyl 3-methylbutanoate	0.023	27.5				5.7											
ethyl 2-methylpropanoate	0.089	93.5				281											
3-methylbutan-1-ol	220	172000	123700	143000		295757											
3-methylbutyl acetate	7.2	3650	3700	2630	3640	150	694	641	1180	1706	2471	1847	1107	1634	726	428	992
ethyl butanoate	0.76	374			810	160	991	696	880	872	987	880	1179	1352	796	785	771
ethyl 2-methylbutanoate	0.13	42.7				11.2											
ethyl octanoate	8.7	2460	800	350	860	130											
3-methylbutanal	0.50	119															
2-phenylethan-1-ol	140	28700	31000	4670	20730	36900											
2-methylpropanal	0.49	36.5															
2-methylbutan-1-ol	1200	70100	45400	54600													
dimethyl sulfide	0.30	14.1															
acetic acid	5600	219000															
3-(methylsulfanyl)propan-1-ol	36	1360	1400	1440	840												
3-(methylsulfanyl)propanal	0.43	14.6															
ethyl propanoate	10	295															
octanoic acid	190	5580	3400	2480	4900		24086	16847	17785	18362	22537	15604	13805	26283	20439	19268	17250
3-hydroxybutan-2-one	590	16600															
2-methylbutanal	1.5	40.2															
phenylacetic acid	68	452															
ethyl decanoate	122	741		180	60												
decanoic acid	500	2460	1000	550	1480		8638	6885	5395	5701	7853	4819	4472	8403	7909	6085	5540
hexan-1-ol	590	2710	607.8	230	1150												
phenylacetaldehyde	5.2	21.5		5.6		2.0	28	19	13	8	6	15	20	11	19	17	16
2-phenylethyl acetate	360	682	200	20	350												
2-methylpropan-1-ol	19000	33000	17600	23200		81260											
3-methylbutanoic acid	490	814		420	980	1170											
2-methylpropyl acetate	66	101				48											
hexanoic acid	4800	4060	2100	1460	3750												
benzaldehyde	150	108		16.4													
butan-1-ol	1900	1120															
butanoic acid	2400	1180	600	500	580	18080											
octan-1-ol	110	44.7															
ethyl 2-phenylacetate	155.55	53.8															
2-methylbutanoic acid	3100	545															
ethyl dodecanoate	3500	269															
propanoic acid	20000	1490															
2-methylpropanoic acid	60000	2180	1700	1910	2590												

		reference no.	71	71	71	71	71	71	71	71	71	72	73	73	73	73	73
		wine sample no.	211	212	213	214	215	216	217	218	219	220	221	222	223	224	225
matrix		mean															
ethanol (%, ALC/VOL)		12.9															
pH		3.4															
odorant	OTC (µg/kg) in water	mean concentration (µg/L)															
ethyl acetate	5	69100										30200					
acetaldehyde	16	49100										46500	114000	49300	36500	2440	49200
butane-2,3-dione	1.0	1400															
ethyl hexanoate	1.2	1570										350					
ethyl 3-methylbutanoate	0.023	27.5															
ethyl 2-methylpropanoate	0.089	93.5															
3-methylbutan-1-ol	220	172000										222400					
3-methylbutyl acetate	7.2	3650	1034	415	2614	932	570	926	1037	348	3237	3210	790	1020	310	330	1050
ethyl butanoate	0.76	374	842	807	839	793	821	864	271	362	482						
ethyl 2-methylbutanoate	0.13	42.7											3.9	11.9	22.5	18.4	3.9
ethyl octanoate	8.7	2460										440					
3-methylbutanal	0.50	119															
2-phenylethan-1-ol	140	28700										22886	75900	71000	55600	57700	70000
2-methylpropanal	0.49	36.5															
2-methylbutan-1-ol	1200	70100										64900					
dimethyl sulfide	0.30	14.1															
acetic acid	5600	219000															
3-(methylsulfanyl)propan-1-ol	36	1360										1930	8080	7810	6330	3490	13300
3-(methylsulfanyl)propanal	0.43	14.6															
ethyl propanoate	10	295															
octanoic acid	190	5580	19891	17574	18672	16796	18710	19947	6714	4648	9161	2790	2240	2350	1590	1380	2400
3-hydroxybutan-2-one	590	16600															
2-methylbutanal	1.5	40.2															
phenylacetic acid	68	452															
ethyl decanoate	122	741															
decanoic acid	500	2460	6773	6664	6911	6669	6802	5787	4449	4320	4191	650					
hexan-1-ol	590	2710										442	3540	3520	2690	1850	3960
phenylacetaldehyde	5.2	21.5	15	26	6	94	6	17	21	30	4			3.28	7.15	6.64	
2-phenylethyl acetate	360	682										10					
2-methylpropan-1-ol	19000	33000										31400					
3-methylbutanoic acid	490	814											2020	2050	1350	1410	3700
2-methylpropyl acetate	66	101															
hexanoic acid	4800	4060										1780	3020	2650	1990	2300	2700
benzaldehyde	150	108										4.2					
butan-1-ol	1900	1120															
butanoic acid	2400	1180										620	2490	1740	660	1180	2060
octan-1-ol	110	44.7															
ethyl 2-phenylacetate	155.55	53.8															
2-methylbutanoic acid	3100	545															
ethyl dodecanoate	3500	269															
propanoic acid	20000	1490															
2-methylpropanoic acid	60000	2180										1870					

		reference no.	73	73	73	73	73	73	74	74	74	74	74	74	75	76	77
		wine sample no.	226	227	228	229	230	231	232	233	234	235	236	237	238	239	240
matrix		mean															
ethanol (%, ALC/VOL)		12.9															
pH		3.4															
odorant	OTC (µg/kg) in water	mean concentration (µg/L)															
ethyl acetate	5	69100							52833	242493	70197	95113	231870	132700			
acetaldehyde	16	49100	56600	33100	4870	56000	46600	57200	240670	284537	54270	154640	225153	145450	45900		
butane-2,3-dione	1.0	1400							146	77.5	200	207	81.3	95.4	1250		350
ethyl hexanoate	1.2	1570							46.3	9.8	61.9	20.2	61.3	68.7	425	487	282
ethyl 3-methylbutanoate	0.023	27.5							143	96.3	393	95.1	76.9	69.1	43.1	37.3	11.1
ethyl 2-methylpropanoate	0.089	93.5							65.2	39.0	176	39.7	27.9	36.1	211	140	35.3
3-methylbutan-1-ol	220	172000							163733	112200	164573	205153	144350	161690	248000	188000	147207
3-methylbutyl acetate	7.2	3650	930	350	320	1100	980	680	39.4	51.7	120	97.7	187	222	437		553
ethyl butanoate	0.76	374							32.7	21.9	41.9	23.7	32.7	29.3	386	124	115
ethyl 2-methylbutanoate	0.13	42.7	12.5	21.1	17.1	4.2	12.1	18.4	98.1	79.2	411	87.2	54.1	84.6	14.9	19.9	7
ethyl octanoate	8.7	2460							7.7	6.2	8.0	1.8	51.3	68.6	196	172	358
3-methylbutanal	0.50	119															
2-phenylethan-1-ol	140	28700	73100	58000	67100	54500	67000	65500	45910	33217	49537	78203	35970	45833	58800	24900	70210
2-methylpropanal	0.49	36.5															
2-methylbutan-1-ol	1200	70100															
dimethyl sulfide	0.30	14.1															
acetic acid	5600	219000							281990	327707	408427	382963	328937	310067		189000	189000
3-(methylsulfanyl)propan-1-ol	36	1360	8020	6510	3370	8100	8640	8210	697	816	1223	1965	760	910	3660	1750	856
3-(methylsulfanyl)propanal	0.43	14.6															
ethyl propanoate	10	295															
octanoic acid	190	5580	2560	1810	1480	2000	2320	2680	312	340	238	254	529	431	2500	1550	1915
3-hydroxybutan-2-one	590	16600							2713	6760	3810	1617	3307	2207	54900	480	33207
2-methylbutanal	1.5	40.2															
phenylacetic acid	68	452							216	232	89.5			298	59.4		
ethyl decanoate	122	741							15.4	14.7	18.3	14.8	27.8	29.3	18.7		100
decanoic acid	500	2460							137	198	124	121	175	176	671		382
hexan-1-ol	590	2710	3590	3260	1950	3700	3470	3720	118	380	1280	396	526	464	3500	2210	7800
phenylacetaldehyde	5.2	21.5		6.84	6.84			1.93	3.8	3.7	3.4	4.5	3.3	6.1	4.70		
2-phenylethyl acetate	360	682							31.1	25.2	22.0	26.3	25.6	21.8	21.9		26.9
2-methylpropan-1-ol	19000	33000							82190	80947	58430	72320	67497	78723	84800		46500
3-methylbutanoic acid	490	814	2150	1690	1350	2500	2230	2270	1867	1267	5257	3441	1097	2346	1810	760	604
2-methylpropyl acetate	66	101							34.1	73.4	41.1	16.1	90.7	172	35.7	71.0	
hexanoic acid	4800	4060	2560	2040	2530	2200	2280	2370	399	323	470	366	642	515	2820	1550	1724
benzaldehyde	150	108							5.3	2.6	3.3	12.1	20.5	30.1			
butan-1-ol	1900	1120													1730		
butanoic acid	2400	1180	1350	570	1050	600	1690	1210	2648	1114	3537	3457	1331	3358	2020	838	974
octan-1-ol	110	44.7															
ethyl 2-phenylacetate	155.55	53.8															
2-methylbutanoic acid	3100	545													204		
ethyl dodecanoate	3500	269															
propanoic acid	20000	1490							2122	1556	6195	6159	1069	3151	4160		
2-methylpropanoic acid	60000	2180							4469	2316	13995	8919	1701	4865	3510	1460	1134

		reference no.	78	79	79	79	79	80	81	81	81	81	81	81	81	81	81
		wine sample no.	241	242	243	244	245	246	247	248	249	250	251	252	253	254	255
matrix		mean															
ethanol (%, ALC/VOL)		12.9							14.77	14.69	14.82	14.66	17.91	17.73	17.86	17.68	14.34
pH		3.4							3.01	3.08	3.07	3.07	2.98	2.99	2.98	2.98	3.35
odorant	OTC (µg/kg) in water	mean concentration (µg/L)															
ethyl acetate	5	69100	53828					117000	58490	83660	64520	41860	51970	62890	52040	48510	64370
acetaldehyde	16	49100															
butane-2,3-dione	1.0	1400															
ethyl hexanoate	1.2	1570	467					1076	361.31	555.12	414.17	271.97	342.78	391.36	280.57	232.48	288.88
ethyl 3-methylbutanoate	0.023	27.5	24.7						2.57	2.95	2.73	2.79	4.07	3.96	4.08	3.83	3.13
ethyl 2-methylpropanoate	0.089	93.5						80									
3-methylbutan-1-ol	220	172000	523877					162000	260100	270950	294790	256680	224670	229710	223200	207070	254280
3-methylbutyl acetate	7.2	3650	1249	449.2	573.5	483.4	386	3528	287.14	417.00	365.18	342.91	201.22	310.27	305.31	302.26	382.14
ethyl butanoate	0.76	374	249					695	260.10	422.74	251.05	184.12	232.99	244.30	170.31	160.74	159.71
ethyl 2-methylbutanoate	0.13	42.7							3.41	2.73	3.38	3.17	3.50	3.86	3.99	2.22	1.80
ethyl octanoate	8.7	2460	357	37.9	35.1	37.3	61.1	1468	171.19	309.87	247.56	148.21	232.65	296.96	201.46	152.80	130.27
3-methylbutanal	0.50	119															
2-phenylethan-1-ol	140	28700	144438					19000	24750	17690	20820	23560	23780	21430	21910	21160	21330
2-methylpropanal	0.49	36.5															
2-methylbutan-1-ol	1200	70100						30000									
dimethyl sulfide	0.30	14.1															
acetic acid	5600	219000							280000	300000	370000	390000	200000	470000	370000	130000	500000
3-(methylsulfanyl)propan-1-ol	36	1360							105.27	84.92	123.47	143.71	102.96	98.07	127.90	153.40	221.98
3-(methylsulfanyl)propanal	0.43	14.6															
ethyl propanoate	10	295						179									
octanoic acid	190	5580	2114					10000	3537.02	4631.18	4100.89	3219.52	4261.98	3990.88	3355.84	3190.36	3576.79
3-hydroxybutan-2-one	590	16600															
2-methylbutanal	1.5	40.2															
phenylacetic acid	68	452															
ethyl decanoate	122	741	72.9					515	65.28	42.70	45.31	14.25	70.89	82.04	56.46	29.78	31.85
decanoic acid	500	2460	149					2390									
hexan-1-ol	590	2710	1869					1152	954.18	948.77	902.40	853.42	838.43	781.33	712.71	798.18	2020.95
phenylacetaldehyde	5.2	21.5															
2-phenylethyl acetate	360	682	274	35.2	46.7	39.1	24.9	299	23.91	18.75	19.87	23.83	22.27	25.38	22.87	14.91	35.58
2-methylpropan-1-ol	19000	33000	52722					33000									
3-methylbutanoic acid	490	814						1717	424.86	310.26	439.13	414.41	318.40	328.26	388.00	319.32	323.93
2-methylpropyl acetate	66	101															
hexanoic acid	4800	4060	1154					12000	1824.83	2973.50	2209.76	1509.15	2030.04	1844.35	1451.38	1316.03	1882.76
benzaldehyde	150	108							16.14	20.31	16.66	17.85	15.42	15.45	15.60	16.59	14.50
butan-1-ol	1900	1120							371.05	241.87	186.51	241.95	441.60	220.49	190.27	205.29	326.74
butanoic acid	2400	1180															
octan-1-ol	110	44.7	18.3	14.9	16.7	18.7	14.0										
ethyl 2-phenylacetate	155.55	53.8	14.1														
2-methylbutanoic acid	3100	545															
ethyl dodecanoate	3500	269															
propanoic acid	20000	1490															
2-methylpropanoic acid	60000	2180	2343														

		reference no.	81	81	81	81	81	81	81	82	82	82	82	82	82	82	82
		wine sample no.	256	257	258	259	260	261	262	263	264	265	266	267	268	269	270
matrix		mean															
ethanol (%, ALC/VOL)		12.9	14.17	14.24	14.54	15.50	15.41	15.31	15.32	12.4	12.1	11.9	13.1	13.8	16.1	13.7	13.7
pH		3.4	3.30	3.32	3.31	3.21	3.22	3.22	3.23	3.11	3.18	3.33	3.14	3.21	3.37	3.39	3.3
odorant	OTC (µg/kg) in water	mean concentration (µg/L)															
ethyl acetate	5	69100	101320	62120	44990	57700	70320	69850	54020								
acetaldehyde	16	49100															
butane-2,3-dione	1.0	1400															
ethyl hexanoate	1.2	1570	441.20	371.86	294.37	415.84	567.81	358.27	439.93	644	745	631	694	811	679	721	798
ethyl 3-methylbutanoate	0.023	27.5	3.10	3.22	3.06	3.11	3.10	3.21	2.95	47.0	35.1	13.4	38.4	24.8	20.5	28.5	14.5
ethyl 2-methylpropanoate	0.089	93.5															
3-methylbutan-1-ol	220	172000	27140	28440	200640	25800	32460	266040	312920	153598	156644	174393	149647	140460	191163	177300	215633
3-methylbutyl acetate	7.2	3650	707.32	482.77	258.90	702.55	689.58	450.43	373.92	751	904	3191	872	3722	3683	2360	1660
ethyl butanoate	0.76	374	206.20	184.08	168.03	267.28	366.95	201.25	241.66	421	330	353	329	455	365	409	460
ethyl 2-methylbutanoate	0.13	42.7	3.57	2.46	1.21	1.83	2.53	2.31	2.93	25.5	20.7	7.4	21.9	11.2	8.8	15.7	7.7
ethyl octanoate	8.7	2460	204.82	182.78	117.46	221.11	320.36	236.13	282.17	813	807	1375	2440	1245	1011	1034	976
3-methylbutanal	0.50	119								10.9	8.8	18.5	17.5	16.4	13.6	10.5	13.3
2-phenylethan-1-ol	140	28700	19420	19270	10950	21670	26480	20010	24690	19025	17591	18053	20571	14866	25103	18366	21511
2-methylpropanal	0.49	36.5								7.9	9.1	12.8	12.6	7.1	6.00	10.7	8.50
2-methylbutan-1-ol	1200	70100								24938	27389	28846	28293	22088	29583	29667	31450
dimethyl sulfide	0.30	14.1															
acetic acid	5600	219000	600000	590000	440000	230000	410000	280000	210000								
3-(methylsulfanyl)propan-1-ol	36	1360	135.32	222.60	389.66	116.97	217.18	129.93	259.07								
3-(methylsulfanyl)propanal	0.43	14.6								2.18	3.60	4.26	5.85	7.62	5.01	2.22	5.36
ethyl propanoate	10	295															
octanoic acid	190	5580	4432.38	4197.55	3798.66	4021.06	5095.48	3948.40	4166.92	3969	3612	4162	3959	4135	3819	3575	3338
3-hydroxybutan-2-one	590	16600															
2-methylbutanal	1.5	40.2								2.75	2.73	3.11	2.40	1.73	1.25	3.33	2.00
phenylacetic acid	68	452															
ethyl decanoate	122	741	45.66	54.99	29.99	51.72	85.86	67.95	75.81	211	192	534	646	494	351	279	178
decanoic acid	500	2460								2191	1798	1554	1753	2124	1949	2015	1464
hexan-1-ol	590	2710	1940.31	1737.24	1701.24	1149.65	1424.44	1054.97	1154.77	542	1161	1128	1309	1423	1048	962	1948
phenylacetaldehyde	5.2	21.5															
2-phenylethyl acetate	360	682	29.21	25.74	24.61	26.14	26.53	14.21	14.32	121	117	233	82	212	553	196	214
2-methylpropan-1-ol	19000	33000								25468	23267	32340	25467	16239	23083	24550	33883
3-methylbutanoic acid	490	814	364.47	486.21	253.57	374.47	432.56	364.05	459.10	398	397	566	552	497	400	416	322
2-methylpropyl acetate	66	101								22.3	24.0	75.4	30.4	55.8	50	255	447
hexanoic acid	4800	4060	2938.93	2359.40	1986.87	2194.09	3172.54	2056.21	2299.39	4075	3746	3922	4714	5144	3609	3415	3573
benzaldehyde	150	108	14.30	15.03	14.75	14.50	15.03	14.19	14.84								
butan-1-ol	1900	1120	228.80	158.29	100.38	435.89	246.54	202.54	161.62	178	711	536	883	929	408	933	583
butanoic acid	2400	1180								1244	1049	1550	1695	1931	1166	1211	1221
octan-1-ol	110	44.7															
ethyl 2-phenylacetate	155.55	53.8															
2-methylbutanoic acid	3100	545															
ethyl dodecanoate	3500	269															
propanoic acid	20000	1490															
2-methylpropanoic acid	60000	2180								713	591	1375	1417	988	806	654	554

		reference no.	82	83	83	83	83	83	83	83	83	83	83	83	83	83	84
		wine sample no.	271	272	273	274	275	276	277	278	279	280	281	282	283	284	285
matrix		mean															
ethanol (%, ALC/VOL)		12.9	13.2	13	12.5	12.5	12.5	12.5	13.0	13.5	13.0	12.5	12.5	13.0	12.5	12.0	12.12
pH		3.4	3.41	3.45	2.98	3.1	3.16	3.27	3.18	3.27	3.4	3.22	3.27	3.07	3.38	3.25	
odorant	OTC (µg/kg) in water	mean concentration (µg/L)															
ethyl acetate	5	69100															30580
acetaldehyde	16	49100															10590
butane-2,3-dione	1.0	1400															
ethyl hexanoate	1.2	1570	715	642	260	779	754	736	845	551	668	549	538	677	705	567	60
ethyl 3-methylbutanoate	0.023	27.5	16.1	5.34	24.27	16.45	12.4	4.43	15.58	9.85	3.33	23.07	2.62	11.11	10.36	6.95	
ethyl 2-methylpropanoate	0.089	93.5															
3-methylbutan-1-ol	220	172000	172851	79262	77943	68735	92012	72668	111837	85313	83035	87285	87624	84250	98730	80724	64500
3-methylbutyl acetate	7.2	3650	2225	952	102	857	1262	1646	294	484	228	340	388	766	1056	356	2017
ethyl butanoate	0.76	374	353	254	134	248	250	253	333	232	279	209	297	327	333	132	410
ethyl 2-methylbutanoate	0.13	42.7	8.5	3.08	1.18	3.19	6.91	1.94	9.49	4.29	5.59	14.53	1.38	5.65	3.09	4.02	
ethyl octanoate	8.7	2460	935	990	435	648	591	740	894	638	801	365	556	769	652	562	
3-methylbutanal	0.50	119	16.5														
2-phenylethan-1-ol	140	28700	22325	10840	7162	4230	6148	4225	5253	14404	8927	8457	9545	11212	9083	9637	30100
2-methylpropanal	0.49	36.5	7.80														
2-methylbutan-1-ol	1200	70100	26758														
dimethyl sulfide	0.30	14.1		1.69	1.7	1.46	1.06	0.98	1.96	0.21	3.1	1.66	1.4	0.38	0.69	1.16	
acetic acid	5600	219000															
3-(methylsulfanyl)propan-1-ol	36	1360		98.1	94.4	84.4	105	68.9	75.4	174.5	98.8	173.3	156.5	75.3	176.7	191.6	
3-(methylsulfanyl)propanal	0.43	14.6	4.68														
ethyl propanoate	10	295															
octanoic acid	190	5580	3546	10053	8636	8437	7569	8407	9318	8452	9137	6767	8385	8233	7986	8596	
3-hydroxybutan-2-one	590	16600															
2-methylbutanal	1.5	40.2	2.7														
phenylacetic acid	68	452															
ethyl decanoate	122	741	288	223	52	124	84	120	120	117	210	60	89	120	141	95	
decanoic acid	500	2460	1064														
hexan-1-ol	590	2710	1623	727	1027	694	720	665	1099	934	930	982	1094	710	1029	1046	
phenylacetaldehyde	5.2	21.5															
2-phenylethyl acetate	360	682	268	298.6	26.9	63.5	82.2	82.7	24.5	134.7	45.8	47.7	94.1	134	88.9	87.6	
2-methylpropan-1-ol	19000	33000	23609														14330
3-methylbutanoic acid	490	814	313	247	259	162	205	163	124	305	193	188	235	177	226	278	
2-methylpropyl acetate	66	101	130														
hexanoic acid	4800	4060	3145	6282	5655	4171	4222	4806	6640	4406	5035	3397	5385	4245	4083	5305	
benzaldehyde	150	108		17.1	16.2	19.8	59.8	34.3	19.1	16.7	25.6	26.3	16.2	15.6	17.8	14.8	
butan-1-ol	1900	1120	650	43.3	27.2	16.9	54.4	60	82.4	70.9	93.2	124.5	52.9	82.7	52	58.1	
butanoic acid	2400	1180	958														
octan-1-ol	110	44.7															
ethyl 2-phenylacetate	155.55	53.8															
2-methylbutanoic acid	3100	545															
ethyl dodecanoate	3500	269															
propanoic acid	20000	1490															
2-methylpropanoic acid	60000	2180	574														

		reference no.	85	86	87	88	88	89	90	91	91	91	91	92	92	93	93
		wine sample no.	286	287	288	289	290	291	292	293	294	295	296	297	298	299	300
matrix		mean															
ethanol (%, ALC/VOL)		12.9	14.5	13.5		12.5	13.0	12.35						14.4	12.7	13.3	
pH		3.4	3.73	3.66		3.43	3.48	3.42								3.42	
odorant	OTC (µg/kg) in water	mean concentration (µg/L)															
ethyl acetate	5	69100		4588	30905	42600	40600			2770	3870	3590	2010	123800	228900	23200	23200
acetaldehyde	16	49100				4500	7000			10	25	32	18				
butane-2,3-dione	1.0	1400				400	210										
ethyl hexanoate	1.2	1570	70	597	817	220	250	337	217.70					200	410		315
ethyl 3-methylbutanoate	0.023	27.5		16		12.5	11.1		4.14					1			7.2
ethyl 2-methylpropanoate	0.089	93.5		70		90	67							30	8		29.6
3-methylbutan-1-ol	220	172000	122		800050	267000	295000			38600	22500	39500	38500	56000		114000	103200
3-methylbutyl acetate	7.2	3650		2346	1346	290	290	1766	1437.24	1610	2420	1380	2000	240	3170	2649	1907
ethyl butanoate	0.76	374	37	381	140	90	88	164	12.62					200	370		206
ethyl 2-methylbutanoate	0.13	42.7	24	9		8.7	8.5							1	3		10.8
ethyl octanoate	8.7	2460	20	652	754	230	210	321	262.41	5440	13000	1320	956	310	430		528
3-methylbutanal	0.50	119		11													4.4
2-phenylethan-1-ol	140	28700	9850		128040	38700	77000		24303.25	6240	4750	3010	3260	32700	23900	19800	18300
2-methylpropanal	0.49	36.5		12													5.4
2-methylbutan-1-ol	1200	70100												53600		19700	29400
dimethyl sulfide	0.30	14.1		39										2.6	1.1		3.0
acetic acid	5600	219000			1410000	391000	106000	380000		739	245	842	326	335200	293400	390000	226800
3-(methylsulfanyl)propan-1-ol	36	1360		1253		1060	2300		105.56								542
3-(methylsulfanyl)propanal	0.43	14.6															1.71
ethyl propanoate	10	295		401		96	190							70	430		25.1
octanoic acid	190	5580	350		3270	1670	1700		1020.51	201	677	40	94	2020	2280		8024
3-hydroxybutan-2-one	590	16600				1200	400			12	5	14	6				
2-methylbutanal	1.5	40.2		11													
phenylacetic acid	68	452							10.19								
ethyl decanoate	122	741	2	1142	530			85.8	22.68					170	536		172
decanoic acid	500	2460	440		190	1130	3000							880	2610		1792
hexan-1-ol	590	2710	350	3463	4910	1420	1440		227.32	1000	956	481	176	2600	3700		1207
phenylacetaldehyde	5.2	21.5		8							5	2					5.1
2-phenylethyl acetate	360	682	3		132	665	932	213	76.36					200	860	280	228
2-methylpropan-1-ol	19000	33000		46780	39150	57000	39000		38.68	2720	421	4		53500	65000	28500	21100
3-methylbutanoic acid	490	814				3900	3500		435.9	275	89	2	156	160			822
2-methylpropyl acetate	66	101		93	41	53	42	51.7		72	2	5	2	130	150	89	70
hexanoic acid	4800	4060			4240	2100	2300			135	251	85	60	1540			5659
benzaldehyde	150	108		41					6.53	12	3		1				61
butan-1-ol	1900	1120	250	1317		403	640		13.16					830			446
butanoic acid	2400	1180				470	500		5.87					440			1211
octan-1-ol	110	44.7							1.41	142	89	502	344				
ethyl 2-phenylacetate	155.55	53.8															
2-methylbutanoic acid	3100	545								3	5	2	5	290			402
ethyl dodecanoate	3500	269	5														
propanoic acid	20000	1490												700			1077
2-methylpropanoic acid	60000	2180				5100	3100		53.62	117	55	129	88	2450	2590		

		reference no.	93	94	95	96	96	97	97	97	98	98	99	99	99	99	99
		wine sample no.	301	302	303	304	305	306	307	308	309	310	311	312	313	314	315
matrix		mean															
ethanol (%, ALC/VOL)		12.9		13.9	12.5	12.9	13.5	13.6	15.0	15.2			12.49	12.50	12.25	12.14	12.51
pH		3.4		3.2	3.46	3.78	3.74	2.96	2.92	3.03			3.50	3.50	3.50	3.51	3.51
odorant	OTC (µg/kg) in water	mean concentration (µg/L)															
ethyl acetate	5	69100	38100		41310	64955	76925	19537.4	49654.9	33649.2							
acetaldehyde	16	49100				816	333										
butane-2,3-dione	1.0	1400				417	658										
ethyl hexanoate	1.2	1570	646	1916	543.51	586	744	548.5	331.0	302.1	435.57	409.18	191	210	190	175	148
ethyl 3-methylbutanoate	0.023	27.5	27.3	1.96		42	22	215.4	64.6	36.7							
ethyl 2-methylpropanoate	0.089	93.5	178	8.4		100	51										
3-methylbutan-1-ol	220	172000	144000	194812	274860	334668	219489	35372.3	33820.8	34951.5	175376.59	182619.15	55000	55000	53000	55000	53000
3-methylbutyl acetate	7.2	3650	890	13292	801.25	111	190	10732.9	3216.1	1877.3	881.39	289.54	1218	1968	2235	2497	1990
ethyl butanoate	0.76	374	329	608	646.55			254.7	265.6	210.1	409.91	329.68					
ethyl 2-methylbutanoate	0.13	42.7	14.4			27	12				10.71	18.69					
ethyl octanoate	8.7	2460	954	1455	33.21	191	277	452.1	254.3	249.6	574.99	589.69	313	365	485	560	385
3-methylbutanal	0.50	119	26.3														
2-phenylethan-1-ol	140	28700	26200	37583	25740	39602	24696	16240.6	20779.8	16568.9	35264.82	46394.58	22000	20000	19000	19000	19000
2-methylpropanal	0.49	36.5	128														
2-methylbutan-1-ol	1200	70100	29600														
dimethyl sulfide	0.30	14.1	15.9			43	23										
acetic acid	5600	219000	434300			429857	442911										
3-(methylsulfanyl)propan-1-ol	36	1360	567	1471		2414	1064						836	689	780	515	478
3-(methylsulfanyl)propanal	0.43	14.6	26.1														
ethyl propanoate	10	295	89			137	240										
octanoic acid	190	5580	10423	9500	1840	1666	1915	2044.8	820.0	1066.7	1443	1191	3267	3270	3469	3406	3525
3-hydroxybutan-2-one	590	16600				11052	22410						72000	91000	106000	151000	87000
2-methylbutanal	1.5	40.2															
phenylacetic acid	68	452															
ethyl decanoate	122	741	319	524				45.6	50.6	38.6	272.75	263.47	205	384	447	652	348
decanoic acid	500	2460	2221	1000	173.81	247	433				269	231	1588	978	574	608	587
hexan-1-ol	590	2710	1982	380	5290	1849	1864	3292.5	2292.4	1951.1	3430.80	2273.37	2570	2014	2745	3109	2348
phenylacetaldehyde	5.2	21.5	25.8								49.04	54.12					
2-phenylethyl acetate	360	682	135	1122	2.70	4.1	5.6	106.2	35.3	30.7	69.94	71.72	288	304	539	730	394
2-methylpropan-1-ol	19000	33000	27400	25484		63758	25701				33347.22	35407.67	48000	39000	41000	42000	40000
3-methylbutanoic acid	490	814	645			1719	943	511.9	402.3	449.1			68	150	175	140	124
2-methylpropyl acetate	66	101	28.9	15.1		8.7	5.8										
hexanoic acid	4800	4060	7048	6800	2740	1382	1684	2623.6	1286.5	1683.5	2643	2232	12	28	38	47	37
benzaldehyde	150	108	130	0.64	38.50	11	2.0										
butan-1-ol	1900	1120	733	1340		781	921	269.7	506.3	228.0	3911.73	3818.76					
butanoic acid	2400	1180	846		1040	186	186						23	65	88	41	51
octan-1-ol	110	44.7			26.11						41.16	15.45					
ethyl 2-phenylacetate	155.55	53.8															
2-methylbutanoic acid	3100	545	371														
ethyl dodecanoate	3500	269		44	29.45						394.97	364.73					
propanoic acid	20000	1490	1140														
2-methylpropanoic acid	60000	2180		1080	1740	3139	4726										

		reference no.	99	99	99	99	99	100	101	102	103	103	103	104	105	106	106
		wine sample no.	316	317	318	319	320	321	322	323	324	325	326	327	328	329	330
matrix		mean															
ethanol (%, ALC/VOL)		12.9	12.25	12.14	12.32	12.17	12.10							13.7	12.68	13.3	13.5
pH		3.4	3.52	3.51	3.52	3.52	3.53							3.3		3.27	3.28
odorant	OTC (µg/kg) in water	mean concentration (µg/L)															
ethyl acetate	5	69100						2852.26	49026	6780	58460	60400	53380				
acetaldehyde	16	49100															
butane-2,3-dione	1.0	1400									8490	12740	11560				
ethyl hexanoate	1.2	1570	216	113	195	128	114	27.27	590	3150	266.37	239.48	186.53	316.08	297	9949	8841
ethyl 3-methylbutanoate	0.023	27.5						0.12						26.56	2.8		
ethyl 2-methylpropanoate	0.089	93.5						1.29	40.3						13.0		
3-methylbutan-1-ol	220	172000	54000	55000	49000	52000	53000	172254.5	397197		294300	314710	294320	33530		105941	99225
3-methylbutyl acetate	7.2	3650	2308	2512	1955	1975	2183		5652	480	3190	3520	2840	1690	2005	146465	120426
ethyl butanoate	0.76	374						20.35			225.08	272.14	192.20	281.50	154	1157	952
ethyl 2-methylbutanoate	0.13	42.7						1.70	5.70					18.41	1.95		
ethyl octanoate	8.7	2460	412	588	354	461	520	33.77	489	1330	232.32	237.02	196.58	585.83	292	2401	2127
3-methylbutanal	0.50	119															
2-phenylethan-1-ol	140	28700	19000	19000	19000	20000	19000	213589.2	91055	85960	28830	36210	34520	32930		25018	22253
2-methylpropanal	0.49	36.5															
2-methylbutan-1-ol	1200	70100												31400			
dimethyl sulfide	0.30	14.1															
acetic acid	5600	219000								74030							
3-(methylsulfanyl)propan-1-ol	36	1360	654	363	471	647	483	6210.62	3484		257.90	394.41	438.89				
3-(methylsulfanyl)propanal	0.43	14.6															
ethyl propanoate	10	295						31.97	313						124		
octanoic acid	190	5580	3408	3518	3566	3979	3626		719	360	1435.57	1342.16	1262.94	8.50		6543	5811
3-hydroxybutan-2-one	590	16600	102000	130000	84000	127000	197000				1260	1580	910	4.97			
2-methylbutanal	1.5	40.2															
phenylacetic acid	68	452															
ethyl decanoate	122	741	442	534	344	433	523	522.35		28600	40.80	45.09	43.35	526.86	58.6	1080	988
decanoic acid	500	2460	518	460	690	589	562			780	302.32	285.21	270.44			3072	2696
hexan-1-ol	590	2710	2565	2535	2382	2613	3123	1143.52	2463	620	1210	1190	1160	1050	1930	1552	1358
phenylacetaldehyde	5.2	21.5															
2-phenylethyl acetate	360	682	333	402	305	526	623		119	3770	143.25	192.73	231.98		238	16103	13518
2-methylpropan-1-ol	19000	33000	43000	44000	39000	40000	42000	37440.21	76089	127370	27190	30210	30020			49309	42767
3-methylbutanoic acid	490	814	163	142	156	166	140			2050	116.44	144.09	151.78	4.41			
2-methylpropyl acetate	66	101						5.99	23.5					54.17	57.3	712	568
hexanoic acid	4800	4060	57	66	29	37	47		4725	70	1488.21	1443.82	1183.28			7286	6598
benzaldehyde	150	108						2.38						17.60		82.8	77.2
butan-1-ol	1900	1120							8.19	1770	990	1430	1480	28.21			
butanoic acid	2400	1180	91	47	67	72	42				96.23	87.68	82.71				
octan-1-ol	110	44.7							30.2					5.43			
ethyl 2-phenylacetate	155.55	53.8						0.56	5.38						2.17		
2-methylbutanoic acid	3100	545															
ethyl dodecanoate	3500	269						3.48	16.1	370	1.31	0.85	0.27		4.62	1162	1170
propanoic acid	20000	1490									5.53	5.86	6.03	29.95			
2-methylpropanoic acid	60000	2180								2550	77.34	105.29	136.41	7.77			

		reference no.	106	106	107	107	108	108	108	108	108	108	109	109	109	109	109
		wine sample no.	331	332	333	334	335	336	337	338	339	340	341	342	343	344	345
matrix		mean															
ethanol (%, ALC/VOL)		12.9	13.3	13.4	12.91	11.57	14.0	14.0	11.0	12.0	13.0	13.0	13.38	13.59	12.95	13.89	13.29
pH		3.4	3.32	3.25	3.25	3.44	3.2	3.1	3.1	3.3	3.3	3.2	3.42	3.54	3.15	3.55	3.20
odorant	OTC (µg/kg) in water	mean concentration (µg/L)															
ethyl acetate	5	69100					24860	20890	11130	17070	4440	4130	30340	33370	41340	33890	68290
acetaldehyde	16	49100											76350	83040	74900	62440	93290
butane-2,3-dione	1.0	1400															
ethyl hexanoate	1.2	1570	10290	9261	380	1100	6940	270	120				134.00	145.00	181.50	162.00	167.45
ethyl 3-methylbutanoate	0.023	27.5															
ethyl 2-methylpropanoate	0.089	93.5															
3-methylbutan-1-ol	220	172000	109098	109286	82070	228460	49090	133190	59120	67180	12990	20330	124000	138000	128500	124000	138000
3-methylbutyl acetate	7.2	3650	144754	124893	2480	3680	2390	1510	320	200	90	90	208.50	239.00	265.50	319.00	238.50
ethyl butanoate	0.76	374	1134	871.9			280	270	140	100	10	10	16.63	22.73	30.10	19.70	24.09
ethyl 2-methylbutanoate	0.13	42.7															
ethyl octanoate	8.7	2460	2460	2152	1670	6290	460	540	190	100	1050	2010	366.50	382.50	303.50	301.00	233.00
3-methylbutanal	0.50	119															
2-phenylethan-1-ol	140	28700	24749	25322	29520	47600	3160	8760	3100	3440	1220	1590	13170	12420	14170	12320	14020
2-methylpropanal	0.49	36.5															
2-methylbutan-1-ol	1200	70100											31550	48050	50450	32650	29650
dimethyl sulfide	0.30	14.1															
acetic acid	5600	219000					133720	142200	91810	108250	195640	208620	4.82	3.67	4.34	4.58	3.88
3-(methylsulfanyl)propan-1-ol	36	1360			3700	3680							176.50	238.00	218.50	209.00	212.50
3-(methylsulfanyl)propanal	0.43	14.6															
ethyl propanoate	10	295															
octanoic acid	190	5580	6735	6358	3220	10480	3490	4480	2200	1440	520	550	474.00	381.00	404.00	387.50	398.50
3-hydroxybutan-2-one	590	16600					200	200	70	70	240	310					
2-methylbutanal	1.5	40.2															
phenylacetic acid	68	452															
ethyl decanoate	122	741	1037	662			170	170	60	40	10	10	179.00	198.00	180.50	192.50	168.00
decanoic acid	500	2460	3038	2071									350.50	253.50	298.00	253.00	262.00
hexan-1-ol	590	2710	1426	1233	1050	2330	1760	1410	1820	1490	990	1070	439.17	339.50	627.00	352.50	308.00
phenylacetaldehyde	5.2	21.5															
2-phenylethyl acetate	360	682	14613	14979			980	1130	630	450	90	90	55.15	46.25	38.05	24.25	33.95
2-methylpropan-1-ol	19000	33000	41271	45559	37710	45120	4550	11140	7710	7960	190	190	24130	35370	42060	47130	34450
3-methylbutanoic acid	490	814					670	990	490	480	250	330	170.00	214.00	193.50	267.00	196.50
2-methylpropyl acetate	66	101	647	528			20	20	30	30							
hexanoic acid	4800	4060	7472	6978	4050	16270	50470	2950	62420	53700	98490	99090	441.50	360.00	417.00	361.00	404.00
benzaldehyde	150	108	296.0	79.9									5.88	5.56	3.83	4.81	6.60
butan-1-ol	1900	1120					650	880	130	90			1.67	2.72	3.41	1.74	2.10
butanoic acid	2400	1180					680	620	130	70			16.90	20.25	19.10	14.67	17.25
octan-1-ol	110	44.7															
ethyl 2-phenylacetate	155.55	53.8					260	240	210	230	250	260					
2-methylbutanoic acid	3100	545															
ethyl dodecanoate	3500	269	1246	967	150	590											
propanoic acid	20000	1490					6810	1260	70	40	1090	1140	1.44	2.37	1.64	1.43	2.51
2-methylpropanoic acid	60000	2180					440	680	320	360	120	120	258.00	294.00	304.00	317.00	320.00

		reference no.	110	110	110	110	110	110	110	110	110	110	110	110	110	110	110
		wine sample no.	346	347	348	349	350	351	352	353	354	355	356	357	358	359	360
matrix		mean															
ethanol (%, ALC/VOL)		12.9															
pH		3.4															
odorant	OTC (µg/kg) in water	mean concentration (µg/L)															
ethyl acetate	5	69100															
acetaldehyde	16	49100															
butane-2,3-dione	1.0	1400															
ethyl hexanoate	1.2	1570															
ethyl 3-methylbutanoate	0.023	27.5															
ethyl 2-methylpropanoate	0.089	93.5															
3-methylbutan-1-ol	220	172000															
3-methylbutyl acetate	7.2	3650															
ethyl butanoate	0.76	374															
ethyl 2-methylbutanoate	0.13	42.7															
ethyl octanoate	8.7	2460															
3-methylbutanal	0.50	119	225	454	70	63	110	181	199	100	169	36	477	212	166	357	108
2-phenylethan-1-ol	140	28700															
2-methylpropanal	0.49	36.5	44	51	26	18	38	66	68	95	77	35	41	23.00	24	68	32
2-methylbutan-1-ol	1200	70100															
dimethyl sulfide	0.30	14.1															
acetic acid	5600	219000															
3-(methylsulfanyl)propan-1-ol	36	1360															
3-(methylsulfanyl)propanal	0.43	14.6	16	11	4.7	8.3	6.7	9.3	17	7.5	25	2.8	2.7	31	14	8.2	3.5
ethyl propanoate	10	295															
octanoic acid	190	5580															
3-hydroxybutan-2-one	590	16600															
2-methylbutanal	1.5	40.2															
phenylacetic acid	68	452															
ethyl decanoate	122	741															
decanoic acid	500	2460															
hexan-1-ol	590	2710															
phenylacetaldehyde	5.2	21.5	22	26.0	7.2	9.5	6.9	12	16.0	4.9	29.0	1.2	13	18	14.0	6.0	5.9
2-phenylethyl acetate	360	682															
2-methylpropan-1-ol	19000	33000															
3-methylbutanoic acid	490	814															
2-methylpropyl acetate	66	101															
hexanoic acid	4800	4060															
benzaldehyde	150	108	57	8.1	62	10	5.0	2.9	3.20	1.3	9.3	1.7	11	9.6	4.7	3.1	2.6
butan-1-ol	1900	1120															
butanoic acid	2400	1180															
octan-1-ol	110	44.7															
ethyl 2-phenylacetate	155.55	53.8															
2-methylbutanoic acid	3100	545															
ethyl dodecanoate	3500	269															
propanoic acid	20000	1490															
2-methylpropanoic acid	60000	2180															

		reference no.	110	110	110	110	110	111	111	111	111	111	111	112	112	112	112
		wine sample no.	361	362	363	364	365	366	367	368	369	370	371	372	373	374	375
matrix		mean															
ethanol (%, ALC/VOL)		12.9												11.86	12.39	12.46	12.41
pH		3.4												3.94	4.01	4.05	4.07
odorant	OTC (µg/kg) in water	mean concentration (µg/L)															
ethyl acetate	5	69100						61000	24000	26000	63000	20000	30000	55638.68	73720.51	65773.82	54064.14
acetaldehyde	16	49100						65000	38000	22000	43000	29000	19000				
butane-2,3-dione	1.0	1400															
ethyl hexanoate	1.2	1570						440	350	260	370	290	230	288.85	202.25	519.39	427.21
ethyl 3-methylbutanoate	0.023	27.5															
ethyl 2-methylpropanoate	0.089	93.5															
3-methylbutan-1-ol	220	172000						250000	239000	159000	238000	234000	215000	435327.94	515279.31	511874.25	498725.37
3-methylbutyl acetate	7.2	3650						300	1100	900	200	300	700	756.79	867.13	792.31	370.43
ethyl butanoate	0.76	374						100		80	110		80				
ethyl 2-methylbutanoate	0.13	42.7															
ethyl octanoate	8.7	2460						670	590	410	560	500	340	30.17	27.04	32.28	34.46
3-methylbutanal	0.50	119	254	117	145	147	287										
2-phenylethan-1-ol	140	28700						34000	51000	20000	47000	54000	33000	107270.06	134569.36	146411.10	143031.88
2-methylpropanal	0.49	36.5	25	28	28	40	133										
2-methylbutan-1-ol	1200	70100						60000	61000	33000	62000	53000	46000				
dimethyl sulfide	0.30	14.1															
acetic acid	5600	219000															
3-(methylsulfanyl)propan-1-ol	36	1360						590	450	140	610	590	310				
3-(methylsulfanyl)propanal	0.43	14.6	13.0	16	7.2	44	41										
ethyl propanoate	10	295															
octanoic acid	190	5580						2430	3290	3370	2170	2300	2320	854.27	913.68	1087.27	1181.95
3-hydroxybutan-2-one	590	16600						6000	3000	3000	5000	4000	3000	215.27	221.52	219.50	225.96
2-methylbutanal	1.5	40.2															
phenylacetic acid	68	452															
ethyl decanoate	122	741						90	250	130	90	210	10	91.83	91.19	91.81	114.00
decanoic acid	500	2460						450	1120	1090	380	810	700	183.69	185.22	189.44	189.80
hexan-1-ol	590	2710						1000	1200	1000	1100	900	800	5502.53	5576.00	9670.76	7590.31
phenylacetaldehyde	5.2	21.5	11.0	3.4	4.9	16	15.0										
2-phenylethyl acetate	360	682						140	310	70	110	240	60	126.92	116.55	175.35	71.91
2-methylpropan-1-ol	19000	33000						70000	33000	23000	56000	34000	27000				
3-methylbutanoic acid	490	814						1130	1700	870	1640	2190	2020				
2-methylpropyl acetate	66	101															
hexanoic acid	4800	4060						2980	2770	2570	2570	2620	1990				
benzaldehyde	150	108	45	5.6	2.7	9.6	26							98.85	304.07	136.91	163.13
butan-1-ol	1900	1120															
butanoic acid	2400	1180						1600	1490	1180	1650	1500	940	1516.75	1621.66	1835.97	1881.57
octan-1-ol	110	44.7												25.56	23.75	29.16	31.89
ethyl 2-phenylacetate	155.55	53.8												10.51	13.19	13.42	11.34
2-methylbutanoic acid	3100	545															
ethyl dodecanoate	3500	269												30.95	31.77	32.11	32.17
propanoic acid	20000	1490															
2-methylpropanoic acid	60000	2180						3200	2420		4910	3560	2300	4388.59	5425.47	5273.97	5677.87

		reference no.	113	113	113	113	113	113	113	113	113	113	113	113	113	113	113
		wine sample no.	376	377	378	379	380	381	382	383	384	385	386	387	388	389	390
matrix		mean															
ethanol (%, ALC/VOL)		12.9	11.6														
pH		3.4	3.46														
odorant	OTC (µg/kg) in water	mean concentration (µg/L)															
ethyl acetate	5	69100	19237.34	17864.94	19332.76	17720.38	17696.57	17637.44	16017.26	18324.82	17121.35	17527.55	17042.91	17167.77	16814.76	17080.08	17377.32
acetaldehyde	16	49100															
butane-2,3-dione	1.0	1400															
ethyl hexanoate	1.2	1570	1175.15	1234.83	1300.95	1187.21	1206.82	1229.32	1100.68	1258.43	1241.12	1282.22	1225.27	1212.25	1195.97	1271.53	1230.65
ethyl 3-methylbutanoate	0.023	27.5															
ethyl 2-methylpropanoate	0.089	93.5															
3-methylbutan-1-ol	220	172000	13526.02	13590.68	13874.43	13575.27	13419.62	13149.27	12121.52	13731.79	13600.81	13640.99	13626.73	13560.27	13437.51	13553.39	13704.47
3-methylbutyl acetate	7.2	3650															
ethyl butanoate	0.76	374	551.8	497.33	497.44	506.85	491.55	496.21	456.51	512.08	455.63	471.75	460.52	459.15	451.88	466.19	469.38
ethyl 2-methylbutanoate	0.13	42.7															
ethyl octanoate	8.7	2460	5123.02	5183.19	4478.78	5132.44	5057.4	5099.02	4086.47	5286.14	4832.02	5107.89	4779.62	4569.16	4358.63	4230.52	5310.55
3-methylbutanal	0.50	119															
2-phenylethan-1-ol	140	28700	3373.51	4469.91	4774.47	3718.27	4321.51	4418.1	2990.55	4085.3	3854.4	4346.33	3489.34	3667.33	3671.37	3492.24	3759.31
2-methylpropanal	0.49	36.5															
2-methylbutan-1-ol	1200	70100															
dimethyl sulfide	0.30	14.1															
acetic acid	5600	219000															
3-(methylsulfanyl)propan-1-ol	36	1360	23.99	26.38	29.54	25.04	26.83	26.93	21.48	25.77	23.66	25.44	22.14	24.18	23.29	22.47	23.28
3-(methylsulfanyl)propanal	0.43	14.6															
ethyl propanoate	10	295															
octanoic acid	190	5580	3476.86	3767.48	4029.52	3317.02	3670.39	3676.58	2925.77	3608.76	3221.48	3482.42	3138.77	3121.03	3074.97	2589.67	3545.4
3-hydroxybutan-2-one	590	16600															
2-methylbutanal	1.5	40.2															
phenylacetic acid	68	452															
ethyl decanoate	122	741	3710.56	2353.45	2974.05	2200.24	2636.51	2503.6	2392.81	2913.78	1793.44	1916.82	1796.77	1680.79	1507.3	1299.43	3332.16
decanoic acid	500	2460	1101.4	1081.03	1187.99	974.48	1070.73	1094.96	918.69	1175.04	887.8	940.18	857.47	818.04	713.37	629.38	1168.13
hexan-1-ol	590	2710	537.7	550.89	604.93	588.16	553.14	548.44	520.84	563.05	580.3	583.9	590.17	584.18	577.37	586.14	576.16
phenylacetaldehyde	5.2	21.5															
2-phenylethyl acetate	360	682	182.38	140.29	141.56	129.39	140.11	136.97	121.46	138.67	74.13	80.8	71.28	73.67	72.11	73.4	81.76
2-methylpropan-1-ol	19000	33000	6401.22	6312.44	6037.56	6280.64	6308.8	6187.14	5912.98	6575.1	6434.77	6357.6	6565.47	6307.33	6515.84	6317.63	6299.22
3-methylbutanoic acid	490	814															
2-methylpropyl acetate	66	101															
hexanoic acid	4800	4060	1610.8	1988.35	2000.62	1729.48	1828.16	1834.68	1455.42	1758.96	1714.06	1861.7	1657.6	1661.17	1700.46	1614.36	1662.99
benzaldehyde	150	108															
butan-1-ol	1900	1120															
butanoic acid	2400	1180															
octan-1-ol	110	44.7	1.27	1.42	1.63	1.49	1.41	1.35	1.26	1.46	1.42	1.46	1.39	1.36	1.3	1.44	1.38
ethyl 2-phenylacetate	155.55	53.8	0.5	0.63	0.7	0.6	0.62	0.59	0.53	0.6	0.83	0.9	0.84	0.84	0.8	0.81	0.86
2-methylbutanoic acid	3100	545															
ethyl dodecanoate	3500	269	408.5	213.14	248.01	188.35	227.36	197	220.09	311.27	133.94	124.27	104.08	98.96	74.89	64.88	311.45
propanoic acid	20000	1490															
2-methylpropanoic acid	60000	2180															

		reference no.	113	113	113	113	113	113	113	114	114	114	114	114	114	115	115
		wine sample no.	391	392	393	394	395	396	397	398	399	400	401	402	403	404	405
matrix		mean															
ethanol (%, ALC/VOL)		12.9															
pH		3.4															
odorant	OTC (µg/kg) in water	mean concentration (µg/L)															
ethyl acetate	5	69100	15779.01	15085.09	15882.21	15076.34	15396.32	15822.02	16263.98	59279	70279	53279	68279	50779	63279	38670	38340
acetaldehyde	16	49100														33800	47160
butane-2,3-dione	1.0	1400															
ethyl hexanoate	1.2	1570	1097.19	971.94	1089.06	993.7	1032.43	1088.04	1086.68	1094	1231	1182	1094	918	1418	444.37	508.13
ethyl 3-methylbutanoate	0.023	27.5								15.7	30.0	5.24	22.4	15.9	20.0	7.10	5.79
ethyl 2-methylpropanoate	0.089	93.5								115	141	42.3	121	90.6	77.8	8.68	4.76
3-methylbutan-1-ol	220	172000	13320.99	13134.23	13174.89	13115.28	12788.18	13399.32	13689.33	99317	103460	113317	104317	107603	109031	178870	178450
3-methylbutyl acetate	7.2	3650								394	380	376	405	376	376	1519.55	1639.48
ethyl butanoate	0.76	374	443.68	407.88	445.42	411.59	429.77	446.61	454.76	109	133	149	134	119	152	273.19	268.09
ethyl 2-methylbutanoate	0.13	42.7								72.6	109	27.1	87.3	69.9	63.9	3.64	3.68
ethyl octanoate	8.7	2460	2724.93	2542.83	2729.86	2401.3	2369.03	2278.57	2842.18	152	147	174	134	144	226	610.54	714.90
3-methylbutanal	0.50	119															
2-phenylethan-1-ol	140	28700	4048.79	3258.31	2966.21	3041.62	3205.98	3282.49	3276.59	18909	18509	17409	18709	20109	18709	25490	25990
2-methylpropanal	0.49	36.5															
2-methylbutan-1-ol	1200	70100															
dimethyl sulfide	0.30	14.1															
acetic acid	5600	219000								1147	1267	1207	1207	1001	1259		
3-(methylsulfanyl)propan-1-ol	36	1360	24.44	22.51	21.25	20.28	22.61	21.78	22.33								
3-(methylsulfanyl)propanal	0.43	14.6															
ethyl propanoate	10	295															
octanoic acid	190	5580	3153.55	3153.77	2962.12	3157.24	3067.82	2715.87	3620.3	376	304	492	249	323	510	4760	5280
3-hydroxybutan-2-one	590	16600															
2-methylbutanal	1.5	40.2															
phenylacetic acid	68	452															
ethyl decanoate	122	741	1233.31	1223.91	1197.39	1109	921.51	922.08	1597.87	41	40.3	62.8	44.2	41.7	53.8	255.86	334.62
decanoic acid	500	2460	811.49	770.08	728.91	791.33	683.44	562.25	1062.02	104	94.2	105	82.9	95.8	142	920	1300
hexan-1-ol	590	2710	580.99	600.41	595.35	598.65	582.17	613.22	613.47	4427	2252	3702	3452	3377	4327	299.34	584.23
phenylacetaldehyde	5.2	21.5															
2-phenylethyl acetate	360	682	46.41	51.79	48.67	51.28	50.1	52.36	60.64							2209.30	2303.58
2-methylpropan-1-ol	19000	33000	6261.86	6165.66	6174.79	6169.14	6107.16	6342.91	6374.4	43243	39910	42577	39243	43577	40910	18080	17510
3-methylbutanoic acid	490	814															
2-methylpropyl acetate	66	101								65.6	62.2	39.8	64.2	45.2	69.6	27.92	24.86
hexanoic acid	4800	4060	1595.72	1638.52	1535.19	1631.23	1623.13	1651.7	1724.47	857	847	990	770	722	1045	4700	4770
benzaldehyde	150	108								1.81	10.7	4.36	2.30	7.48	3.41		
butan-1-ol	1900	1120								1083	823	1243	1280	1167	957		
butanoic acid	2400	1180															
octan-1-ol	110	44.7	1.18	1.31	1.29	1.28	1.25	1.48	1.57	2.40	2.25	3.55	2.23	2.36	2.83		
ethyl 2-phenylacetate	155.55	53.8	0.72	0.77	0.78	0.81	0.75	0.78	0.86	4.01	5.61	2.28	4.54	3.89	5.11		
2-methylbutanoic acid	3100	545															
ethyl dodecanoate	3500	269	92.26	64.61	57.09	58.48	37.1	37.62	140.69	41	28.9	82.2	62.3	63.3	56.5		
propanoic acid	20000	1490															
2-methylpropanoic acid	60000	2180															

		reference no.	116	116	116	116	116	116	116	116	116	116	116	116	116	116	116
		wine sample no.	406	407	408	409	410	411	412	413	414	415	416	417	418	419	420
matrix		mean															
ethanol (%, ALC/VOL)		12.9	13	13.9	13.9	13.9	13.5	14.5	13.5	12.0	12.0	13.1	13.0	11.5	11.8	12.0	13.9
pH		3.4	3.62	3.60	3.56	3.56	3.48	3.55	3.59	3.40	3.29	3.31	3.24	3.05	3.26	3.01	3.53
odorant	OTC (µg/kg) in water	mean concentration (µg/L)															
ethyl acetate	5	69100															
acetaldehyde	16	49100															
butane-2,3-dione	1.0	1400															
ethyl hexanoate	1.2	1570															
ethyl 3-methylbutanoate	0.023	27.5															
ethyl 2-methylpropanoate	0.089	93.5															
3-methylbutan-1-ol	220	172000															
3-methylbutyl acetate	7.2	3650															
ethyl butanoate	0.76	374															
ethyl 2-methylbutanoate	0.13	42.7															
ethyl octanoate	8.7	2460															
3-methylbutanal	0.50	119	19	18	15	11	18.0	23	27	19	134	138	133	156	145	128	126
2-phenylethan-1-ol	140	28700															
2-methylpropanal	0.49	36.5	35	45	44	29	51	56	57	105	33	47	28	41	60	41	68
2-methylbutan-1-ol	1200	70100															
dimethyl sulfide	0.30	14.1															
acetic acid	5600	219000															
3-(methylsulfanyl)propan-1-ol	36	1360															
3-(methylsulfanyl)propanal	0.43	14.6	6.7	4.4	3.7	2.40	5.6	8.4	11.0	2.90	13	23.0	11.0	12.0	18	9.0	17
ethyl propanoate	10	295															
octanoic acid	190	5580															
3-hydroxybutan-2-one	590	16600															
2-methylbutanal	1.5	40.2															
phenylacetic acid	68	452															
ethyl decanoate	122	741															
decanoic acid	500	2460															
hexan-1-ol	590	2710															
phenylacetaldehyde	5.2	21.5	13.0	13.0	10	7.1	13	16.0	28.0	9.0	71	82	48	65	77	41	95
2-phenylethyl acetate	360	682															
2-methylpropan-1-ol	19000	33000															
3-methylbutanoic acid	490	814															
2-methylpropyl acetate	66	101															
hexanoic acid	4800	4060															
benzaldehyde	150	108	17	2.2	2.4	3.5	0.70	1.20	6.6	1.9	2.5	3.6	1.70	1.60	3.8	1.3	2.0
butan-1-ol	1900	1120															
butanoic acid	2400	1180															
octan-1-ol	110	44.7															
ethyl 2-phenylacetate	155.55	53.8															
2-methylbutanoic acid	3100	545															
ethyl dodecanoate	3500	269															
propanoic acid	20000	1490															
2-methylpropanoic acid	60000	2180															

		reference no.	116	117	117	117	117	117	117	117	117	117	117	117	117	117	117
		wine sample no.	421	422	423	424	425	426	427	428	429	430	431	432	433	434	435
matrix		mean															
ethanol (%, ALC/VOL)		12.9	13.0														
pH		3.4	3.20														
odorant	OTC (µg/kg) in water	mean concentration (µg/L)															
ethyl acetate	5	69100															
acetaldehyde	16	49100															
butane-2,3-dione	1.0	1400															
ethyl hexanoate	1.2	1570		1835		1547	1399			1570		1405	1310			1752	
ethyl 3-methylbutanoate	0.023	27.5															
ethyl 2-methylpropanoate	0.089	93.5		4.56		4.21	4.06			3.71		3.82	3.99			4.76	
3-methylbutan-1-ol	220	172000															
3-methylbutyl acetate	7.2	3650		6007		3973	3663			4979		3834	3513			6105	
ethyl butanoate	0.76	374		537		382	359			440		360	345			549	
ethyl 2-methylbutanoate	0.13	42.7		0.554		0.516	0.509			0.455		0.483	0.488			0.579	
ethyl octanoate	8.7	2460		1536		1396	1272			1263		1326	1190			1557	
3-methylbutanal	0.50	119	157	135.33	150.90	161.69	174.67	238.57	239.56	181.12	204.28	210.28	238.13	281.07	290.29	194.99	232.99
2-phenylethan-1-ol	140	28700															
2-methylpropanal	0.49	36.5	87	43.96	53.18	57.50	66.70	11.65	13.18	47.98	53.85	60.98	72.12	12.54	13.33	44.16	63.37
2-methylbutan-1-ol	1200	70100															
dimethyl sulfide	0.30	14.1		4.09	1.08	0.82	1.42	9.18	10.71	3.04	0.83	2.39	2.25	9.92	9.52	3.01	1.10
acetic acid	5600	219000															
3-(methylsulfanyl)propan-1-ol	36	1360															
3-(methylsulfanyl)propanal	0.43	14.6	21	5.28	5.89	3.73	6.35	35.31	31.09	5.22	4.00	5.27	7.63	29.24	32.89	3.46	4.33
ethyl propanoate	10	295		156			106			130		99	99			168	
octanoic acid	190	5580															
3-hydroxybutan-2-one	590	16600															
2-methylbutanal	1.5	40.2															
phenylacetic acid	68	452															
ethyl decanoate	122	741		8.0		6.7	6.7			6.2		7.5	5.6			6.7	
decanoic acid	500	2460															
hexan-1-ol	590	2710		23.2		27.1	23.2			21.8		21.7	24.3			23.2	
phenylacetaldehyde	5.2	21.5	64	3.45	3.55	4.33	4.62	8.83	7.72	4.53	4.52	5.36	5.85	10.36	9.49	5.30	5.60
2-phenylethyl acetate	360	682		531		569	500			437		530	470			449	
2-methylpropan-1-ol	19000	33000															
3-methylbutanoic acid	490	814															
2-methylpropyl acetate	66	101		109.1		68.1	63.6			97.4		64.5	59.0			109.1	
hexanoic acid	4800	4060															
benzaldehyde	150	108	20.0	1.29	2.02	2.47	2.03	3.35	3.86	1.21	1.91	2.91	2.39	4.55	3.51	1.23	2.08
butan-1-ol	1900	1120															
butanoic acid	2400	1180															
octan-1-ol	110	44.7															
ethyl 2-phenylacetate	155.55	53.8		0.412		0.297	0.328			0.336		0.313	0.281			0.419	
2-methylbutanoic acid	3100	545															
ethyl dodecanoate	3500	269		3079		1785	1581			2259		2068	1420			2589	
propanoic acid	20000	1490															
2-methylpropanoic acid	60000	2180															

		reference no.	117	117	117	117	117	117	117	117	117	117	117	117	117	118	118
		wine sample no.	436	437	438	439	440	441	442	443	444	445	446	447	448	449	450
matrix		mean															
ethanol (%, ALC/VOL)		12.9															
pH		3.4															
odorant	OTC (µg/kg) in water	mean concentration (µg/L)															
ethyl acetate	5	69100														32020	20140
acetaldehyde	16	49100															
butane-2,3-dione	1.0	1400															
ethyl hexanoate	1.2	1570	1418	1393			202	151		229	151		265	189		760	1060
ethyl 3-methylbutanoate	0.023	27.5															
ethyl 2-methylpropanoate	0.089	93.5	4.48	3.96			11.8	10.2		12.6	9.4		13.2	9.5			
3-methylbutan-1-ol	220	172000														96170	87050
3-methylbutyl acetate	7.2	3650	3650	3463			744	532		737	475		1030	627		3200	640
ethyl butanoate	0.76	374	357	352			104	76		127	82		158	103		300	240
ethyl 2-methylbutanoate	0.13	42.7	0.568	0.515			3.4	2.4		4.0	2.5		4.5	2.8		10	10
ethyl octanoate	8.7	2460	1356	1287			213	146		356	149		267	182		990	540
3-methylbutanal	0.50	119	230.91	256.02	301.67	330.59	274.01	238.56	258.15	336.54	233.34	239.86	340.27	225.10	220.76		
2-phenylethan-1-ol	140	28700														15840	41630
2-methylpropanal	0.49	36.5	62.66	84.58	11.55	13.95	4.40	2.13	6.55	4.43	1.83	5.76	4.13	2.03	5.12		
2-methylbutan-1-ol	1200	70100															
dimethyl sulfide	0.30	14.1	1.33	1.16	10.27	10.34	2.76	0.96	17.60	2.76	0.77	17.28	2.67	0.63	16.29		
acetic acid	5600	219000															
3-(methylsulfanyl)propan-1-ol	36	1360															
3-(methylsulfanyl)propanal	0.43	14.6	4.89	6.75	31.60	32.29	6.20	4.80	169.75	6.76	4.44	143.02	6.64	3.86	162.44		
ethyl propanoate	10	295	108	109			83	69		108	75		128	87			
octanoic acid	190	5580															
3-hydroxybutan-2-one	590	16600															
2-methylbutanal	1.5	40.2															
phenylacetic acid	68	452															
ethyl decanoate	122	741	8.2	5.7			0.639	0.260		1.429	0.325		1.067	0.460		300	60
decanoic acid	500	2460															
hexan-1-ol	590	2710	19.8	23.0			17.1	19.6		28.1	21.5		20.7	20.2		80	450
phenylacetaldehyde	5.2	21.5	6.49	6.59	9.97	10.22	2.62	1.56	8.86	3.16	2.05	6.69	3.24	1.52	6.56		
2-phenylethyl acetate	360	682	451	481			43.2	45.5		54.7	35.9		58.5	48.3		260	70
2-methylpropan-1-ol	19000	33000														24670	8580
3-methylbutanoic acid	490	814															
2-methylpropyl acetate	66	101	63.1	58.9			43.3	30.4		40.5	25.7		51.7	32.0		10	20
hexanoic acid	4800	4060															
benzaldehyde	150	108	2.33	2.43	4.99	3.74	6.84	7.53	1.53	8.06	8.19	1.54	7.43	8.10	1.86		
butan-1-ol	1900	1120														1980	620
butanoic acid	2400	1180															
octan-1-ol	110	44.7														10	10
ethyl 2-phenylacetate	155.55	53.8	0.394	0.295			1.21	1.00		0.94	0.81		0.88	0.75			
2-methylbutanoic acid	3100	545															
ethyl dodecanoate	3500	269	2712	1474			114	97		220	98		203	121		140	100
propanoic acid	20000	1490															
2-methylpropanoic acid	60000	2180															

		reference no.	119	119	119	119	120	121	121	122	122	123	123	123	123	123	123
		wine sample no.	451	452	453	454	455	456	457	458	459	460	461	462	463	464	465
matrix		mean															
ethanol (%, ALC/VOL)		12.9															
pH		3.4															
odorant	OTC (µg/kg) in water	mean concentration (µg/L)															
ethyl acetate	5	69100					13943	12686	25636	82400	63821						
acetaldehyde	16	49100															
butane-2,3-dione	1.0	1400															
ethyl hexanoate	1.2	1570	450	570	300	310	721	830	263	1060	1119						
ethyl 3-methylbutanoate	0.023	27.5					104	184	87	27	37						
ethyl 2-methylpropanoate	0.089	93.5						111	67	70	104						
3-methylbutan-1-ol	220	172000					181159	107081	103010	106758	122317						
3-methylbutyl acetate	7.2	3650	730	1000	460	670	759	718	441	1107	968						
ethyl butanoate	0.76	374					1060	468	335	537	454						
ethyl 2-methylbutanoate	0.13	42.7					56	72	27	12	15						
ethyl octanoate	8.7	2460	780	950	340	410	458	1215	121	2880	2901						
3-methylbutanal	0.50	119										12.20	3.81	12.80	27.50	64.00	99.50
2-phenylethan-1-ol	140	28700	18300	19000	18800	21500	30893	21942	34704	15951	20318						
2-methylpropanal	0.49	36.5								45	15	88.90	56.00	72.80	91.00	101.00	114.00
2-methylbutan-1-ol	1200	70100								42989	48667						
dimethyl sulfide	0.30	14.1								20	29						
acetic acid	5600	219000								341015	238271						
3-(methylsulfanyl)propan-1-ol	36	1360								701	1850	466.0	494.0	470.0	470.0	471.00	351
3-(methylsulfanyl)propanal	0.43	14.6								0.7	0.2	4.32	1.09	3.77	4.87	18.30	22.80
ethyl propanoate	10	295					204	223	210	206	208						
octanoic acid	190	5580	1620	2450	1880	2490	1207	6347	982	9070	9254						
3-hydroxybutan-2-one	590	16600								300							
2-methylbutanal	1.5	40.2															
phenylacetic acid	68	452															
ethyl decanoate	122	741	250	300	84	110	65	443	12	676	561						
decanoic acid	500	2460	650	970	690	660	138	2073	179	2774	2964						
hexan-1-ol	590	2710	1880	1480	1960	1720		483	6609	1824	1768						
phenylacetaldehyde	5.2	21.5								12	5	4.73	2.880	9.16	12.60	26.50	18.80
2-phenylethyl acetate	360	682	120	180	85	120	48	237	606	76	83						
2-methylpropan-1-ol	19000	33000					13682	3494	8335	25807	23347						
3-methylbutanoic acid	490	814															
2-methylpropyl acetate	66	101					98	17	58	52	24						
hexanoic acid	4800	4060	3790	5080	3590	4960	929	1394	602	5493	5747						
benzaldehyde	150	108	4.87	5.30	5.69	5.55				119	36	7.2	18.8	25.6	24.5	7.8	23.0
butan-1-ol	1900	1120					2059	1081	726	1016	838						
butanoic acid	2400	1180	32.7	30.9	34.1	37.1											
octan-1-ol	110	44.7					23	16	13								
ethyl 2-phenylacetate	155.55	53.8	1.11	0.96	2.42	2.24											
2-methylbutanoic acid	3100	545															
ethyl dodecanoate	3500	269					1			74	54						
propanoic acid	20000	1490								893	759						
2-methylpropanoic acid	60000	2180								616	646						

		reference no.	124	125	125	125	125	126	126	126	126	126	126	126	127	128	128
		wine sample no.	466	467	468	469	470	471	472	473	474	475	476	477	478	479	480
matrix		mean															
ethanol (%, ALC/VOL)		12.9														11.9	12.0
pH		3.4														3.3	3.1
odorant	OTC (µg/kg) in water	mean concentration (µg/L)															
ethyl acetate	5	69100														44870.3	40599.4
acetaldehyde	16	49100															
butane-2,3-dione	1.0	1400															
ethyl hexanoate	1.2	1570	1016	927.3	772.5	1034.9	867.9	2630	1810	1160	805	1330	1020	809	51	60.5	322.3
ethyl 3-methylbutanoate	0.023	27.5	3.47														
ethyl 2-methylpropanoate	0.089	93.5	16.5														
3-methylbutan-1-ol	220	172000													94469	40796.2	49988.3
3-methylbutyl acetate	7.2	3650	5794	444.4	563.9	1535.1	2139.9	1670	1530	1130	874	1200	1150	520		6549.8	939.0
ethyl butanoate	0.76	374	586					282	272	178	164	236	190	108			
ethyl 2-methylbutanoate	0.13	42.7	1.76														
ethyl octanoate	8.7	2460	1950	1235.7	1092.7	1389.4	1275.4	3570	1490	1510	1010	1240	1520	1010	42	651.2	7608.9
3-methylbutanal	0.50	119															
2-phenylethan-1-ol	140	28700						16600	16200	13200	91300	16200	13000	6570	19988	29477.5	12140.8
2-methylpropanal	0.49	36.5															
2-methylbutan-1-ol	1200	70100															
dimethyl sulfide	0.30	14.1															
acetic acid	5600	219000														36735.7	25325.5
3-(methylsulfanyl)propan-1-ol	36	1360														1918.8	1487.0
3-(methylsulfanyl)propanal	0.43	14.6															
ethyl propanoate	10	295	76.2														
octanoic acid	190	5580													990	798.7	216.2
3-hydroxybutan-2-one	590	16600															
2-methylbutanal	1.5	40.2															
phenylacetic acid	68	452															
ethyl decanoate	122	741	555	490.6	576.2	774.5	723.6								10	127.5	28.8
decanoic acid	500	2460															
hexan-1-ol	590	2710													742	2079.5	2516.2
phenylacetaldehyde	5.2	21.5														5.4	
2-phenylethyl acetate	360	682	297					385	230	227	213	228	236	170			5.8
2-methylpropan-1-ol	19000	33000													3704	44341.9	56007.6
3-methylbutanoic acid	490	814													685		
2-methylpropyl acetate	66	101	86.4														
hexanoic acid	4800	4060													904	1033.5	445.4
benzaldehyde	150	108															1.7
butan-1-ol	1900	1120													138	1151.8	791.2
butanoic acid	2400	1180													322		
octan-1-ol	110	44.7														13.3	
ethyl 2-phenylacetate	155.55	53.8	0.31														
2-methylbutanoic acid	3100	545															
ethyl dodecanoate	3500	269	136	25.97	22.87	30.11	33.94										
propanoic acid	20000	1490															
2-methylpropanoic acid	60000	2180														18.2	

		reference no.	128	128	128	129	129	130	131	131	131	132	132	133	133	133	133
		wine sample no.	481	482	483	484	485	486	487	488	489	490	491	492	493	494	495
matrix		mean															
ethanol (%, ALC/VOL)		12.9	11.9	12.2	11.8			14.37									
pH		3.4	3.2	3.0	3.1												
odorant	OTC (µg/kg) in water	mean concentration (µg/L)															
ethyl acetate	5	69100	82203.7	116484.0	20847.3	39510	19100	36110	44700	26000	66200			211800	177300	146300	211800
acetaldehyde	16	49100				14190	17040	27670	5610	7040	640						
butane-2,3-dione	1.0	1400				610	160										
ethyl hexanoate	1.2	1570	1690.5	1006.6	1253.5	230	120	6200	152	146	120			7200	10300	4100	12900
ethyl 3-methylbutanoate	0.023	27.5							2.72	1.70	2.97						
ethyl 2-methylpropanoate	0.089	93.5				20	10	30	21.5	13.4	13.7						
3-methylbutan-1-ol	220	172000	45280.1	49266.5	43648.7			74810	167000	224000	223000	202.78	275.11	1444000	1430000	1390000	1520000
3-methylbutyl acetate	7.2	3650	4181.3	2375.4	1260.7	840	420	1640	187	147	254			7900	8700	5800	15900
ethyl butanoate	0.76	374				240	90	980	167	56.8	133			180	150	140	200
ethyl 2-methylbutanoate	0.13	42.7															
ethyl octanoate	8.7	2460	125.8	2260.4	874.9	140	60	1180	340	214	162						28500
3-methylbutanal	0.50	119															
2-phenylethan-1-ol	140	28700	69528.7	99956.8	8289.0	23450	5580	42520	3110	3150	3300	49.57	53.71	24700	16500	17700	15800
2-methylpropanal	0.49	36.5															
2-methylbutan-1-ol	1200	70100															
dimethyl sulfide	0.30	14.1							11.7		39.3						
acetic acid	5600	219000	16057.9	28340.6	47398.2												
3-(methylsulfanyl)propan-1-ol	36	1360	2182.4	3032.6	1260.7	710	90										
3-(methylsulfanyl)propanal	0.43	14.6															
ethyl propanoate	10	295				130	20										
octanoic acid	190	5580	537.2	1281.8	394.4	1120	330	1750	418	282	379			17200	18900	10700	37400
3-hydroxybutan-2-one	590	16600				3560	170	10050				0.88	1.25				
2-methylbutanal	1.5	40.2															
phenylacetic acid	68	452															
ethyl decanoate	122	741		545.9	55.1			4010						599	270	110	217
decanoic acid	500	2460		269.2	497.5	180	30	1050	68.6	36.5	60.2			27200	36200	12100	64900
hexan-1-ol	590	2710	3271.7	3608.9	3051.7	2220	330	790	442	620	769			58100	55400	55000	27500
phenylacetaldehyde	5.2	21.5	20.6	26.6													
2-phenylethyl acetate	360	682	315.9	300.9	32.5	50	20	2600	2.88	2.37	3.71	11.59	11.41	11400	11200	10700	11200
2-methylpropan-1-ol	19000	33000	50914.1	52203.0	75637.9	45660	10700	34350	75700	95600	104000	9.25	16.16	1127500	742300	346800	
3-methylbutanoic acid	490	814						640									
2-methylpropyl acetate	66	101							50.9	23.7	12.2						
hexanoic acid	4800	4060	1564.7	2124.7	572.9	1220	50	3100	561	194	264			30800	12700	10400	19800
benzaldehyde	150	108	3.2	0.4								1.87	2.97				
butan-1-ol	1900	1120	944.1	897.6	709.3	1600	420	560									
butanoic acid	2400	1180															
octan-1-ol	110	44.7	29.2	5.2	17.5				343	293	413						132
ethyl 2-phenylacetate	155.55	53.8							0.15	0.33	0.21				506	501	
2-methylbutanoic acid	3100	545															
ethyl dodecanoate	3500	269															
propanoic acid	20000	1490	27.2	37.3													
2-methylpropanoic acid	60000	2180	33.4		7.5	2230	60	1690									

		reference no.	133	133	133	133	133	133	133	133	134	135	136	137	138	138	138
		wine sample no.	496	497	498	499	500	501	502	503	504	505	506	507	508	509	510
matrix		mean															
ethanol (%, ALC/VOL)		12.9									12.3	14.5	11.78	14.53			
pH		3.4									3.50	3.82		3.51			
odorant	OTC (µg/kg) in water	mean concentration (µg/L)															
ethyl acetate	5	69100	177300	146300	145400	138700	214300	155100	150200	121500	9.7		27793	21365.23			
acetaldehyde	16	49100											10001	332.27	8000	10500	14000
butane-2,3-dione	1.0	1400												1243.45		1300	900
ethyl hexanoate	1.2	1570	10800	44900	6600	1800	5400	8800	8100	6600	73	320	603	613.67			
ethyl 3-methylbutanoate	0.023	27.5									3.4		2.86				
ethyl 2-methylpropanoate	0.089	93.5											12.0				
3-methylbutan-1-ol	220	172000	1635000	4880000	1316600	1233400	1446800	1146900	1413700	1271100	26327	890	220696	391316.33			
3-methylbutyl acetate	7.2	3650	9900	19000	7700	5700	7000	11000	11400	16500	56	470	2017				
ethyl butanoate	0.76	374	180	370	150	140	150	80	90	160	32	130	149	157.33			
ethyl 2-methylbutanoate	0.13	42.7									2.9		1.17				
ethyl octanoate	8.7	2460	24000	22800	24100	6900	17600	13700	12300	10100	52	67	703	372.00			
3-methylbutanal	0.50	119											10.2				
2-phenylethan-1-ol	140	28700	16200	25500	16700	16700	17900	14900	16000	16200	4759	18860	35182	54308.67			
2-methylpropanal	0.49	36.5											6.4				
2-methylbutan-1-ol	1200	70100															
dimethyl sulfide	0.30	14.1															
acetic acid	5600	219000											300440	310000			
3-(methylsulfanyl)propan-1-ol	36	1360									48		1589				
3-(methylsulfanyl)propanal	0.43	14.6											2.2				
ethyl propanoate	10	295										100					
octanoic acid	190	5580	28900	98900	26000	11000	18000	34700	30400	19800	187		9522	9919.33			
3-hydroxybutan-2-one	590	16600											511				
2-methylbutanal	1.5	40.2											2.7				
phenylacetic acid	68	452															
ethyl decanoate	122	741	196	912	223	72	394	94	85	66	2.9	13	51.6	86.33	580	1080	1700
decanoic acid	500	2460	54400	18200	33800	12900	39700	56700	49600	17900	54		1081				
hexan-1-ol	590	2710	27600	32100	55800	55200	27300	27800	27600	54900	376	1360	548	1835.33			
phenylacetaldehyde	5.2	21.5											13.0		6.5	8	4.0
2-phenylethyl acetate	360	682	10900	12200	10800	10700	10800	10800	11100	11100	8	46	289	8.00	860	560	670
2-methylpropan-1-ol	19000	33000					757600			351400	949		25994	55502.00			
3-methylbutanoic acid	490	814												385.67	65	80	85
2-methylpropyl acetate	66	101									0.27		14.2				
hexanoic acid	4800	4060	16000	63700	14900	9900	11600	18900	15300	11700	203		3210	3614.33			
benzaldehyde	150	108		9600						884			26.6		8.1	6.1	6.1
butan-1-ol	1900	1120									21	1060	460				
butanoic acid	2400	1180											658		410		800
octan-1-ol	110	44.7	204	327							1.0						
ethyl 2-phenylacetate	155.55	53.8		432	500	493	506			242							
2-methylbutanoic acid	3100	545											1125		23	29	21
ethyl dodecanoate	3500	269									0.5	2.5					
propanoic acid	20000	1490															
2-methylpropanoic acid	60000	2180											1370	3614.33			

		reference no.	139	139	140	141	141	142	143	143	143	143	144	145	145	146	146
		wine sample no.	511	512	513	514	515	516	517	518	519	520	521	522	523	524	525
matrix		mean															
ethanol (%, ALC/VOL)		12.9	10.05	11.2		12.40	13.27							11.53	11.24		
pH		3.4	3.3	3.2										3.34	3.37	3.75	3.74
odorant	OTC (µg/kg) in water	mean concentration (µg/L)															
ethyl acetate	5	69100	53056.0	23688.5				90910	20000	21000	36000	33000	6365	34690	36750	670000	814000
acetaldehyde	16	49100						49780									
butane-2,3-dione	1.0	1400															
ethyl hexanoate	1.2	1570	638.2	714.4	158	1234	2600	82.45	288	293	80	92	5017	269.67	254.98		
ethyl 3-methylbutanoate	0.023	27.5				87		12.14	5.9	6.4	4.1	4.3	188			4.5	3.9
ethyl 2-methylpropanoate	0.089	93.5						4.44	125	125	149	151	41			7.30	7.98
3-methylbutan-1-ol	220	172000	27075.7	104872.7	240847	187000	339000	227000	188000	195000	141000	145000	105882	17534.81	15862.23	190000	161000
3-methylbutyl acetate	7.2	3650		281.4	652	172	774	420.81	177	180	141	155	582	1420.01	1158.54	457	393
ethyl butanoate	0.76	374				215	593	154.39	139	144	89	111	474	89.72	93.11	865	843
ethyl 2-methylbutanoate	0.13	42.7				49		10.13	5.3	5.8	2.9	3.2	95				
ethyl octanoate	8.7	2460	1294.3		249	490	1200	84.19	295	288	244	301	994	262.02	209.40		
3-methylbutanal	0.50	119															
2-phenylethan-1-ol	140	28700	22128.4	12471.2	80960	22000	15000	30230	35000	34000	33000	40000	65337	6103.51	5046.66	8172	5198
2-methylpropanal	0.49	36.5															
2-methylbutan-1-ol	1200	70100															
dimethyl sulfide	0.30	14.1															
acetic acid	5600	219000	31412.0	22524.6													
3-(methylsulfanyl)propan-1-ol	36	1360	19.1	13.3												242	331
3-(methylsulfanyl)propanal	0.43	14.6															
ethyl propanoate	10	295						0.97	57	55	49	53	32				
octanoic acid	190	5580	1442.4	780.5				661.83	3393	3345	2299	2407	6251	4066.26	2534.93	8000	7100
3-hydroxybutan-2-one	590	16600															
2-methylbutanal	1.5	40.2															
phenylacetic acid	68	452															
ethyl decanoate	122	741	115.4	22.3	35.7	105	180	113.13	72	69	67	75	278	113.54	55.76		
decanoic acid	500	2460	32.7	155.6				135.48	330	318	401	433	1992	809.74	292.23		
hexan-1-ol	590	2710	1439.7	10457.9	2146	4358	5300	1159.23	2251	2217	2876	2503	2325	453.33	479.73	3373	1969
phenylacetaldehyde	5.2	21.5															
2-phenylethyl acetate	360	682	30.7	2225.1				350.27	31	29	31	39	463	159.85	127.84		
2-methylpropan-1-ol	19000	33000	40123.1	16335.6				79740	119000	118000	63000	66000	3574	110.33	71.19	28016	26425
3-methylbutanoic acid	490	814															
2-methylpropyl acetate	66	101						66.41	117	118	120	126	20				
hexanoic acid	4800	4060	1480.8	1238.3				910.40	186	194	322	324	1280	1790.08	1750.20	8500	7800
benzaldehyde	150	108		495.7				4.71									
butan-1-ol	1900	1120	485.9	19841.5													
butanoic acid	2400	1180															
octan-1-ol	110	44.7	10.6	120.3				1.63	31	30	23	23	16				
ethyl 2-phenylacetate	155.55	53.8						16.38	2.2	2.0	1.9	2.6					
2-methylbutanoic acid	3100	545															
ethyl dodecanoate	3500	269		186.3				9.67					17				
propanoic acid	20000	1490	4234.0														
2-methylpropanoic acid	60000	2180	116.2														

		reference no.	146	146	146	146	147	148	149	150	150	150	150	151	151	152	152
		wine sample no.	526	527	528	529	530	531	532	533	534	535	536	537	538	539	540
matrix		mean															
ethanol (%, ALC/VOL)		12.9						12.4		12.6	12.7	15.0	14.9	14.2	13.5		
pH		3.4	3.82	3.64	3.67	3.67		3.45		3.50	3.48	3.45	3.48	3.25	3.20		
odorant	OTC (µg/kg) in water	mean concentration (µg/L)															
ethyl acetate	5	69100	682000	860000	987000	596000		31800	6365	61900	53900	46800	47300	36000	22100		
acetaldehyde	16	49100						73300				580	960	49300	204000		
butane-2,3-dione	1.0	1400															
ethyl hexanoate	1.2	1570					737.62	227	853	450	540	730	1010			500	190
ethyl 3-methylbutanoate	0.023	27.5	4.2	5.2	4.4	4.2	3.25			6.58	5.74	4.25	4.32				
ethyl 2-methylpropanoate	0.089	93.5	7.27	10.03	7.63	7.60	11.50					10.3	12.7			870	700
3-methylbutan-1-ol	220	172000	182000	205000	170000	187000	112701.7	161000	105882	168000	184000	132000	136000				
3-methylbutyl acetate	7.2	3650	358	448	409	325	3175.57	1375	582	3290	3600	5800	5530			1060	870
ethyl butanoate	0.76	374	800	900	1058	801	463.89		474	580	570	450	440			880	760
ethyl 2-methylbutanoate	0.13	42.7					1.48			0.69	0.92	0.66	0.26				
ethyl octanoate	8.7	2460					731.47	574	1294	380	490	510	1170				
3-methylbutanal	0.50	119															
2-phenylethan-1-ol	140	28700	6858	9506	10044	16427	1361	2778	65337	14500	15400	13800	14200	85000	71000	46280	60000
2-methylpropanal	0.49	36.5															
2-methylbutan-1-ol	1200	70100						47700									
dimethyl sulfide	0.30	14.1															
acetic acid	5600	219000						5.08		299000	240000	423000	464000			2200	17800
3-(methylsulfanyl)propan-1-ol	36	1360	325	406	234	359	1936.85	209		630	670	680	800			210	480
3-(methylsulfanyl)propanal	0.43	14.6					98.09										
ethyl propanoate	10	295														810	720
octanoic acid	190	5580	8400	6700	8000	8300	680	303	6251	7890	5860	7400	9350			4500	4000
3-hydroxybutan-2-one	590	16600									1210	610	810	234000	176000	14200	17900
2-methylbutanal	1.5	40.2															
phenylacetic acid	68	452															
ethyl decanoate	122	741					318.42	192	478			70	180				
decanoic acid	500	2460					20187.46	292	1992	520	490	1150	2800				
hexan-1-ol	590	2710	3473	4267	2295	3963	2499.60	911	465	1990	2020	1260	1420			3990	7400
phenylacetaldehyde	5.2	21.5															
2-phenylethyl acetate	360	682					511.13	152	463	228	222	315	313				
2-methylpropan-1-ol	19000	33000	28303	30891	26112	23263	17840	38800	3574	29400	31500	21900	22200	47200	46000	14400	12000
3-methylbutanoic acid	490	814					460.23	187		650	720	770	760				
2-methylpropyl acetate	66	101					47.41			53.5	59.0	85.7	79.6				
hexanoic acid	4800	4060	8200	8800	8000	8600	5094.66	241	1280	4620	4110	3840	4340			5500	4800
benzaldehyde	150	108					0.05	8.88		6.39	3.63	6.10	5.91			600	390
butan-1-ol	1900	1120					583.82	3.36	881	770	660	1340	1210				
butanoic acid	2400	1180						11.8		1420	1470	980	980			2830	3000
octan-1-ol	110	44.7															
ethyl 2-phenylacetate	155.55	53.8					4.15										
2-methylbutanoic acid	3100	545															
ethyl dodecanoate	3500	269					39.63		17								
propanoic acid	20000	1490						0.55									
2-methylpropanoic acid	60000	2180					925.87	223	62	580	490	950	970				4400

		reference no.	152	152	152	152	152	152	152	152	153	153	154	154	154	154	154
		wine sample no.	541	542	543	544	545	546	547	548	549	550	551	552	553	554	555
matrix		mean															
ethanol (%, ALC/VOL)		12.9									14.1	13.125	12.63	13.41	13.10	12.88	12.54
pH		3.4									3.65	3.29	3.30	3.29	3.31	3.30	3.30
odorant	OTC (µg/kg) in water	mean concentration (µg/L)															
ethyl acetate	5	69100											112922	102219	117548	105940	119178
acetaldehyde	16	49100									1382	1473					
butane-2,3-dione	1.0	1400									2066	300					
ethyl hexanoate	1.2	1570	700	600	700	500	1010	800	1400	1600			2882	2611	2699	2931	3200
ethyl 3-methylbutanoate	0.023	27.5											4.137	3.595	3.294	3.655	4.145
ethyl 2-methylpropanoate	0.089	93.5	880	770	700		990						18.7	13.0	13.7	11.2	12.1
3-methylbutan-1-ol	220	172000									199000	148000	179457	170733	185194	168891	183559
3-methylbutyl acetate	7.2	3650	920	1500	1650	1750	1070	920	850	1310			15411	12655	15267	13376	16028
ethyl butanoate	0.76	374	1130	830	800	1000	1250	810		980			1110.0	961.9	989.5	935.1	1071.9
ethyl 2-methylbutanoate	0.13	42.7															
ethyl octanoate	8.7	2460	30			30	10	20					2216	2019	2336	3171	3352
3-methylbutanal	0.50	119									10.8	6.91					
2-phenylethan-1-ol	140	28700	48450	46800	30000	49500	40600	43600	40000	28600	24100	70500	11603	12177	9756	5790	6391
2-methylpropanal	0.49	36.5									17.9	7.08					
2-methylbutan-1-ol	1200	70100															
dimethyl sulfide	0.30	14.1															
acetic acid	5600	219000	12300	10700	7800	5500	5800	9800	16200	16900							
3-(methylsulfanyl)propan-1-ol	36	1360	300	120	500	60	50	200		70	1680	1020	2190	2093	2274	1996	2152
3-(methylsulfanyl)propanal	0.43	14.6									7.45	3.78					
ethyl propanoate	10	295	790	660	800	1000	1340		660								
octanoic acid	190	5580	7500	4930	3700	7400	6200	4800		5400			7600	7800	8500	8400	8700
3-hydroxybutan-2-one	590	16600	8220	1600		3700		6400	15500	19800							
2-methylbutanal	1.5	40.2									6.80	2.24					
phenylacetic acid	68	452															
ethyl decanoate	122	741											566	503	722	1292	1287
decanoic acid	500	2460				3800	3450		2820				8300	9400	8400	11700	12300
hexan-1-ol	590	2710	5110	5300	5900	3700	3820	3500		1980			2235	1997	2283	2218	2307
phenylacetaldehyde	5.2	21.5									18.7	8.13					
2-phenylethyl acetate	360	682											455.6	448	486.1	460.8	500.5
2-methylpropan-1-ol	19000	33000	12200	8200	13300	12600	14900	11700	10700	10030	42000	22800	19820	19052	21201	19207	20645
3-methylbutanoic acid	490	814															
2-methylpropyl acetate	66	101											250.3	215.3	230.9	206.0	244.1
hexanoic acid	4800	4060	9250	5900	4200	6600	5100	5050	6900	5200			4000	3960	4660	4250	4490
benzaldehyde	150	108	500	600	580	500	600	400	400	390	6.04	29.5					
butan-1-ol	1900	1120											1590	1515	1673	1688	1763
butanoic acid	2400	1180	3530	3190	2870	7900	3280	2770	2780	3000							
octan-1-ol	110	44.7															
ethyl 2-phenylacetate	155.55	53.8															
2-methylbutanoic acid	3100	545															
ethyl dodecanoate	3500	269											55.4	77.1	70.9	122.9	113.8
propanoic acid	20000	1490															
2-methylpropanoic acid	60000	2180	3500	3400	3100	2900	3200	3500	2950	4600							

		reference no.	155	156	156	157	157	157	157	157	157	158	158	158	158	158	158
		wine sample no.	556	557	558	559	560	561	562	563	564	565	566	567	568	569	570
matrix		mean															
ethanol (%, ALC/VOL)		12.9		12.7	13.0							12.4	13.1	12.5	13.3	13.5	11.3
pH		3.4										3.41	3.15	3.37	3.37	3.64	3.33
odorant	OTC (µg/kg) in water	mean concentration (µg/L)															
ethyl acetate	5	69100	20571	100372.34	23025.19	87000	81000	82000	82000	73000	83000	222336	165703	191277	225800	207697	228966
acetaldehyde	16	49100				43000	42000	41000	40000	41000	41000						
butane-2,3-dione	1.0	1400															
ethyl hexanoate	1.2	1570	195	418.98	146.53	1500	1500	1500	1500	1500	1500	728	1885	1347	1666	121	1592
ethyl 3-methylbutanoate	0.023	27.5			10.45							91.3	68.0	61.8	105	68.5	27.6
ethyl 2-methylpropanoate	0.089	93.5			49.29							17.9		33.1	53.1	29.7	7.8
3-methylbutan-1-ol	220	172000	129750		2661.08	162000	155000	161000	160000	150000	158000	77901	100580	135207	125713	141238	89678
3-methylbutyl acetate	7.2	3650	824	156.25	109.96	7500	6400	5600	5100	4800	4700		1987		2773	131	6366
ethyl butanoate	0.76	374	1233	649.57	449.73	570	550	550	530	510	540	147	560	527	442		448
ethyl 2-methylbutanoate	0.13	42.7															
ethyl octanoate	8.7	2460	479	147.60	19.23	1400	1900	1600	1600	1600	1400	2493	6787	1870	4796	1652	3787
3-methylbutanal	0.50	119															
2-phenylethan-1-ol	140	28700	70556	52379.56	14640.80	14000	13000	13000	14000	13000	13000	5583	7361	6075	7856	9244	4584
2-methylpropanal	0.49	36.5															
2-methylbutan-1-ol	1200	70100															
dimethyl sulfide	0.30	14.1															
acetic acid	5600	219000				447000	415000	438000	434000	371000	429000	188030	141728	113475	143275	300041	108772
3-(methylsulfanyl)propan-1-ol	36	1360	50														
3-(methylsulfanyl)propanal	0.43	14.6															
ethyl propanoate	10	295															
octanoic acid	190	5580	5060	12772.54	2985.98	8000	7300	7800	7900	7800	7900	2134	5094		3241		3679
3-hydroxybutan-2-one	590	16600															
2-methylbutanal	1.5	40.2															
phenylacetic acid	68	452															
ethyl decanoate	122	741	31		16.65	2100	3900	2600	2500	2300	1600	2298	5106	610	3931	1512	3867
decanoic acid	500	2460		4943.49	1211.42	3000	2300	2500	2500	2500	2700	1858	2246	76.7	1965	698	3049
hexan-1-ol	590	2710	8919	11055.22	104.82	910	910	970	990	960	990	9296	4599	12269	5819	4249	8408
phenylacetaldehyde	5.2	21.5															
2-phenylethyl acetate	360	682		294.48	18.41	2700	2400	2300	2200	2100	2000	28.6	240		113	8.2	199
2-methylpropan-1-ol	19000	33000	30047	44933.06		19000	18000	19000	18000	16000	18000	2296	1983	4753	2948	12471	2089
3-methylbutanoic acid	490	814				630	580	600	610	580	600						
2-methylpropyl acetate	66	101	172		18.69												
hexanoic acid	4800	4060	873	4146.54	514.19	5500	5200	5400	5400	5300	5300	6570	5505	143	1781		2429
benzaldehyde	150	108			3.24							74.0				27.7	
butan-1-ol	1900	1120	1878			1700	1600	1700	1600	1400	1600						
butanoic acid	2400	1180				290	280	290	300	280	290						
octan-1-ol	110	44.7										38.5	43.6	42.1	46.6	36.6	9.6
ethyl 2-phenylacetate	155.55	53.8			4.36							35.8	4.7	1.9	5.7	4.3	0.56
2-methylbutanoic acid	3100	545															
ethyl dodecanoate	3500	269	7									31.4	55.5		89.3	96.5	119
propanoic acid	20000	1490				1900	1700	1900	1900	1700	1900						
2-methylpropanoic acid	60000	2180	14			780	730	760	760	700	740						

		reference no.	158	158	158	158	158	158	158	158	158	158	158	158	158	158	158
		wine sample no.	571	572	573	574	575	576	577	578	579	580	581	582	583	584	585
matrix		mean															
ethanol (%, ALC/VOL)		12.9	12.5	13.2	13.1	13.1	12.7	13.1	11.7	13.5	13.5	13.0	12.9	13.2	11.5	13.4	12.4
pH		3.4	3.37	3.36	3.21	3.43	3.37	3.23	3.23	3.65	3.41	3.29	3.52	3.39	3.25	3.14	3.05
odorant	OTC (µg/kg) in water	mean concentration (µg/L)															
ethyl acetate	5	69100	202084	194655	228361	241265	236165	185071	139539	160838	209868	133760	175743	74639	129241	265274	74978
acetaldehyde	16	49100															
butane-2,3-dione	1.0	1400															
ethyl hexanoate	1.2	1570	1221	1714	957	1396	1083	276	693	318	1127	563	435	375	366	543	13.1
ethyl 3-methylbutanoate	0.023	27.5	39.1	64.3	38.2	42.9	28.6	395	74.5	7.7	67.0	91.7	83.2	192	87.7	143	151
ethyl 2-methylpropanoate	0.089	93.5	12.5					178						117	25.9	117	41.8
3-methylbutan-1-ol	220	172000	99575	99541	124773	93668	90976	156560	65685	111889	100901	115507	102515	114513	79523	123811	83625
3-methylbutyl acetate	7.2	3650	4751	3716	8131	4007	8523		1632	5907	1791	1243	3036		1045		
ethyl butanoate	0.76	374	369	431	503	282	357	233	243	242	272	73.4	161	129	87.7	60.4	9.9
ethyl 2-methylbutanoate	0.13	42.7															
ethyl octanoate	8.7	2460	3798	5676	2492	4793	3407	936	2773	1734	3261	1698	1549	1322	1971	1901	216
3-methylbutanal	0.50	119															
2-phenylethan-1-ol	140	28700	9380	4753	4362	5480	7255	12984	2972	8000	6534	8183	7340	9652	3751	10472	4140
2-methylpropanal	0.49	36.5															
2-methylbutan-1-ol	1200	70100															
dimethyl sulfide	0.30	14.1															
acetic acid	5600	219000	99387	184611	134907	221622	111887	173441	115305	210659	159339	114571	139010	115197	157679	235282	129655
3-(methylsulfanyl)propan-1-ol	36	1360															
3-(methylsulfanyl)propanal	0.43	14.6															
ethyl propanoate	10	295															
octanoic acid	190	5580	3672	4266	800	3588	2137		2083	803	1623	1955	2125	980	1360		
3-hydroxybutan-2-one	590	16600															
2-methylbutanal	1.5	40.2															
phenylacetic acid	68	452															
ethyl decanoate	122	741	4111	5618	2899	4777	3292	1084	3338	3265	3374	2188	3407	1674	2419	1986	1157
decanoic acid	500	2460	3027	2744	2007	2676	1802	696	2160	1796	2057	1845	2523	1337	1620	1191	1446
hexan-1-ol	590	2710	8208	4562	3904	4807	641	9292	2884	3986	7344	2927	5552	2608	3849	2764	2055
phenylacetaldehyde	5.2	21.5															
2-phenylethyl acetate	360	682	481	152	107	210	675		97.7	262	77.5	96.3	234		74.1	69.9	
2-methylpropan-1-ol	19000	33000	2429	2423	2660	1864	2136	4217	1235	4244	3602	4014	3664	3624	4458	4428	1039
3-methylbutanoic acid	490	814															
2-methylpropyl acetate	66	101															
hexanoic acid	4800	4060	2571	5362	802	5207	2494		2981	3841	3802	3458	5528	2722	2370	970	1004
benzaldehyde	150	108		11.3				34.4		160			27.2		139		
butan-1-ol	1900	1120															
butanoic acid	2400	1180															
octan-1-ol	110	44.7	18.0	31.8	29.7	16.2	21.8	95.2	9.9	34.3	29.4	25.9	32.5	40.5	11.5	19.6	4.5
ethyl 2-phenylacetate	155.55	53.8	2.9	5.1		2.4	0.67	15.6	2.5	1.8	3.2	4.3	7.6	8.3	3.6	7.9	2.9
2-methylbutanoic acid	3100	545															
ethyl dodecanoate	3500	269	243	241	158	215	50.9	51	231	176	110	109	279	103	144	38.4	109
propanoic acid	20000	1490															
2-methylpropanoic acid	60000	2180															

		reference no.	158	158	158	158	158	159	160	161	162	163	163	164	164	165	165
		wine sample no.	586	587	588	589	590	591	592	593	594	595	596	597	598	599	600
matrix		mean															
ethanol (%, ALC/VOL)		12.9	13.7	13.3	12.7	11.7	11.8	12.775	13.0	13.5	10.21	12.3	11.9	11.8		11.0	11.2
pH		3.4	3.35	3.70	3.13	3.27	3.42	3.02	3.97	3.55	3.75	2.97	2.98			3.0	3.1
odorant	OTC (µg/kg) in water	mean concentration (µg/L)															
ethyl acetate	5	69100	190758	145920	178886	208000	204609			30673.2		81100	73000			80.9	131.3
acetaldehyde	16	49100															
butane-2,3-dione	1.0	1400															
ethyl hexanoate	1.2	1570		268	1429	1672	965	1478	960.09	123.7	580	3050	2730	1301	338	1021.7	658.2
ethyl 3-methylbutanoate	0.023	27.5	286		55.0		43.2					5.87	6.64	2.0		14.9	13.1
ethyl 2-methylpropanoate	0.089	93.5	155											8.8	49	45.2	27.5
3-methylbutan-1-ol	220	172000	206723	121317	106439	93744	81321	113240		188023.5	87970					157800	107400
3-methylbutyl acetate	7.2	3650	411	3718	6769	7664	4536	7228	7725.37	663.1	1080	9480	7510	1828	318	311.3	1055.6
ethyl butanoate	0.76	374		62.8	318	376	292			128.9		723	647	286	146	221.6	221.7
ethyl 2-methylbutanoate	0.13	42.7												0.87		8.2	5.9
ethyl octanoate	8.7	2460		1384	3015	3797	3188	1695	1589.86	1080.3	440	2270	1880	1407	409	1141.3	760.1
3-methylbutanal	0.50	119															
2-phenylethan-1-ol	140	28700	22479	8293	10234	5873	4998	10662		51802.2	6870						
2-methylpropanal	0.49	36.5															
2-methylbutan-1-ol	1200	70100									229370					22100	14300
dimethyl sulfide	0.30	14.1															
acetic acid	5600	219000	348820	187409	160843	126319	177142										
3-(methylsulfanyl)propan-1-ol	36	1360						132		2287.6	7870					109.0	198.2
3-(methylsulfanyl)propanal	0.43	14.6															
ethyl propanoate	10	295												37	143	290.3	219.3
octanoic acid	190	5580		753	2949	5101	2969	7427		285.6	1210						
3-hydroxybutan-2-one	590	16600						253		8627.0							
2-methylbutanal	1.5	40.2															
phenylacetic acid	68	452						55									
ethyl decanoate	122	741	153	3233	2821	5060	4271	537		80.2	240	626	469	614	273	327.2	100.9
decanoic acid	500	2460	38	1985	2111	3178	2834	2747			280						
hexan-1-ol	590	2710	4399	3616	4971	3035	3497	1224		3164.1	1820					1.4	2.2
phenylacetaldehyde	5.2	21.5															
2-phenylethyl acetate	360	682	42.2	168	426	516	220	428	1722.54	14.4	120	381	283	435	29	67.9	105.7
2-methylpropan-1-ol	19000	33000	9952	5858	1783	2136	1803	19024	33865.40	57765.3	50340					16100	12600
3-methylbutanoic acid	490	814						670		3109.7							
2-methylpropyl acetate	66	101								89.7		170	112	51	16	2.4	5.5
hexanoic acid	4800	4060		1797	2349	6079	5482	5448		1131.3							
benzaldehyde	150	108		67.7	7.9	2.7	89.8										
butan-1-ol	1900	1120						863		2187.2							
butanoic acid	2400	1180						1870									
octan-1-ol	110	44.7	44.1	23.6	11.1	11.2	19.5			11.0							
ethyl 2-phenylacetate	155.55	53.8	14.6	1.2	2.5	0.37	1.7					5.54	5.50	0.43		10.2	28.1
2-methylbutanoic acid	3100	545															
ethyl dodecanoate	3500	269		157	220	326	118			316.2	50	127	83	43		19.4	7.4
propanoic acid	20000	1490						1367									
2-methylpropanoic acid	60000	2180						2766		1214.2							

		reference no.	165	165	165	165	166	166	166	166	167	167	167	167	167	168	168
		wine sample no.	601	602	603	604	605	606	607	608	609	610	611	612	613	614	615
matrix		mean															
ethanol (%, ALC/VOL)		12.9	11.0	11.1	10.5	10.5					11.7	11.7	11.7	11.8	11.8		
pH		3.4	3.0	3.0	3.2	3.3					3.68	3.68	3.68	3.69	3.70		
odorant	OTC (µg/kg) in water	mean concentration (µg/L)															
ethyl acetate	5	69100	117.5	145.7	150.6	210.1	94000	79000	125000	106000	17108.11	27866.57	20653.93	25131.31	21188.48	106740	278620
acetaldehyde	16	49100														195940	77780
butane-2,3-dione	1.0	1400														3530	1870
ethyl hexanoate	1.2	1570	965.3	638.1	742.3	512.4	805	705	772	874	2078.61	1779.86	2028.59	1926.9	1963.6	40.00	60.00
ethyl 3-methylbutanoate	0.023	27.5	8.8	6.5	10.9	7.2											
ethyl 2-methylpropanoate	0.089	93.5	53.9	45.2	46.1	30.1	56	66	69	48						15.44	12.41
3-methylbutan-1-ol	220	172000	107300	88400	135800	106700	125000	139000	116000	99000						421390	321700
3-methylbutyl acetate	7.2	3650	457.8	837.4	545.5	923.9	626	394	592	502	1443.71	1034.39	1341.34	1264.84	1375.9	320.00	635.00
ethyl butanoate	0.76	374	319.8	361.0	196.8	162.3	275	214	258	316						20.00	35.00
ethyl 2-methylbutanoate	0.13	42.7	4.5	3.7	4.9	3.6										3.76	2.54
ethyl octanoate	8.7	2460	1249.4	983.8	1130.0	754.0	1291	1421	1327	1360	4512.04	3922.49	4267.37	3617.63	3737.63	45.00	75.00
3-methylbutanal	0.50	119															
2-phenylethan-1-ol	140	28700					22000	74000	30000	15000	2008.81	2217.82	3578.40	5120.08	3343.82	90100	86820
2-methylpropanal	0.49	36.5															
2-methylbutan-1-ol	1200	70100	19100	15000	15400	10500	25000	31000	25000	19000							
dimethyl sulfide	0.30	14.1					28	12	11	13							
acetic acid	5600	219000														48140	53600
3-(methylsulfanyl)propan-1-ol	36	1360	197.3	240.5	150.6	220.4										1520	1010
3-(methylsulfanyl)propanal	0.43	14.6															
ethyl propanoate	10	295	390.9	200.8	453.4	320.8	86	58	75	63						170.00	310.00
octanoic acid	190	5580					6000	7000	7000	7000	4612.76	5373.03	5877.61	5695.66	5426.34	545.00	515.00
3-hydroxybutan-2-one	590	16600									3618.67	4983.47	5008.13	6021.05	3071.80	15710	25460
2-methylbutanal	1.5	40.2															
phenylacetic acid	68	452															
ethyl decanoate	122	741	289.1	226.1	332.9	121.3	324	335	394	366	1091.22	992.16	943.90	881.99	1008	185.00	440.00
decanoic acid	500	2460					1000	1000	1000	1000	2315.56	2328.23	2199.37	2717.07	2286.71	65.00	85.00
hexan-1-ol	590	2710	0.1	0.9	0.6	1.3	1398	1294	1447	1149	409.07	675	628.33	634.96	540.66	2510	2430
phenylacetaldehyde	5.2	21.5															
2-phenylethyl acetate	360	682	46.5	70.1	69.0	80.7	105	242	178	90	10.58	11.62	11.92	13.14	11.88		
2-methylpropan-1-ol	19000	33000	25300	18700	18300	13600	24000	28000	22000	19000	2064.51	3581.97	3078.39	3827.62	2546.65	108120	99180
3-methylbutanoic acid	490	814									114.04	157.51	172.4	161.64	134.17	3850	2220
2-methylpropyl acetate	66	101	15.7	19.9	18.7	29.1					34.67	50.1	42.1	57.2	30.51	82.48	141.22
hexanoic acid	4800	4060					7000	6000	7000	7000						1270	1070
benzaldehyde	150	108									1145.6	1429.32	1417.02	1673.16	1379.24	1.61	2.29
butan-1-ol	1900	1120									2239.08	3229.38	5597.59	3515	2314.41	410.00	370.00
butanoic acid	2400	1180														595.00	430.00
octan-1-ol	110	44.7									10.11	12.19	9.03	16.86	12.16		
ethyl 2-phenylacetate	155.55	53.8	5.2	11.5	12.1	14.5											
2-methylbutanoic acid	3100	545															
ethyl dodecanoate	3500	269	19.4	10.1	27.2	15.6											
propanoic acid	20000	1490															
2-methylpropanoic acid	60000	2180									3333.79	5131.69	5779.6	7429.69	4386.64	3150	2200

		reference no.	169	169	169	169	170	170	171	171	171	171	172	173	173	174	174
		wine sample no.	616	617	618	619	620	621	622	623	624	625	626	627	628	629	630
matrix		mean															
ethanol (%, ALC/VOL)		12.9	12.7	12.60	13.0	9.60	11.3	12.4					10.7	12.5	12.5	12.9	12.8
pH		3.4					3.48	3.39					2.81	3.51	3.51	3.53	3.53
odorant	OTC (µg/kg) in water	mean concentration (µg/L)															
ethyl acetate	5	69100	100372.34	11061.56	23025.19	16341.50	883.14	743.63	45210	36650	34160	27850	24400	20420	23660		
acetaldehyde	16	49100											2330	7540	13750		
butane-2,3-dione	1.0	1400															
ethyl hexanoate	1.2	1570	418.98	193.75	146.53	47.60	45.14	66.46	172.71	168.44	169.53	153.96	1030	110	470	341.91	406.38
ethyl 3-methylbutanoate	0.023	27.5			10.45	4.76	12.57	6.45	11.50	9.88	8.41	8.70	15.93			7.99	8.41
ethyl 2-methylpropanoate	0.089	93.5			49.29	81.37			183.77	149.37	120.49	149.53				28.96	32.30
3-methylbutan-1-ol	220	172000		4136.22	2661.08	2852.66	1173.94	827.65	174560	170630	155800	171890	178600				
3-methylbutyl acetate	7.2	3650	156.25	232.56	109.96		188.45	509.71	277.90	261.74	226.84	231.73	460			1323.64	1868.69
ethyl butanoate	0.76	374	649.57	162.01	449.73	432.30	30.43		171.03	181.65	169.43	132.20	190	170	660	182.00	192.76
ethyl 2-methylbutanoate	0.13	42.7					12.35	5.11	6.33	5.29	4.62	4.58	8.28			5.71	6.84
ethyl octanoate	8.7	2460	147.6	23.82	19.23	74.87	22.88	34.06	111.29	110.37	127.71	110.44	1400	650	180	353.19	371.07
3-methylbutanal	0.50	119					0.80										
2-phenylethan-1-ol	140	28700	52379.56	58196.64	14640.80	46181.57			17790	15180	16230	16290	53600	67810	52810		
2-methylpropanal	0.49	36.5															
2-methylbutan-1-ol	1200	70100															
dimethyl sulfide	0.30	14.1															
acetic acid	5600	219000											220200	6030	6250		
3-(methylsulfanyl)propan-1-ol	36	1360											1040	980	620		
3-(methylsulfanyl)propanal	0.43	14.6															
ethyl propanoate	10	295		1142.20		2089.54	71.24	27.93	99.58	91.50	93.50	73.19	80			331.88	425.04
octanoic acid	190	5580	12772.54	3299.00	2985.98	9011.51			554.9	667.1	676.1	694.9	20110	10740	10140		
3-hydroxybutan-2-one	590	16600											520	2130	2370		
2-methylbutanal	1.5	40.2					2.47	0.13									
phenylacetic acid	68	452															
ethyl decanoate	122	741			16.65		0.50	3.30	27.00	23.21	28.90	24.57	390	420	410	173.20	186.13
decanoic acid	500	2460	4943.49	4043.81	1211.42	2712.82			63.72	61.90	73.31	72.33	600	2400	1210		
hexan-1-ol	590	2710	11055.22	12491.26	104.82	1583.76			1653.68	1484.86	1731.39	1618.33	920	3400	2320		
phenylacetaldehyde	5.2	21.5															
2-phenylethyl acetate	360	682	294.48	99.42	18.41	76.70			9.86	8.11	7.96	8.29	2370	820	960	108.80	208.39
2-methylpropan-1-ol	19000	33000	44933.06				171.69	78.51					22200	5850	7360		
3-methylbutanoic acid	490	814											1040		240		
2-methylpropyl acetate	66	101			18.69	50.52			100.24	89.97	79.80	96.69	23.6			47.68	64.88
hexanoic acid	4800	4060	4146.54	892.74	514.19	1655.70							4620	7230	7170		
benzaldehyde	150	108			3.24			0.36	15.72	27.56	49.64	26.24		110			
butan-1-ol	1900	1120					2.63	0.86					510	340	510		
butanoic acid	2400	1180											1270	420	450		
octan-1-ol	110	44.7				6.06		0.26	36.76	23.25	24.15	19.40					
ethyl 2-phenylacetate	155.55	53.8			4.36	5.78			1.02	0.78	0.79	0.83				2.33	3.57
2-methylbutanoic acid	3100	545												2360			
ethyl dodecanoate	3500	269														19.37	13.80
propanoic acid	20000	1490															
2-methylpropanoic acid	60000	2180												820	70		

		reference no.	174	174	174	174	174	174	174	174	175	175	175	175	176	176	176
		wine sample no.	631	632	633	634	635	636	637	638	639	640	641	642	643	644	645
matrix		mean															
ethanol (%, ALC/VOL)		12.9	13.1	11.9	11.9	11.9	13.1	13.0	13.0	13.1							
pH		3.4	3.50	3.64	3.65	3.66	3.59	3.60	3.64	3.57							
odorant	OTC (µg/kg) in water	mean concentration (µg/L)															
ethyl acetate	5	69100															
acetaldehyde	16	49100															
butane-2,3-dione	1.0	1400									258.7	309.1	280.8	198.1			
ethyl hexanoate	1.2	1570	481.84	749.16	744.13	1011.69	304.03	355.65	361.26	438.75	710.3	582	574.6	310.7	387	326	793
ethyl 3-methylbutanoate	0.023	27.5	5.48	2.67	3.65	2.43	15.15	9.70	8.87	8.54					36.1	110	20.2
ethyl 2-methylpropanoate	0.089	93.5	26.34	15.83	19.60	15.48	43.67	35.30	36.12	34.79					110	221	69
3-methylbutan-1-ol	220	172000															
3-methylbutyl acetate	7.2	3650	1879.28	4495.44	7075.54	6944.19	1234.67	1259.11	608.74	822.42	573.7	479	390.1	188.6	507	324	3190
ethyl butanoate	0.76	374	196.19	217.90	209.63	223.62	155.40	168.80	179.03	179.23	240.7	225.2	200.7	103.2	177	147	367
ethyl 2-methylbutanoate	0.13	42.7	3.60	1.63	1.95	1.08	11.43	5.51	5.86	6.11	2.5	2.4	2.7	2.5	20.0	66.0	12.1
ethyl octanoate	8.7	2460	401.94	2431.24	2080.01	2949.30	308.39	400.02	491.39	764.69	1678.8	1745.1	1788.4	666.1	479	365	1001
3-methylbutanal	0.50	119															
2-phenylethan-1-ol	140	28700															
2-methylpropanal	0.49	36.5															
2-methylbutan-1-ol	1200	70100															
dimethyl sulfide	0.30	14.1															
acetic acid	5600	219000															
3-(methylsulfanyl)propan-1-ol	36	1360									774.9	613.3	804.5	606.2			
3-(methylsulfanyl)propanal	0.43	14.6															
ethyl propanoate	10	295	260.47	48.62	46.75	29.07	187.28	132.95	135.03	142.30	43.9	46.3	39.5	26.8	91.9	112	111
octanoic acid	190	5580									2383.2	2247.1	2858.2	3393.4			
3-hydroxybutan-2-one	590	16600															
2-methylbutanal	1.5	40.2															
phenylacetic acid	68	452															
ethyl decanoate	122	741	178.06	1199.06	1046.69	1345.35	185.26	267.27	287.91	412.94	511.9	883.5	864.3	270	108	52.9	364
decanoic acid	500	2460									438	509.5	585.6	739.5			
hexan-1-ol	590	2710									1255.6	1122.7	1102.7	756.4			
phenylacetaldehyde	5.2	21.5															
2-phenylethyl acetate	360	682	133.63	763.52	1491.61	1270.16	88.11	62.29	24.09	48.43	49.4	53.3	53.6	42.6	57.5	37.3	311
2-methylpropan-1-ol	19000	33000															
3-methylbutanoic acid	490	814															
2-methylpropyl acetate	66	101	67.05	133.70	189.17	165.28	47.52	48.35	38.75	40.88	4.5	4.1	3.3	1.4	62.7	59.9	126.4
hexanoic acid	4800	4060									4821.8	3411.1	4812.9	3689.1			
benzaldehyde	150	108															
butan-1-ol	1900	1120									394.8	380.6	343.1	226.9			
butanoic acid	2400	1180															
octan-1-ol	110	44.7															
ethyl 2-phenylacetate	155.55	53.8	2.60	1.09	1.53	1.00	2.81	1.33	0.99	1.70					7.51	18.3	3.57
2-methylbutanoic acid	3100	545															
ethyl dodecanoate	3500	269	22.56	120.34	92.37	135.60	9.78	19.47	31.44	39.72	36.8	72	49.9	97.1	2.88	0.59	13.3
propanoic acid	20000	1490															
2-methylpropanoic acid	60000	2180															

		reference no.	176	176	176	177	178	178	178	178	178	178	178	178	178	178	179
		wine sample no.	646	647	648	649	650	651	652	653	654	655	656	657	658	659	660
matrix		mean															
ethanol (%, ALC/VOL)		12.9				14											11.77
pH		3.4				3.35											3.36
odorant	OTC (µg/kg) in water	mean concentration (µg/L)															
ethyl acetate	5	69100				63320	29634.0	67783.0	41981.0				63156.0	80160.0	7720.8	48391.6	28900
acetaldehyde	16	49100				10360	24800.0	5400.0	6700.0	42400.0		4200.0		590000.0	256500.0		
butane-2,3-dione	1.0	1400				1051											
ethyl hexanoate	1.2	1570	1046	1099	1064	638											1000
ethyl 3-methylbutanoate	0.023	27.5	15.2	39.8	32.0	3.6											14.4
ethyl 2-methylpropanoate	0.089	93.5	43.5	108	70.9	66											47.3
3-methylbutan-1-ol	220	172000				139200											179000
3-methylbutyl acetate	7.2	3650	2235	406	1198	3509	1541.1	2661.9	1120.8	1914.7	1494.4		982.8	1029.6	1123.2	1029.6	900
ethyl butanoate	0.76	374	347	310	301	398	546.0	283.4	163.8	434.2	267.8	231.4	110.5	198.9	97.5	244.4	301
ethyl 2-methylbutanoate	0.13	42.7	6.2	18.9	12.4	1.1											
ethyl octanoate	8.7	2460	1516	1420	1352	610	800.0	2400.0	800.0	2080.0	1440.0	728.0	364.0	436.8	436.8	582.4	
3-methylbutanal	0.50	119					30.0										
2-phenylethan-1-ol	140	28700				18840	35640.0	32400.0	9900.0	82260.0	9900.0	45124.0	10846.0	5757.7	18344.0	26780.0	
2-methylpropanal	0.49	36.5					33.3	29.0	21.7	30.8	20.6	30.0	21.8	27.9	19.4	35.3	
2-methylbutan-1-ol	1200	70100															68000
dimethyl sulfide	0.30	14.1															2.0
acetic acid	5600	219000				329000											270000
3-(methylsulfanyl)propan-1-ol	36	1360				559											
3-(methylsulfanyl)propanal	0.43	14.6															
ethyl propanoate	10	295	62.3	102	79.7	896											248
octanoic acid	190	5580				6494											
3-hydroxybutan-2-one	590	16600				2630											
2-methylbutanal	1.5	40.2															
phenylacetic acid	68	452															
ethyl decanoate	122	741	491	387	359	87	205.0			287.0	557.6	639.2	73.8		112.8	56.4	
decanoic acid	500	2460				1678											
hexan-1-ol	590	2710				406	1440.0	960.0	960.0		1680.0	1970.0	1103.8	2049.8	1379.7	3547.8	5600
phenylacetaldehyde	5.2	21.5				0.7											
2-phenylethyl acetate	360	682	170	29.7	133	1926											70
2-methylpropan-1-ol	19000	33000				17300											21700
3-methylbutanoic acid	490	814				321	1788.8	980.4			2064.0	4625.0		1150.0		2150.0	
2-methylpropyl acetate	66	101	55	22.7	21.9	89.5											
hexanoic acid	4800	4060				4836											
benzaldehyde	150	108				4.2											
butan-1-ol	1900	1120				647											490
butanoic acid	2400	1180				2797											
octan-1-ol	110	44.7															
ethyl 2-phenylacetate	155.55	53.8	2.20	6.26	3.44												
2-methylbutanoic acid	3100	545				348											
ethyl dodecanoate	3500	269	18.8	5.84	10.8												
propanoic acid	20000	1490															
2-methylpropanoic acid	60000	2180				1162											

		reference no.	179	179	179	179	180	180	180	180	180	180	180	180	180	180	180
		wine sample no.	661	662	663	664	665	666	667	668	669	670	671	672	673	674	675
matrix		mean															
ethanol (%, ALC/VOL)		12.9	12.87	13.57	14.2	15.50											
pH		3.4	3.50	3.41	3.62	3.46											
odorant	OTC (µg/kg) in water	mean concentration (µg/L)															
ethyl acetate	5	69100	36100	44100	45200	55000											
acetaldehyde	16	49100															
butane-2,3-dione	1.0	1400															
ethyl hexanoate	1.2	1570	1000	1300	1200	1400											
ethyl 3-methylbutanoate	0.023	27.5	13.8	21.8	14.4	27.3											
ethyl 2-methylpropanoate	0.089	93.5	41.3	56.3	42.2	68.2											
3-methylbutan-1-ol	220	172000	185000	187000	205000	205000											
3-methylbutyl acetate	7.2	3650	1000	1300	1300	1800	310.81	870.52	633.62	798.03	700.78	994.15	450.39	530.50	562.38	956.11	331.22
ethyl butanoate	0.76	374	326	386	348	474											
ethyl 2-methylbutanoate	0.13	42.7															
ethyl octanoate	8.7	2460															
3-methylbutanal	0.50	119															
2-phenylethan-1-ol	140	28700															
2-methylpropanal	0.49	36.5															
2-methylbutan-1-ol	1200	70100	79000	77000	89000	88000											
dimethyl sulfide	0.30	14.1	3.0	3.3	4.0	5.3											
acetic acid	5600	219000	200000	270000	300000	370000											
3-(methylsulfanyl)propan-1-ol	36	1360															
3-(methylsulfanyl)propanal	0.43	14.6															
ethyl propanoate	10	295	284	335	357	419											
octanoic acid	190	5580															
3-hydroxybutan-2-one	590	16600															
2-methylbutanal	1.5	40.2															
phenylacetic acid	68	452															
ethyl decanoate	122	741															
decanoic acid	500	2460															
hexan-1-ol	590	2710	4700	4400	4400	3000	3755.92	4260.56	3623.36	4122.34	5082.57	5669.25	3147.09	3621.82	3346.70	4132.32	5893.97
phenylacetaldehyde	5.2	21.5															
2-phenylethyl acetate	360	682	68	114	85	165											
2-methylpropan-1-ol	19000	33000	24100	23600	24500	25700											
3-methylbutanoic acid	490	814															
2-methylpropyl acetate	66	101															
hexanoic acid	4800	4060															
benzaldehyde	150	108															
butan-1-ol	1900	1120	648	778	843	1275											
butanoic acid	2400	1180															
octan-1-ol	110	44.7															
ethyl 2-phenylacetate	155.55	53.8															
2-methylbutanoic acid	3100	545															
ethyl dodecanoate	3500	269															
propanoic acid	20000	1490															
2-methylpropanoic acid	60000	2180															

		reference no.	180	181	182	182	183	184	185	186	187	187	188	188	188	188	188
		wine sample no.	676	677	678	679	680	681	682	683	684	685	686	687	688	689	690
matrix		mean															
ethanol (%, ALC/VOL)		12.9		11.5			12.75			14.3	13.48	13.63	10.86	14.19	10.69	11.79	12.32
pH		3.4		2.93			3.74			3.29	3.27	3.28	4.03	4.15	3.55	3.65	3.89
odorant	OTC (µg/kg) in water	mean concentration (µg/L)															
ethyl acetate	5	69100		32000	61247	82538	53540		25000	11760							
acetaldehyde	16	49100		71000						23130							
butane-2,3-dione	1.0	1400											907	1597	1237	767	1387
ethyl hexanoate	1.2	1570			381.3	832.7	474		167.9	14920	674	871	57	110	137	217	123
ethyl 3-methylbutanoate	0.023	27.5							10.8				3.0	4.3	4.8	12	5.5
ethyl 2-methylpropanoate	0.089	93.5							124.6								
3-methylbutan-1-ol	220	172000		198000				99990.32	140100	184600			200203	208197	206993	217807	252000
3-methylbutyl acetate	7.2	3650	704.41		1156.3	3509.7	1470		860.9	33110			107	437	580	273	393
ethyl butanoate	0.76	374			322.8	1277.8			120.2	890	87.1	103	80	97	93	77	80
ethyl 2-methylbutanoate	0.13	42.7							10.0				2.2	3.2	2.0	7.3	3.5
ethyl octanoate	8.7	2460			214.4	1123.1	477		90.7	54130	1247	763	77	90	90	169	73
3-methylbutanal	0.50	119															
2-phenylethan-1-ol	140	28700		25000			38834	5723.12	24000	11850	5353	7876	40367	65037	58427	66953	97810
2-methylpropanal	0.49	36.5															
2-methylbutan-1-ol	1200	70100						21551.48	1013.1								
dimethyl sulfide	0.30	14.1															
acetic acid	5600	219000								320							
3-(methylsulfanyl)propan-1-ol	36	1360					497			120			1865	1769	1251	3048	4330
3-(methylsulfanyl)propanal	0.43	14.6															
ethyl propanoate	10	295							164.2								
octanoic acid	190	5580					843		49.6	1600	2545	2635	697	503	923	520	553
3-hydroxybutan-2-one	590	16600		24000				86.93					12597	4833	3013	2727	2590
2-methylbutanal	1.5	40.2						936.59									
phenylacetic acid	68	452															
ethyl decanoate	122	741					37		10.2	37420	479	96.7	33	35	42.88	62	30
decanoic acid	500	2460					217		93.3	420	558	381	153	123	193	173	160
hexan-1-ol	590	2710	6072.35				1327	729.86	252.9	2470	268	401	1077	1817	1213	953	1217
phenylacetaldehyde	5.2	21.5															
2-phenylethyl acetate	360	682							68.4	3550	446	27.5	20	68	186	65	113
2-methylpropan-1-ol	19000	33000		19000			11453	11355.80		9080	155	205	31120	32573	37593	28253	27977
3-methylbutanoic acid	490	814								400			77	85	87	78	63
2-methylpropyl acetate	66	101			57.9	21.0			69.8	480							
hexanoic acid	4800	4060					1305			430	694	1019	1150	1180	1667	1067	1270
benzaldehyde	150	108							1.1		11.5	4.91					
butan-1-ol	1900	1120					331			660	15.2	18.4	1873	4213	1127	2797	2913
butanoic acid	2400	1180					534			100	90.5	98.2	390	577	437	447	587
octan-1-ol	110	44.7							15.5	270							
ethyl 2-phenylacetate	155.55	53.8							2.1	50							
2-methylbutanoic acid	3100	545											139	144	117	158	141
ethyl dodecanoate	3500	269								4000	605	123					
propanoic acid	20000	1490															
2-methylpropanoic acid	60000	2180											830	1180	747	873	853

		reference no.	189	189	189	189	189	190	190	191	191	192	193	194	194	194	194
		wine sample no.	691	692	693	694	695	696	697	698	699	700	701	702	703	704	705
matrix		mean															
ethanol (%, ALC/VOL)		12.9						13.7	13.9				13.5				
pH		3.4											3.65				
odorant	OTC (µg/kg) in water	mean concentration (µg/L)															
ethyl acetate	5	69100						84419	44103			38000	62590				
acetaldehyde	16	49100						3061	3128				9650				
butane-2,3-dione	1.0	1400						1071	491			4500	2100				
ethyl hexanoate	1.2	1570	155	284	146.3	20.46	16.37	556	592	5589	2495	320	1110	364	401	339	261
ethyl 3-methylbutanoate	0.023	27.5						9.9	20			10.5		6.7	13.4	14.9	22.3
ethyl 2-methylpropanoate	0.089	93.5	82.3	252	84.48	7.96	15.13	75	66			41		24.6	49.0	53.1	74.6
3-methylbutan-1-ol	220	172000						226992	265404					134000	132000	147000	142000
3-methylbutyl acetate	7.2	3650	136	335		19.27	14.57	5433	1420	5925	6863	572	4690	4470	3403	2557	797
ethyl butanoate	0.76	374	156	438	985.67	17.98	13.73	439	379			147	492.2	376	376	366	380
ethyl 2-methylbutanoate	0.13	42.7						6.3	9.7			8.6					
ethyl octanoate	8.7	2460	199	509	128.32	7.30	5.10	526	216	5400	3702	412	1180	1872	1931	1594	1195
3-methylbutanal	0.50	119															
2-phenylethan-1-ol	140	28700	14170	50983	15412.94	69.84	1826.87	36784	30249	7448	3279		30260	36100	35700	35200	38400
2-methylpropanal	0.49	36.5															
2-methylbutan-1-ol	1200	70100															
dimethyl sulfide	0.30	14.1										5.7					
acetic acid	5600	219000						537000	521000								
3-(methylsulfanyl)propan-1-ol	36	1360	713	723	452.34	15.76	16.00	3855	912				1240				
3-(methylsulfanyl)propanal	0.43	14.6															
ethyl propanoate	10	295	104	924	416.84	21.22	13.23	63	211			60					
octanoic acid	190	5580	520	868	978.15	63.75	29.00	6481	5063				3680	18300	18000	19200	17900
3-hydroxybutan-2-one	590	16600						13713	6993				1870				
2-methylbutanal	1.5	40.2															
phenylacetic acid	68	452															
ethyl decanoate	122	741	90.3	119	92.36		19.94			6075	4503	147	150	638	564	465	281
decanoic acid	500	2460	201	453	268.93	19.38	169.62	1054	457				610	2300	2100	2100	2800
hexan-1-ol	590	2710	743	1578	676.70	29.15	43.84	1714	2083				3260	2509	2858	2863	3108
phenylacetaldehyde	5.2	21.5						181	119								
2-phenylethyl acetate	360	682	81.5	4270	186.59	26.61	36.47	2011	710	3043	1306	37	219.1	13.5	11.1	9.3	6.2
2-methylpropan-1-ol	19000	33000	840	2728	1245.27	25.88	22.99	38018	23058				39730	20500	22400	21900	23500
3-methylbutanoic acid	490	814						712	592			550	990				
2-methylpropyl acetate	66	101						347	55			45	172.4	35.8	25.8	23.9	11.2
hexanoic acid	4800	4060						5989	4890				4550	9300	9600	9400	9800
benzaldehyde	150	108						3.2									
butan-1-ol	1900	1120						1031	1044				1210				
butanoic acid	2400	1180						2874	1972				1780				
octan-1-ol	110	44.7											40.95				
ethyl 2-phenylacetate	155.55	53.8										4.21					
2-methylbutanoic acid	3100	545										232					
ethyl dodecanoate	3500	269										9.4	11.31	35.8	33.0	31.3	29.9
propanoic acid	20000	1490											853.6				
2-methylpropanoic acid	60000	2180						1399	616			1047	2080				

		reference no.	194	194	194	194	194	194	195	196	196	197	197	197	198	199	199
		wine sample no.	706	707	708	709	710	711	712	713	714	715	716	717	718	719	720
matrix		mean															
ethanol (%, ALC/VOL)		12.9							8.77	11.8	12.8				12.4	12.7	13.0
pH		3.4							3.47	3.0	3.1				3.5	3.05	3.01
odorant	OTC (µg/kg) in water	mean concentration (µg/L)															
ethyl acetate	5	69100							4390	52200	121900				59800		
acetaldehyde	16	49100															
butane-2,3-dione	1.0	1400															
ethyl hexanoate	1.2	1570	293	362	300	300	362	262	220	4107.2	3851.4	356	358	277	860	850	2610
ethyl 3-methylbutanoate	0.023	27.5	6.0	4.1	3.5	4.65	8.4	8.9				18.3	14.3	18.5	10	19.2	5.95
ethyl 2-methylpropanoate	0.089	93.5	24.7	24.8	16.0	28.1	38.3	41.1				70.1	47.3	64.8	30		
3-methylbutan-1-ol	220	172000	91000	117000	78000	105000	80000	74800	25310	185600	211200	125000	127000	161000	105000	324000	198000
3-methylbutyl acetate	7.2	3650	3330	4160	3360	7400	2040	2640	2170	14700	14700	1902	1227	2920	3300	3790	13800
ethyl butanoate	0.76	374	354	303	343	342	312	311				380	428	322	380	476	1060
ethyl 2-methylbutanoate	0.13	42.7													3		
ethyl octanoate	8.7	2460	1280	1120	1258	1163	1207	1250	7610	5107.9	5154.0	1271	1471	1848	1830	1200	2530
3-methylbutanal	0.50	119															
2-phenylethan-1-ol	140	28700	53300	38000	42640	57000	16200	19400	11350	14500	24300	40100	25500	42900	49200	39500	15700
2-methylpropanal	0.49	36.5															
2-methylbutan-1-ol	1200	70100													22770		
dimethyl sulfide	0.30	14.1															
acetic acid	5600	219000							300000	18900	16100				500000		
3-(methylsulfanyl)propan-1-ol	36	1360								1990.2	2459.8					2240	775
3-(methylsulfanyl)propanal	0.43	14.6															
ethyl propanoate	10	295													100		
octanoic acid	190	5580	21600	17350	19400	18240	16520	21070	2060	5205.9	2692.4	21600	16500	18100	7900	4820	12200
3-hydroxybutan-2-one	590	16600								3378.5	11000					8.95	13.3
2-methylbutanal	1.5	40.2															
phenylacetic acid	68	452															
ethyl decanoate	122	741	439	454	423	376	614	388	22370	1451.2	1373.7	412	442	442	740	395	905
decanoic acid	500	2460	2670	2250	2740	2020	1760	2260	3870	2877.7	1430.1	2600	1800	2300	2580	1300	3220
hexan-1-ol	590	2710	2860	2543	3273	2301	2748	1426		4041.7	3429.0	2601	3090	3192	780	473	611
phenylacetaldehyde	5.2	21.5															
2-phenylethyl acetate	360	682	12.8	17.4	14.7	19.9	10.9	8.6	200			8.9	6.7	10.9	800	1980	1750
2-methylpropan-1-ol	19000	33000	14800	18800	14600	13700	15100	17200	480	22000	31000	26500	27800	15600	26800	49700	20800
3-methylbutanoic acid	490	814													360		
2-methylpropyl acetate	66	101	33.7	70.3	30.3	95	48.6	33.8				18.0	17.8	20.4	140		
hexanoic acid	4800	4060	10240	8630	10190	8800	8610	9590		1709.8	1033.5	10200	8600	9500	4430	2090	5020
benzaldehyde	150	108								348.6	121.0					20.4	9.04
butan-1-ol	1900	1120								1587.6	3333.5				920	751	1810
butanoic acid	2400	1180													1380		
octan-1-ol	110	44.7								44.2	39.0						
ethyl 2-phenylacetate	155.55	53.8															
2-methylbutanoic acid	3100	545													340		
ethyl dodecanoate	3500	269	120.3	100.4	111.9	97.2	96.06	101.6	2450	222.7	156.4	17.7	17.2	58.0			
propanoic acid	20000	1490								213.3							
2-methylpropanoic acid	60000	2180								2916.4	4975.8				1290		

		reference no.	199	200	200	200	200	201	202	202	203	203	204	204	204	205	205
		wine sample no.	721	722	723	724	725	726	727	728	729	730	731	732	733	734	735
matrix		mean															
ethanol (%, ALC/VOL)		12.9	12.9	13	13.2	14.2	14.0									13.93	13.87
pH		3.4	3.39	4.08	3.98	4.51	4.23									3.20	3.16
odorant	OTC (µg/kg) in water	mean concentration (µg/L)															
ethyl acetate	5	69100		23100	17100	28300	29000		38000	25000			115100.4	98210.7	81586.8	28440	31210
acetaldehyde	16	49100														30480	27130
butane-2,3-dione	1.0	1400		1340	3550	910	2340				2510	280					
ethyl hexanoate	1.2	1570	3820	220	230	270	230	570	722	416			1549.7	1418	1128.7	184	201
ethyl 3-methylbutanoate	0.023	27.5	3.89	7.38	2.04	8.15	6.64				4.51	49.42					
ethyl 2-methylpropanoate	0.089	93.5		30.6	7.83	50.6	56.7						171.4	121.4	191.4		
3-methylbutan-1-ol	220	172000	169000	255000	290000	305000	399000		189000	220000			131990.8	143526.5	182997.3		
3-methylbutyl acetate	7.2	3650	32400	910	1090	1170	1020	3260	3182	1412			1464.9	1624.9	925	256	200
ethyl butanoate	0.76	374	1220	170	190	180	180	366	342	231			461.2	474.1	348.9		
ethyl 2-methylbutanoate	0.13	42.7		1.21	0.81	2.21	3.48				1.73	30.75					
ethyl octanoate	8.7	2460	3170	120	150	140	120	1239	870	424			3705.4	3680.1	3257.2	162	178
3-methylbutanal	0.50	119									43.26	81.44					
2-phenylethan-1-ol	140	28700	19200	53600	45300	70500	71800		53000	51000			14807.4	15614.5	28803.1	12065	10920
2-methylpropanal	0.49	36.5									14.65	40.80					
2-methylbutan-1-ol	1200	70100							49000	55000							
dimethyl sulfide	0.30	14.1															
acetic acid	5600	219000															
3-(methylsulfanyl)propan-1-ol	36	1360	650	2040	2050	3080	2900										
3-(methylsulfanyl)propanal	0.43	14.6									2.24	23.91					
ethyl propanoate	10	295															
octanoic acid	190	5580	14400	1620	1570	1600	1220				1280	560				1047	1222
3-hydroxybutan-2-one	590	16600	5.22	16500	7930	6130	820									860	750
2-methylbutanal	1.5	40.2									17.13	28.21					
phenylacetic acid	68	452		42.9	19.6	55.5	46.4				15.75	58.91					
ethyl decanoate	122	741	824	11.1	9.46	12.7	20.4	593	318	247			1572.4	1505.1	1183.8	380	400
decanoic acid	500	2460	4190	2500	1920	2470	1170									234	190
hexan-1-ol	590	2710	720	1450	1400	1610	1500	1349	574	1019			2542.8	1290.1	1874.4	379	318
phenylacetaldehyde	5.2	21.5									8.74	23.00					
2-phenylethyl acetate	360	682	3880	100	48.9	109	51.1	466	563	131						249	300
2-methylpropan-1-ol	19000	33000	14400	61300	67200	85100	90900		25000	16000							
3-methylbutanoic acid	490	814		1410	1780	2120	2380										
2-methylpropyl acetate	66	101		46.6	41.8	57.7	51.9										
hexanoic acid	4800	4060	4560	2320	2090	2210	2070				3120	1100				802	740
benzaldehyde	150	108	7.58					2					11.4	23.8	52.7		
butan-1-ol	1900	1120	1370	2620	2750	2490	2590										
butanoic acid	2400	1180		1030	930	1650	1200										
octan-1-ol	110	44.7															
ethyl 2-phenylacetate	155.55	53.8															
2-methylbutanoic acid	3100	545		153	91.6	198	110										
ethyl dodecanoate	3500	269						37	28	16							
propanoic acid	20000	1490		2.86	2.33	4.32	2.58										
2-methylpropanoic acid	60000	2180		1350	1020	2320	1530										

		reference no.	205	206	206	206	207	208	208	208	209	209	209	210	211	212	213
		wine sample no.	736	737	738	739	740	741	742	743	744	745	746	747	748	749	750
matrix		mean															
ethanol (%, ALC/VOL)		12.9	13.88					11.1	11.2	10.9	12.7			14.5			11.5
pH		3.4	3.17					3.02	3.29	3.23	3.17			3.79			3.38
odorant	OTC (µg/kg) in water	mean concentration (µg/L)															
ethyl acetate	5	69100	28750				73400	28800	39400	26900	38600	56700	67400		20490	32900	56400
acetaldehyde	16	49100	26980								179000	162000	158000	53.7	93960	78300	
butane-2,3-dione	1.0	1400															89
ethyl hexanoate	1.2	1570	190	740	1030	580	1000	415	721	532	655	813	787		40	768	295
ethyl 3-methylbutanoate	0.023	27.5					9.2								10	2.38	
ethyl 2-methylpropanoate	0.089	93.5					71.9										
3-methylbutan-1-ol	220	172000		45100	55100	58000	140000	193600	175900	178200	133000	127000	129000		124440	152000	
3-methylbutyl acetate	7.2	3650	269	890	790	200	2140	1315	4371	1825	1225	120	135	825	680	1330	749
ethyl butanoate	0.76	374					504	187	318	214	81.5	98.3	91.1	123	20	93.6	250
ethyl 2-methylbutanoate	0.13	42.7					10.4										
ethyl octanoate	8.7	2460	190	140	920	640	1520	509	910	623	1106	698	763		40	2520	111
3-methylbutanal	0.50	119															
2-phenylethan-1-ol	140	28700	10480	27900	38100	47600	45500		32300	36700	5343	7135	8394	5970	56830	13400	12000
2-methylpropanal	0.49	36.5															
2-methylbutan-1-ol	1200	70100					26500	48500	45400	46000	71700	28700	29000			63000	
dimethyl sulfide	0.30	14.1															
acetic acid	5600	219000					386000							250000			
3-(methylsulfanyl)propan-1-ol	36	1360		930	980	1010										15.1	1030
3-(methylsulfanyl)propanal	0.43	14.6															
ethyl propanoate	10	295					190										
octanoic acid	190	5580	1292	5440	8060	6060	9300				2490	2630	2560		3630	2610	2180
3-hydroxybutan-2-one	590	16600	700												5570		
2-methylbutanal	1.5	40.2															
phenylacetic acid	68	452														32.2	
ethyl decanoate	122	741	360	155	253	43.1	677	235	319	239	454	80.2	95.7	200		851	
decanoic acid	500	2460	248	1320	2170	670	3460				557	410	389		1390	1080	745
hexan-1-ol	590	2710	356				2430	665	616	1212	206	315	317		940	233	666
phenylacetaldehyde	5.2	21.5															
2-phenylethyl acetate	360	682	185	350	1190	380	404	233	534	190	363	40.8	21.5	78.4	80	715	23
2-methylpropan-1-ol	19000	33000					28000	39400	34100	25500	31600	31200	31800		140	30400	19600
3-methylbutanoic acid	490	814					492								130	328	595
2-methylpropyl acetate	66	101					56.9								180		
hexanoic acid	4800	4060	806		1990	1880	5100				630	1060	930		5150	941	6470
benzaldehyde	150	108						1	2	1	9.62	3.77	5.10			2.43	111
butan-1-ol	1900	1120					810				16.7	21.7	19.3		740	13.1	1300
butanoic acid	2400	1180		600	170	220	2240				84.5	101	89.2		10760	9.68	5060
octan-1-ol	110	44.7												10.4		4.19	
ethyl 2-phenylacetate	155.55	53.8														6.79	
2-methylbutanoic acid	3100	545					236										732
ethyl dodecanoate	3500	269					150	20	15	23	638	134	125			31.3	
propanoic acid	20000	1490					1368										
2-methylpropanoic acid	60000	2180		2200	1680	1600	493								50	9.00	

		reference no.	213	213	213	214	214	214	214	215	215	215	216	217	218	218	219
		wine sample no.	751	752	753	754	755	756	757	758	759	760	761	762	763	764	765
matrix		mean															
ethanol (%, ALC/VOL)		12.9	11.4	13.4	12.8								12.2				
pH		3.4	3.42	3.52	3.45								3.59				
odorant	OTC (µg/kg) in water	mean concentration (µg/L)															
ethyl acetate	5	69100	48100	46500	51800								51002				
acetaldehyde	16	49100															
butane-2,3-dione	1.0	1400		172													
ethyl hexanoate	1.2	1570	401	303	739	813	668	1068	766	1200	800	1440	899		810	1500	
ethyl 3-methylbutanoate	0.023	27.5								54	34	7.7			27.4	43	
ethyl 2-methylpropanoate	0.089	93.5			121	7	7	6	5	420	170	40			107	119.6	
3-methylbutan-1-ol	220	172000				361410	312905	95489	418424						83200	62100	
3-methylbutyl acetate	7.2	3650	1140	1600	3050	1379	2022	3271	2454	2030	290	550	1260		17	11	
ethyl butanoate	0.76	374	312	180	460	210	240	240	270	310	200	240			270	760	
ethyl 2-methylbutanoate	0.13	42.7								35	26	5.4					
ethyl octanoate	8.7	2460	140	71	298	3103	3005	5005	4446	830	530	1180	667		1140	1500	
3-methylbutanal	0.50	119															
2-phenylethan-1-ol	140	28700	15400	19600	16000	28654	15312	25216	13890	18000	32800	31000	930		5700	3400	
2-methylpropanal	0.49	36.5															
2-methylbutan-1-ol	1200	70100															
dimethyl sulfide	0.30	14.1															
acetic acid	5600	219000															
3-(methylsulfanyl)propan-1-ol	36	1360		1240	563	4301	1338	3686	5009				40				
3-(methylsulfanyl)propanal	0.43	14.6												17.0			
ethyl propanoate	10	295															
octanoic acid	190	5580	5910		8440								1008		7850	7100	
3-hydroxybutan-2-one	590	16600															4768
2-methylbutanal	1.5	40.2															
phenylacetic acid	68	452															
ethyl decanoate	122	741			59	299	588	311	1072	110	118	180	101		530	52	
decanoic acid	500	2460											565		4000	1760	
hexan-1-ol	590	2710	1150	1900	1620	5876	4094	4812	7003				502		417	1370	
phenylacetaldehyde	5.2	21.5												42.3			39.8
2-phenylethyl acetate	360	682	51	59	138	127	180	232	317	150	23	36	92		4.866	2.66	
2-methylpropan-1-ol	19000	33000	19800	21900	21800	31012	18938	13834	34661				17790				
3-methylbutanoic acid	490	814	461	735	679												
2-methylpropyl acetate	66	101			74					152	35	60					
hexanoic acid	4800	4060	6180	5820	7340								865		11700	10700	
benzaldehyde	150	108		242		31	21	44	26								67.4
butan-1-ol	1900	1120	1100	1500	1360								1610				
butanoic acid	2400	1180	3360	3620	2840								307				
octan-1-ol	110	44.7			12												
ethyl 2-phenylacetate	155.55	53.8								7.7	8.3	5.9					
2-methylbutanoic acid	3100	545	689	1120	1040												
ethyl dodecanoate	3500	269				28	13	12	31						23		
propanoic acid	20000	1490															
2-methylpropanoic acid	60000	2180															

		reference no.	219	220	221	222	223	223	224	225	226	226	226	226	226	226	226
		wine sample no.	766	767	768	769	770	771	772	773	774	775	776	777	778	779	780
matrix		mean															
ethanol (%, ALC/VOL)		12.9				11.5											
pH		3.4				2.82											
odorant	OTC (µg/kg) in water	mean concentration (µg/L)															
ethyl acetate	5	69100		61270					70000								
acetaldehyde	16	49100				43100											
butane-2,3-dione	1.0	1400															
ethyl hexanoate	1.2	1570		1120	308				1200		1990	1910	1560	1570	1820	1620	1730
ethyl 3-methylbutanoate	0.023	27.5							14								
ethyl 2-methylpropanoate	0.089	93.5			19.0				43								
3-methylbutan-1-ol	220	172000		205020	249750	291500			12000								
3-methylbutyl acetate	7.2	3650		260	930				2000		3370	3670	3570	4140	6810	4130	3360
ethyl butanoate	0.76	374			274				506								
ethyl 2-methylbutanoate	0.13	42.7							7.1								
ethyl octanoate	8.7	2460		1180	397				1800	2420	14780	11170	8950	10220	8640	7570	11910
3-methylbutanal	0.50	119															
2-phenylethan-1-ol	140	28700		20140		114900			24000	92900							
2-methylpropanal	0.49	36.5															
2-methylbutan-1-ol	1200	70100							21000								
dimethyl sulfide	0.30	14.1					2.5	2.0									
acetic acid	5600	219000				130000			320000								
3-(methylsulfanyl)propan-1-ol	36	1360					2900	3054									
3-(methylsulfanyl)propanal	0.43	14.6															
ethyl propanoate	10	295							382								
octanoic acid	190	5580				6900			8100	6920							
3-hydroxybutan-2-one	590	16600	7943														
2-methylbutanal	1.5	40.2															
phenylacetic acid	68	452															
ethyl decanoate	122	741			52				1200	950							
decanoic acid	500	2460				3700			2900								
hexan-1-ol	590	2710			1790				2600								
phenylacetaldehyde	5.2	21.5	51.7														
2-phenylethyl acetate	360	682		40	37.2				174	270							
2-methylpropan-1-ol	19000	33000		14420	25950	40300			20000								
3-methylbutanoic acid	490	814							279								
2-methylpropyl acetate	66	101							50								
hexanoic acid	4800	4060				4900			5100	3690							
benzaldehyde	150	108	51.3		226												
butan-1-ol	1900	1120			1520				1000								
butanoic acid	2400	1180							1800								
octan-1-ol	110	44.7															
ethyl 2-phenylacetate	155.55	53.8								40							
2-methylbutanoic acid	3100	545							2900								
ethyl dodecanoate	3500	269			10.5				500								
propanoic acid	20000	1490							1900								
2-methylpropanoic acid	60000	2180							452								

		reference no.	226	226	226	226	226	226	226	226	227	228	228	229	229	230	231
		wine sample no.	781	782	783	784	785	786	787	788	789	790	791	792	793	794	795
matrix		mean															
ethanol (%, ALC/VOL)		12.9									14.2	11.1	12.2	13.6	12.0		
pH		3.4									3.53	2.90	3.05	3.08	2.95		
odorant	OTC (µg/kg) in water	mean concentration (µg/L)															
ethyl acetate	5	69100									56790			37	32.5		80530
acetaldehyde	16	49100												61500	40500		
butane-2,3-dione	1.0	1400															
ethyl hexanoate	1.2	1570	2180	1470	2450	1540	1660	1890	1400	1450	680	780	680	614	478	326.42	
ethyl 3-methylbutanoate	0.023	27.5									10						
ethyl 2-methylpropanoate	0.089	93.5									40	90	50				
3-methylbutan-1-ol	220	172000									279686			223500	210000	88036	113600
3-methylbutyl acetate	7.2	3650	3510	3250	5850	2940	4210	5270	3430	2090	1069	510	1180	2886	202	754.17	
ethyl butanoate	0.76	374									259	1160	900	246	204		
ethyl 2-methylbutanoate	0.13	42.7									7	200	190				
ethyl octanoate	8.7	2460	10530	9140	14190	12060	10310	11150	9600	7610	604	670	670	839	548	340.84	
3-methylbutanal	0.50	119															
2-phenylethan-1-ol	140	28700									59869	34020	63160	37900	45700	16727	56890
2-methylpropanal	0.49	36.5															
2-methylbutan-1-ol	1200	70100									63412						
dimethyl sulfide	0.30	14.1									18						
acetic acid	5600	219000															
3-(methylsulfanyl)propan-1-ol	36	1360									184	1120	3730				
3-(methylsulfanyl)propanal	0.43	14.6															
ethyl propanoate	10	295									369						
octanoic acid	190	5580									2531			4475	3399		
3-hydroxybutan-2-one	590	16600															
2-methylbutanal	1.5	40.2															
phenylacetic acid	68	452															
ethyl decanoate	122	741									131			172	113		
decanoic acid	500	2460									505			989	670		
hexan-1-ol	590	2710										750	930	500	700	642.08	3450
phenylacetaldehyde	5.2	21.5															
2-phenylethyl acetate	360	682									50		430	354	261	233.73	
2-methylpropan-1-ol	19000	33000									36359	18340	11490	44500	42500		
3-methylbutanoic acid	490	814									1069			708	1109		
2-methylpropyl acetate	66	101									24		280	109	75		
hexanoic acid	4800	4060									2889			2480	2084		
benzaldehyde	150	108															28
butan-1-ol	1900	1120									1442						
butanoic acid	2400	1180															
octan-1-ol	110	44.7														11.10	
ethyl 2-phenylacetate	155.55	53.8															
2-methylbutanoic acid	3100	545									1265						
ethyl dodecanoate	3500	269									62						
propanoic acid	20000	1490															
2-methylpropanoic acid	60000	2180									1442						

		reference no.	231	231	231	231	231	231	232	232	232	232	232	233	233	234	234
		wine sample no.	796	797	798	799	800	801	802	803	804	805	806	807	808	809	810
matrix		mean															
ethanol (%, ALC/VOL)		12.9							8.5								
pH		3.4							3.56								
odorant	OTC (µg/kg) in water	mean concentration (µg/L)															
ethyl acetate	5	69100	95990	69640	33230	97520	99170	12230	7000	7500	8000	8000	6000				
acetaldehyde	16	49100															
butane-2,3-dione	1.0	1400															
ethyl hexanoate	1.2	1570												1300	930	52900	78400
ethyl 3-methylbutanoate	0.023	27.5												10	10		
ethyl 2-methylpropanoate	0.089	93.5															
3-methylbutan-1-ol	220	172000	209680	163680	143870	214460	106370	74970								286000	309000
3-methylbutyl acetate	7.2	3650							600	620	900	430	900	1480	1140	148000	288000
ethyl butanoate	0.76	374												380	210		
ethyl 2-methylbutanoate	0.13	42.7															
ethyl octanoate	8.7	2460							17	40	26	25	60	1240	960	180000	201000
3-methylbutanal	0.50	119															
2-phenylethan-1-ol	140	28700	85950	66920	81120	75010	44260	50220	13700	28000	30100	29000	31000	21200	35200	46800	24600
2-methylpropanal	0.49	36.5															
2-methylbutan-1-ol	1200	70100															
dimethyl sulfide	0.30	14.1															
acetic acid	5600	219000															
3-(methylsulfanyl)propan-1-ol	36	1360							150	410	700	600	700				
3-(methylsulfanyl)propanal	0.43	14.6															
ethyl propanoate	10	295															
octanoic acid	190	5580							450	610	626	590	890	4560	2510	17400	20200
3-hydroxybutan-2-one	590	16600															
2-methylbutanal	1.5	40.2															
phenylacetic acid	68	452															
ethyl decanoate	122	741							200	144	220	120	170	310	360		
decanoic acid	500	2460							400	900	940	530	820	1270	930	2000	2400
hexan-1-ol	590	2710	4160	3380	3190	4040	3680	3050	900	1900	2600	1900	2100	400	400	8100	7900
phenylacetaldehyde	5.2	21.5															
2-phenylethyl acetate	360	682							9000	7300	12800	2000	10000	140	240		
2-methylpropan-1-ol	19000	33000							3200	1900	2400	2000	3000				
3-methylbutanoic acid	490	814												290	230		
2-methylpropyl acetate	66	101															
hexanoic acid	4800	4060							270	520	671	710	750	2470	1210	5500	3300
benzaldehyde	150	108	46	45	52	41	48	38									
butan-1-ol	1900	1120															
butanoic acid	2400	1180												900	560		
octan-1-ol	110	44.7															
ethyl 2-phenylacetate	155.55	53.8															
2-methylbutanoic acid	3100	545															
ethyl dodecanoate	3500	269															
propanoic acid	20000	1490															
2-methylpropanoic acid	60000	2180												50	50		

		reference no.	234	235	235	236	236	236	236	237	237	237	237	237	237	237	237
		wine sample no.	811	812	813	814	815	816	817	818	819	820	821	822	823	824	825
matrix		mean															
ethanol (%, ALC/VOL)		12.9		12.2	12.0												
pH		3.4		3.59	3.57												
odorant	OTC (µg/kg) in water	mean concentration (µg/L)															
ethyl acetate	5	69100		51002	67476	6100	7200	9600	53300								
acetaldehyde	16	49100				1600			2200								
butane-2,3-dione	1.0	1400															
ethyl hexanoate	1.2	1570	21700	899	3962	800	800	900	100								
ethyl 3-methylbutanoate	0.023	27.5															
ethyl 2-methylpropanoate	0.089	93.5															
3-methylbutan-1-ol	220	172000	310000			305100	318400	313800	380300								
3-methylbutyl acetate	7.2	3650	43200	1260	5170	1500	1600	1200	500								
ethyl butanoate	0.76	374				500	400	500	400								
ethyl 2-methylbutanoate	0.13	42.7				400	500	300	5400								
ethyl octanoate	8.7	2460	26200	667	2871	900	800	900	300								
3-methylbutanal	0.50	119															
2-phenylethan-1-ol	140	28700	37200	930	6564	17200	15900	100	23700	34816	42854	30230	32386	41553	27429	65381	43734
2-methylpropanal	0.49	36.5															
2-methylbutan-1-ol	1200	70100															
dimethyl sulfide	0.30	14.1															
acetic acid	5600	219000															
3-(methylsulfanyl)propan-1-ol	36	1360		40	96												
3-(methylsulfanyl)propanal	0.43	14.6															
ethyl propanoate	10	295															
octanoic acid	190	5580	6400	1008	6046	13400	5800	12300	3200								
3-hydroxybutan-2-one	590	16600															
2-methylbutanal	1.5	40.2															
phenylacetic acid	68	452				200		300		322.95	86.94	565.57	893.61	7.04	516.23	250.39	
ethyl decanoate	122	741		101	496	1000	300		800								
decanoic acid	500	2460		565	2474	1400	600	400	100								
hexan-1-ol	590	2710	7900	502	1078												
phenylacetaldehyde	5.2	21.5								56.46	57.51	59.80	62.66	55.32	56.98	58.88	56.42
2-phenylethyl acetate	360	682		92	235					126.60	120.23	126.64	135.01	170.33	123.15	131.70	128.51
2-methylpropan-1-ol	19000	33000		17790	37321	19500	28800	17000	44700								
3-methylbutanoic acid	490	814															
2-methylpropyl acetate	66	101															
hexanoic acid	4800	4060	2500	865	3377	9100	1900	8600	600								
benzaldehyde	150	108															
butan-1-ol	1900	1120		1610	1720												
butanoic acid	2400	1180		307	542												
octan-1-ol	110	44.7															
ethyl 2-phenylacetate	155.55	53.8								52.59	16.19	89.90	112.70	9.44	76.63	40.39	17.17
2-methylbutanoic acid	3100	545															
ethyl dodecanoate	3500	269															
propanoic acid	20000	1490															
2-methylpropanoic acid	60000	2180															

		reference no.	237	237	237	237	237	237	237	237	237	237	238	238	238	238	239
		wine sample no.	826	827	828	829	830	831	832	833	834	835	836	837	838	839	840
matrix		mean															
ethanol (%, ALC/VOL)		12.9															
pH		3.4															
odorant	OTC (µg/kg) in water	mean concentration (µg/L)															
ethyl acetate	5	69100															2300
acetaldehyde	16	49100															1500
butane-2,3-dione	1.0	1400											1930	990	960	1120	
ethyl hexanoate	1.2	1570															1300
ethyl 3-methylbutanoate	0.023	27.5															
ethyl 2-methylpropanoate	0.089	93.5															
3-methylbutan-1-ol	220	172000															
3-methylbutyl acetate	7.2	3650															2300
ethyl butanoate	0.76	374															
ethyl 2-methylbutanoate	0.13	42.7															
ethyl octanoate	8.7	2460															1200
3-methylbutanal	0.50	119															
2-phenylethan-1-ol	140	28700	39362	39909	53585	48062	49572	20131	25645	37717	41712	45369					14000
2-methylpropanal	0.49	36.5															
2-methylbutan-1-ol	1200	70100															
dimethyl sulfide	0.30	14.1															
acetic acid	5600	219000															
3-(methylsulfanyl)propan-1-ol	36	1360															190
3-(methylsulfanyl)propanal	0.43	14.6															
ethyl propanoate	10	295															
octanoic acid	190	5580															7100
3-hydroxybutan-2-one	590	16600															
2-methylbutanal	1.5	40.2															
phenylacetic acid	68	452	71.14	354.68	6993.27	4165.45	2105.46	573.03	1435.78	400.02							
ethyl decanoate	122	741															510
decanoic acid	500	2460															2000
hexan-1-ol	590	2710															660
phenylacetaldehyde	5.2	21.5	62.80	53.77	79.07	65.37	63.10	54.00	47.07	58.25	48.38	67.32					
2-phenylethyl acetate	360	682	134.73	116.64	154.17	166.04	139.79	99.74	116.94	249.74	128.94	117.51					
2-methylpropan-1-ol	19000	33000															3200
3-methylbutanoic acid	490	814															
2-methylpropyl acetate	66	101															
hexanoic acid	4800	4060															3700
benzaldehyde	150	108															
butan-1-ol	1900	1120															
butanoic acid	2400	1180															350
octan-1-ol	110	44.7															
ethyl 2-phenylacetate	155.55	53.8	42.00	97.80	32.76	12.10	13.59	63.09	150.25	64.68	18.30	35.67					
2-methylbutanoic acid	3100	545															
ethyl dodecanoate	3500	269															
propanoic acid	20000	1490															
2-methylpropanoic acid	60000	2180															

		reference no.	239	239	240	241	241	242	242	242	243	243	243	243	243	243	243
		wine sample no.	841	842	843	844	845	846	847	848	849	850	851	852	853	854	855
matrix		mean															
ethanol (%, ALC/VOL)		12.9				11.0	10.7										
pH		3.4				3.23	2.98										
odorant	OTC (µg/kg) in water	mean concentration (µg/L)															
ethyl acetate	5	69100	2300	700		32000	69000	63500	75400	78800	62280	44320	35650	88460	27950	24100	18470
acetaldehyde	16	49100	4000	9500				41000	35600	34100							
butane-2,3-dione	1.0	1400															
ethyl hexanoate	1.2	1570	1100	1030	589	879	921	1000	890	460							
ethyl 3-methylbutanoate	0.023	27.5			17												
ethyl 2-methylpropanoate	0.089	93.5															
3-methylbutan-1-ol	220	172000			182200	267000	492000				149890	52470	160650	75260	137380	58210	102270
3-methylbutyl acetate	7.2	3650	1900	1100	1680	2650	3186	1610	1190	1040	2770	820	1220	960	520	680	460
ethyl butanoate	0.76	374			254	168	230	260	270	150	270		760	20	330	110	120
ethyl 2-methylbutanoate	0.13	42.7															
ethyl octanoate	8.7	2460	1700	1000	830	650	535	1390	1100	480	1200	170	1340	320	1190	590	1150
3-methylbutanal	0.50	119															
2-phenylethan-1-ol	140	28700	22000	27000	43770	44000	126000	18600	22300	23400	12260	72280	14890	71730	47380	26950	35250
2-methylpropanal	0.49	36.5															
2-methylbutan-1-ol	1200	70100			39030						125180	372950	102110	476330	177300	149870	153490
dimethyl sulfide	0.30	14.1															
acetic acid	5600	219000			415700						43480	4390	13660	56810	22630	10260	10540
3-(methylsulfanyl)propan-1-ol	36	1360	320	301.3													
3-(methylsulfanyl)propanal	0.43	14.6															
ethyl propanoate	10	295				109	294										
octanoic acid	190	5580	10000	12500		706	663	5220	5170	2260	10270	1090	13750	1310	12040	5920	9870
3-hydroxybutan-2-one	590	16600				15000	8300	2900	6700	19100							
2-methylbutanal	1.5	40.2															
phenylacetic acid	68	452															
ethyl decanoate	122	741	680	420	198	54		280	270	100	290	10	270	20	300	150	330
decanoic acid	500	2460	2200	2500		144	309	1030	1250	600	910	40	3280	70	2990	1600	2380
hexan-1-ol	590	2710	1600	1780	1870	322	817	1330	1780	1720	850	1920	400	1810	1040	570	680
phenylacetaldehyde	5.2	21.5															
2-phenylethyl acetate	360	682			103	553	951	200	170	120	240	70	150	80	260	130	280
2-methylpropan-1-ol	19000	33000	2500	2680	29720	131000	229000	27400	29900	42100							
3-methylbutanoic acid	490	814															
2-methylpropyl acetate	66	101				154	194	50	40	30							
hexanoic acid	4800	4060	5700	7000	4430	2500	2532	4510	4020	2310	3620	580	3160	300	4540	2360	3630
benzaldehyde	150	108			8		14								16		
butan-1-ol	1900	1120			1150	492	1334										
butanoic acid	2400	1180	530	710				4950	5020	4770						128	177
octan-1-ol	110	44.7															
ethyl 2-phenylacetate	155.55	53.8										20		20	20	30	
2-methylbutanoic acid	3100	545															
ethyl dodecanoate	3500	269															
propanoic acid	20000	1490															
2-methylpropanoic acid	60000	2180						1730	1780	1940							

		reference no.	243	243	243	243	243	243	243	243	243	243	243	243	243	243	243
		wine sample no.	856	857	858	859	860	861	862	863	864	865	866	867	868	869	870
matrix		mean															
ethanol (%, ALC/VOL)		12.9															
pH		3.4															
odorant	OTC (µg/kg) in water	mean concentration (µg/L)															
ethyl acetate	5	69100	39630	23740	28090	66630	78040	84590	51150	69390	48640	29180	85770	100340	321400	35450	38850
acetaldehyde	16	49100															
butane-2,3-dione	1.0	1400															
ethyl hexanoate	1.2	1570															
ethyl 3-methylbutanoate	0.023	27.5															
ethyl 2-methylpropanoate	0.089	93.5															
3-methylbutan-1-ol	220	172000	99520	155030	155590	76520	116570	152260	54470	83930	177300	113040	89110	84910	158440	53280	104750
3-methylbutyl acetate	7.2	3650	2480	530	680	1220	990	1740	660	1300	2720	760	740	730	890	930	580
ethyl butanoate	0.76	374	590	60	50	10	400	390		570	330	450	600	640	210	300	390
ethyl 2-methylbutanoate	0.13	42.7															
ethyl octanoate	8.7	2460	1120	1040	1090	350	610	890	280	410	1480	790	390	330	1430	970	950
3-methylbutanal	0.50	119															
2-phenylethan-1-ol	140	28700	13710	19680	20800	31250	39570	31090	40730	91400	8630	11720	51680	51540	28890	20690	28500
2-methylpropanal	0.49	36.5															
2-methylbutan-1-ol	1200	70100	137930	65420	91480	187980	281210	250480	209470	383660	111150	75360	407710	347600	136350	207830	200180
dimethyl sulfide	0.30	14.1															
acetic acid	5600	219000	18250	23020	23440	8760	63290	48430	3880	47180	15880	24160	60830	74620	11290	28020	13430
3-(methylsulfanyl)propan-1-ol	36	1360															
3-(methylsulfanyl)propanal	0.43	14.6															
ethyl propanoate	10	295															
octanoic acid	190	5580	12950	6890	10630	2650	4120	6210	2290	2610	13880	8610	1390	1820	11980	8290	7630
3-hydroxybutan-2-one	590	16600															
2-methylbutanal	1.5	40.2															
phenylacetic acid	68	452															
ethyl decanoate	122	741	250	720	190	20	110	130	20	20	360	130	50	40	410	250	260
decanoic acid	500	2460	2750	750	1920	290	610	60	240	60	3480	1430	30	50	3310	1840	2000
hexan-1-ol	590	2710	430	900	580	1620	2650	680	1300	1980	650	1180	1990	1780	653	750	870
phenylacetaldehyde	5.2	21.5															
2-phenylethyl acetate	360	682	420	180	140	100	90	380	60	150	180	100	70	60	210	150	80
2-methylpropan-1-ol	19000	33000															
3-methylbutanoic acid	490	814															
2-methylpropyl acetate	66	101															
hexanoic acid	4800	4060	3850	3120	2640	1650	1030	1340	801	261	4140	4350	661	576	3100	670	1530
benzaldehyde	150	108			9						20			20			
butan-1-ol	1900	1120															
butanoic acid	2400	1180	88	39					380			120				104	
octan-1-ol	110	44.7															
ethyl 2-phenylacetate	155.55	53.8		50	90							10	10	30			
2-methylbutanoic acid	3100	545															
ethyl dodecanoate	3500	269															
propanoic acid	20000	1490															
2-methylpropanoic acid	60000	2180															

		reference no.	243	244	244	245	245	245	245	245	245	246	246	246	246	246	246
		wine sample no.	871	872	873	874	875	876	877	878	879	880	881	882	883	884	885
matrix		mean															
ethanol (%, ALC/VOL)		12.9				12	12	12.5	12	12	12.5						
pH		3.4										3.39	3.22	3.34	3.33	3.25	3.17
odorant	OTC (µg/kg) in water	mean concentration (µg/L)															
ethyl acetate	5	69100	25850	45000	56000												
acetaldehyde	16	49100															
butane-2,3-dione	1.0	1400															
ethyl hexanoate	1.2	1570		190	180							824	755	670	884	602	424
ethyl 3-methylbutanoate	0.023	27.5										6	1	3	3	30	135
ethyl 2-methylpropanoate	0.089	93.5										95	31	35	91	361	328
3-methylbutan-1-ol	220	172000	86550														
3-methylbutyl acetate	7.2	3650	390	368	273												
ethyl butanoate	0.76	374	500	460	480							141	100	143	178	162	80
ethyl 2-methylbutanoate	0.13	42.7										4	3	4	3	7	21
ethyl octanoate	8.7	2460	780	210	200							862	728	578	981	506	218
3-methylbutanal	0.50	119															
2-phenylethan-1-ol	140	28700	18890	25000	16000												
2-methylpropanal	0.49	36.5															
2-methylbutan-1-ol	1200	70100	119310														
dimethyl sulfide	0.30	14.1															
acetic acid	5600	219000	27910			119000	400000	380000	215000	290000	370000						
3-(methylsulfanyl)propan-1-ol	36	1360															
3-(methylsulfanyl)propanal	0.43	14.6															
ethyl propanoate	10	295															
octanoic acid	190	5580	7630	1000	880												
3-hydroxybutan-2-one	590	16600															
2-methylbutanal	1.5	40.2															
phenylacetic acid	68	452															
ethyl decanoate	122	741	140	70	60							408	357	355	511	153	150
decanoic acid	500	2460	1600	270	320												
hexan-1-ol	590	2710	620	3350	3090												
phenylacetaldehyde	5.2	21.5															
2-phenylethyl acetate	360	682	90	289	57	528	405	212	324	673	314						
2-methylpropan-1-ol	19000	33000		41000	64000												
3-methylbutanoic acid	490	814		840	600												
2-methylpropyl acetate	66	101															
hexanoic acid	4800	4060	1370	1110	1000												
benzaldehyde	150	108	10														
butan-1-ol	1900	1120		6000	1000												
butanoic acid	2400	1180		1580	2600												
octan-1-ol	110	44.7															
ethyl 2-phenylacetate	155.55	53.8	30														
2-methylbutanoic acid	3100	545															
ethyl dodecanoate	3500	269		520	380												
propanoic acid	20000	1490															
2-methylpropanoic acid	60000	2180		1300	1280												

		reference no.	247	247	247	247	248	248	248	249	249	249	249	249	250	251	251
		wine sample no.	886	887	888	889	890	891	892	893	894	895	896	897	898	899	900
matrix		mean															
ethanol (%, ALC/VOL)		12.9															
pH		3.4															
odorant	OTC (µg/kg) in water	mean concentration (µg/L)															
ethyl acetate	5	69100	45610	66510	67240	65440	61200	40900	53700						22570		
acetaldehyde	16	49100	40640	44930	25430	25640									35150		
butane-2,3-dione	1.0	1400															
ethyl hexanoate	1.2	1570	980	1310	1330	1240	550	720	240	2700	2400	1900	2300	2800	390		
ethyl 3-methylbutanoate	0.023	27.5															
ethyl 2-methylpropanoate	0.089	93.5															
3-methylbutan-1-ol	220	172000	95830	97590	81630	87540	47700	50130	3720	48200	46200	39600	48900	37200	85510		
3-methylbutyl acetate	7.2	3650	2120	6870	4380	6130	1520	60	70	12500	10500	13200	12700	10600	1430		
ethyl butanoate	0.76	374					160	10	30						380		
ethyl 2-methylbutanoate	0.13	42.7														103	95
ethyl octanoate	8.7	2460	870	850	930	960	380	900	150						820		
3-methylbutanal	0.50	119													14		
2-phenylethan-1-ol	140	28700	20000	11800	8400	9800	18600	9740	8400	4100	3900	4000	4300	3700	9960		
2-methylpropanal	0.49	36.5															
2-methylbutan-1-ol	1200	70100	25010	21340	16650	17790									15380		
dimethyl sulfide	0.30	14.1															
acetic acid	5600	219000															
3-(methylsulfanyl)propan-1-ol	36	1360	520	690	260	260	2110	310									
3-(methylsulfanyl)propanal	0.43	14.6															
ethyl propanoate	10	295															
octanoic acid	190	5580	5260	6730	9050	7610	3200	1690	1920	300	292	301	201	202	2220		
3-hydroxybutan-2-one	590	16600															
2-methylbutanal	1.5	40.2															
phenylacetic acid	68	452															
ethyl decanoate	122	741					360	340	50								
decanoic acid	500	2460	2170	2380	2290	3690	670	600	1620						5620		
hexan-1-ol	590	2710	881.4	451.2	499.1	502.3	710	190	440	2770	2650	3170	2970	2870	230		
phenylacetaldehyde	5.2	21.5															
2-phenylethyl acetate	360	682					380	320	20	3700	2800	3600	3500	3500	120		
2-methylpropan-1-ol	19000	33000	17400	17860	14780	13900									20930		
3-methylbutanoic acid	490	814					120	100							460		
2-methylpropyl acetate	66	101															
hexanoic acid	4800	4060	3870	5240	6240	5470									3070		
benzaldehyde	150	108													20		
butan-1-ol	1900	1120															
butanoic acid	2400	1180	380	360	420	370									450		
octan-1-ol	110	44.7															
ethyl 2-phenylacetate	155.55	53.8	100	120	340	330											
2-methylbutanoic acid	3100	545															
ethyl dodecanoate	3500	269															
propanoic acid	20000	1490															
2-methylpropanoic acid	60000	2180	1550	1260	780	760	2690		1100						420		

		reference no.	251	251	252	252
		wine sample no.	901	902	903	904
matrix		mean				
ethanol (%, ALC/VOL)		12.9				
pH		3.4				
odorant	OTC (µg/kg) in water	mean concentration (µg/L)				
ethyl acetate	5	69100				
acetaldehyde	16	49100				
butane-2,3-dione	1.0	1400				
ethyl hexanoate	1.2	1570				
ethyl 3-methylbutanoate	0.023	27.5				
ethyl 2-methylpropanoate	0.089	93.5				
3-methylbutan-1-ol	220	172000				
3-methylbutyl acetate	7.2	3650				
ethyl butanoate	0.76	374				
ethyl 2-methylbutanoate	0.13	42.7	38.3	6.9	71.6	40.8
ethyl octanoate	8.7	2460				
3-methylbutanal	0.50	119				
2-phenylethan-1-ol	140	28700				
2-methylpropanal	0.49	36.5				
2-methylbutan-1-ol	1200	70100			58600	18200
dimethyl sulfide	0.30	14.1				
acetic acid	5600	219000				
3-(methylsulfanyl)propan-1-ol	36	1360				
3-(methylsulfanyl)propanal	0.43	14.6				
ethyl propanoate	10	295				
octanoic acid	190	5580				
3-hydroxybutan-2-one	590	16600				
2-methylbutanal	1.5	40.2			20.41	7.10
phenylacetic acid	68	452				
ethyl decanoate	122	741				
decanoic acid	500	2460				
hexan-1-ol	590	2710				
phenylacetaldehyde	5.2	21.5				
2-phenylethyl acetate	360	682				
2-methylpropan-1-ol	19000	33000				
3-methylbutanoic acid	490	814				
2-methylpropyl acetate	66	101				
hexanoic acid	4800	4060				
benzaldehyde	150	108				
butan-1-ol	1900	1120				
butanoic acid	2400	1180				
octan-1-ol	110	44.7				
ethyl 2-phenylacetate	155.55	53.8				
2-methylbutanoic acid	3100	545			722	433
ethyl dodecanoate	3500	269				
propanoic acid	20000	1490				
2-methylpropanoic acid	60000	2180				

Table S7. Matrix Compositions of the Beer and Wine Aroma Base Models

beer	ethanol (%, ALC/VOL)	5.0[a]
	pH	4.5[a]
	citric acid (mg/L)	150[b]
	glycerol (g/L)	1.29[b]
	carbonation (g/L)	~4.7
wine	ethanol (%, ALC/VOL)	12.9[a]
	pH	3.4[a]
	tartaric acid (mg/L)	1330[b]
	glycerol (g/L)	7.47[b]
	carbonation (g/L)	0

[a] Arithmetic mean of individual values resulting from the literature survey. [b] Concentration data were approximated from Souci, S. W.; Fachmann, W.; Kraut, H. *Food Composition and Nutrition Tables* , 8th edition; Medpharm Scientific Publishers: Stuttgart, Germany, 2016; pp 1151–1169.

Sensory analysis of alcoholic beverage model solutions: INSTRUCTIONS

Dear assessor,

Please orthonasally evaluate the aroma of the test sample(s) and then mark a position on the blue ruler considering the following points:

- The leftmost position indicates that you perceive the sample as 100% beer-like; this position is defined by sample B consisting of a beer aroma model solution
- The rightmost position indicates that you perceive the sample as 100% wine-like; this position is defined by sample W consisting of a wine aroma model solution
- The position in the middle of the ruler indicates that you cannot decide whether the sample is more beer-like or more wine-like
- A position in the left half of the ruler indicates that you perceive the sample as more beer-like than wine-like; the more left the position, the more beer-like you perceive the sample
- A position in the right half of the ruler indicates that you perceive the sample as more wine-like than beer-like; the more right the position, the more wine-like you perceive the sample

Example 1: You perceive the sample as 100% wine-like

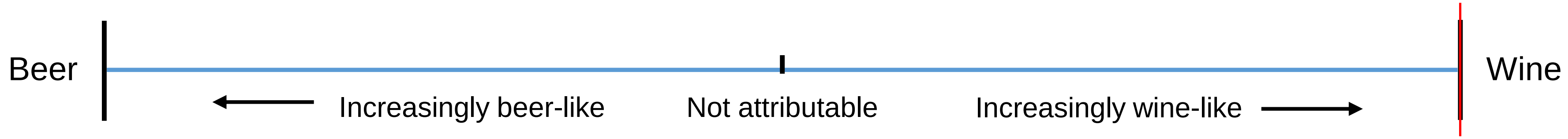

Example 2: You perceive the sample as somewhat more wine-like than beer-like

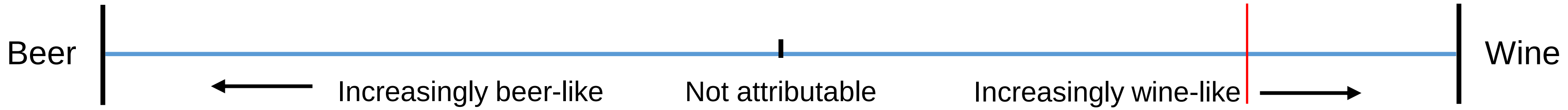

Example 3: You are unable to decide whether the sample is more beer-like or more wine-like

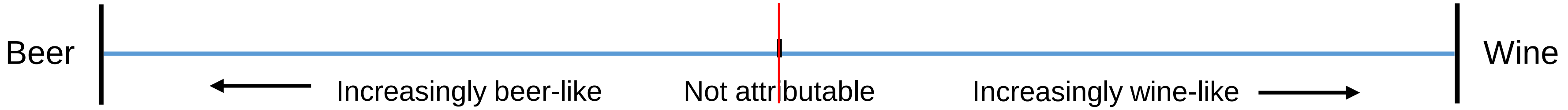

Sensory analysis

Assessor: Date:

Please orthonasally evaluate the aroma of the test samples according to the instruction provided separately.

Sample 1

Beer Wine

← Increasingly beer-like Not attributable Increasingly wine-like →

Sample 2

Beer Wine

← Increasingly beer-like Not attributable Increasingly wine-like →

Sample 3

Beer Wine

← Increasingly beer-like Not attributable Increasingly wine-like →
